# Investigation of the N^C Ligand Effects on Emission Characteristics in a Series of Bis-Metalated [Ir(N^C)_2_(N^N)]^+^ Complexes

**DOI:** 10.3390/molecules28062740

**Published:** 2023-03-17

**Authors:** Zohreh Hendi, Daria O. Kozina, Vitaly V. Porsev, Kristina S. Kisel, Julia R. Shakirova, Sergey P. Tunik

**Affiliations:** 1 Institute of Chemistry, Saint-Petersburg State University, Universitetskii pr. 26, 198504 St. Petersburg, Russia; 2 Department of Chemistry, Sharif University of Technology, Tehran P.O. Box 11155-3516, Iran

**Keywords:** iridium complexes, phosphorescence, structural characterization, photophysical characteristics, ligand effects

## Abstract

A series of bis-metalated phosphorescent [(N^C)_2_Ir(bipyridine)]^+^ complexes with systematic variations in the structure and electronic characteristics of the N^C ligands were synthesized and characterized by using elemental analysis, mass spectrometry, NMR spectroscopy and X-ray crystallography. Investigation of the complexes’ spectroscopic properties together with DFT and TD DFT calculations revealed that metal-to-ligand charge transfer (MLCT) and intraligand (LC) transition play key roles in the generation of emissive triplet states. According to the results of theoretical studies, the ^3^LC excited state is more accurate to consider as an intraligand charge transfer process (ILCT) between N- and C-coordinated moieties of the N^C chelate. This hypothesis is completely in line with the trends observed in the experimental absorption and emission spectra, which display systematic bathochromic shifts upon insertion of electron-withdrawing substituents into the N-coordinated fragment. An analogous shift is induced by expansion of the aromatic system of the C-coordinated fragment and insertion of polarizable sulfur atoms into the aromatic rings. These experimental and theoretical findings extend the knowledge of the nature of photophysical processes in complexes of this type and provide useful instruments for fine-tuning of their emissive characteristics.

## 1. Introduction

Bis-metalated iridium complexes containing complementary diimine chelates, [Ir(N^C)_2_(N^N)]^+^, are very well known as efficient emitters and have attracted considerable attention in the last decade due to their potential applicability in many areas of modern photonic technologies such as electroluminescence devices [1,2], photochemical energy conversion [3], upconversion emitters [4,5], photoelectrochemical analytics [6] and luminescent microscopy [7,8,9,10,11]. Emitters of this type are photostable and display high emission quantum yields and a very wide range of emission wavelengths from blue [12,13] to the near-infrared region. [14,15,16,17] The key emitter characteristics, including emission wavelength, are determined by the character of the emissive excited state, which in complexes of this type is commonly represented by metal disturbed intraligand (IL) [18,19], metal-to-ligand (MLCT) and ligand-to-ligand charge-transfer (LLCT) transitions or their mixture [20,21,22,23], depending on the nature of the metalated and diimine ligands. It is quite evident that the characteristics of all of these excited states for the complexes of the [Ir(N^C)_2_(N^N)]^+^ type depend on the properties of the ligands’ aromatic systems, the π* orbitals of which play an active role in excited state generation. Development of calculation methods facilitated easier interpretation of the obtained experimental data, but it is still very hard to predict *a priori* emission parameters of novel chromophores. This is why investigation of the photophysics and identification of general trends of change in emission characteristics depending on the structure and properties of ligand aromatic systems play an important role in fine-tuning the emission parameters of this class of iridium complexes.

In the current communication, we present the synthesis, characterization, photophysical and theoretical study of a series of [Ir(N^C)_2_(N^N)]^+^ complexes with substantial variations in the properties of metalating ligands. The complexes contain bipyridine as the N^N ligand, whereas the N^C chelates are built up from the 4-carboxy-quinoline N-functionality with H, F and NO_2_ substituents in 6-position and various aromatic systems (phenyl, 4-Br-phenyl, thienyl and benzothienyl), which serve as metalating C-coordinated functions. These variations in the structure and properties of the N- and C-coordinating functions were aimed at the study of their effects on the spectroscopic and photophysical properties of iridium complexes, thus making possible the targeted synthesis of emitters with predetermined photophysical characteristics. Analysis of the photophysical data and results of density-functional theory (DFT) and time-dependent (TD) DFT calculations for these complexes made it possible to draw some conclusions, which are helpful for fine-tuning of the complexes’ photophysical characteristics.

## 2. Results and Discussion

### 2.1. Synthesis and Characterization

**Scheme 1:**
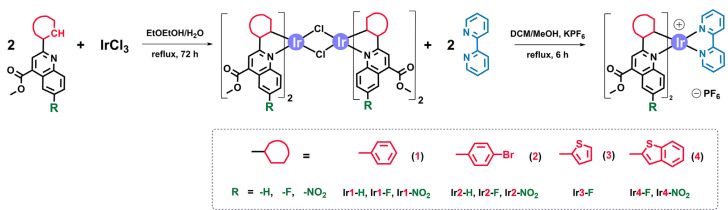
Synthesis of [Ir(N^C)_2_(N^N)]PF_6_ complexes.

The metalating N^C proligands were prepared using a method described in the literature (Scheme S1, Supplementary Materials) [24,25]; details on the synthesis and spectroscopic characteristics of the obtained compounds are given in Electronic Supporting Information (ESI), Part S1, Figures S1–S8. The iridium complexes were obtained in good yield (50–80%) using a standard two-stage synthetic procedure shown in Scheme 1.

**Figure 1:**
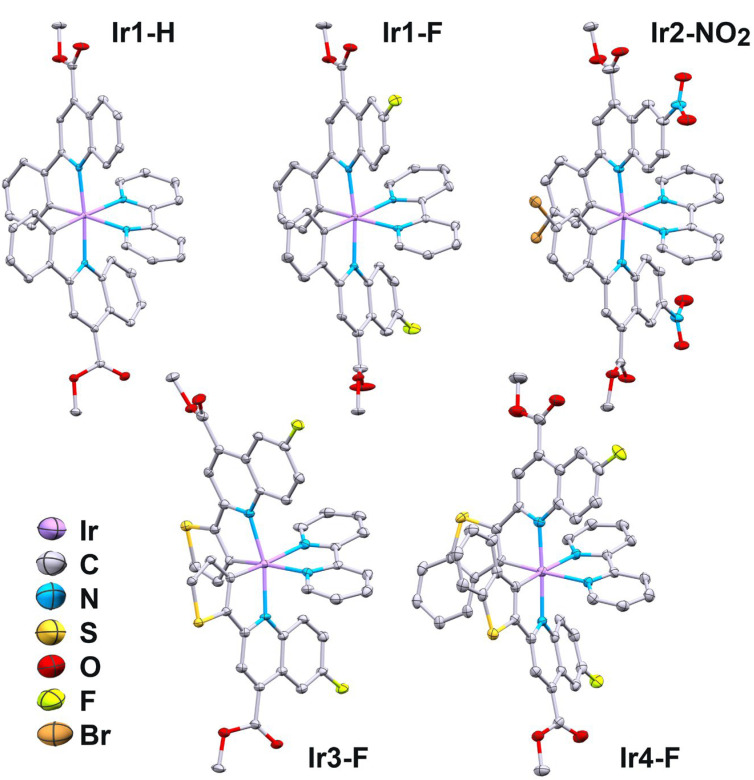
Perspective views of Ir1-H, Ir1-F, Ir2-NO_2_, Ir3-F, Ir4-F. Thermal ellipsoids are shown at the 40% probability level. Hydrogen atoms and counterions (PF_6_) are omitted for clarity.

Five compounds (Ir1-H, Ir1-F, Ir2-NO_2_, Ir3-F, Ir4-F) gave single crystals suitable for XRD study, and their structure in the solid state was established by crystallographic analysis. Key structural parameters of these molecules are summarized in Table S1; perspective views are shown in Figure 1.

Ligands in the coordination sphere of these complexes form a pseudo-octahedral environment at the iridium ion, with the nitrogen and carbon atoms of the N^C ligands disposed in *trans*- and *cis*-positions, respectively. This structural pattern is typical for complexes of this sort; bond angles and lengths in the coordination octahedron are not exceptional and fall in the range characteristic for the cationic [Ir(N^C)_2_(N^N)]^+^ complexes [26,27,28]. The observed deviations from the ideal octahedral geometry around the iridium center are due to short bite angles of the N^C ligands, which are about 80° in these structures. The aromatic systems of the N^C ligands display a slightly non-planar configuration, due to the effect of both short bite angle and intramolecular nonbonding C–H···N contacts between the protons of quinoline fragments of the N^C ligands and the nitrogen atoms of bipyridine ligand (H···N distances fall in the range 2.22–2.41 Å, Table S1).

All obtained substances were characterized by elemental analysis, mass spectrometry and 1D proton and ^1^H-^1^H COSY NMR spectroscopy, see Experimental and Figures S9–S33 (Supplementary Materials). Electro-spray ionization, positive mode (ESI+) mass spectra of these complexes display the major signals of the {Ir(N^C)_2_(N^N)}^+^ molecular ions with the isotopic distributions that fit completely their molecular stoichiometry. The combination of 1D and 2D COSY proton NMR spectra made possible detailed assignment of the signals observed in these spectra (Figures S9–S24). The ^1^H spectroscopic patterns display the proton resonances corresponding to coordinated bipyridine and two equivalent N^C ligands that are completely compatible with the molecular architecture found in the solid state.

### 2.2. Photophysics

**Figure 2:**
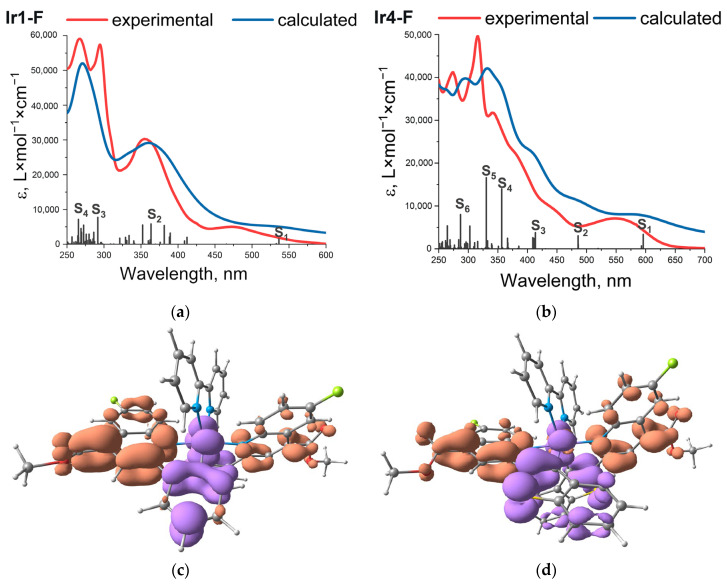
Experimental (red) and calculated (blue) absorption spectra of Ir1-F (**a**) and Ir4-F (**b**); oscillator strengths of electronic transitions are shown with vertical bars along wavelength axis. NTOs of the Ir1-F (**c**) and Ir4-F (**d**) lowest energy (S_0_→S_1_) transitions: decrease and increase in electron density are given in violet and terracotta colors, respectively.

**Figure 3:**
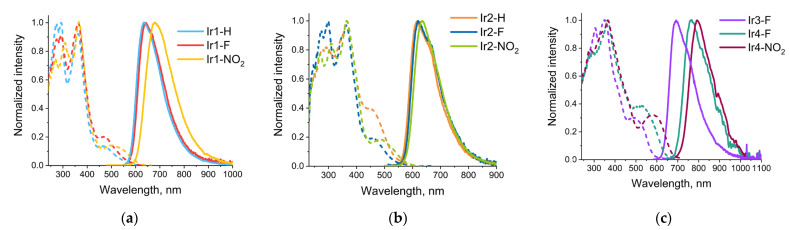
Normalized excitation (dashed line) and emission (solid line) spectra of iridium complexes: (**a**) Ir1-H, Ir1-F, Ir1-NO_2_; (**b**) Ir2-H, Ir2-F, Ir2-NO_2_; (**c**) Ir3-F, Ir4-F, Ir4-NO_2_.

All complexes studied were luminescent in dichloromethane solution at room temperature. Absorption, excitation, and emission spectra are shown in Figure S34, Figure 2 and Figure 3, respectively; photophysical data are summarized in Table 1.

Electronic spectra of the studied complexes displayed strong absorption (ε ≈ 3–6 × 10^4^ M^−1^cm^−1^) in the region 250–300 nm, which may be assigned to the π→π* transitions associated with aromatic systems of the ligands. Several absorption bands were observed between 300 and 400 nm (ε ≈ 2–3 × 10^4^ M^−1^cm^−1^), related to the mixed excited states composed of the ligand centered (^1^LC), ligand-to-ligand (^1^LLCT), and metal-to-ligand charge-transfer (^1^MLCT) transitions. The weak (ε < 10^4^ M^−1^cm^−1^) lowest energy bands/shoulders below 400 nm may be described in terms of mixed ^1^LC/^1^MLCT character. The above description of the absorption spectra was confirmed by TD DFT calculations, which yielded very good agreement with experimental data, see Figure 2 and Figures S35–S43. The calculations also provided assignments of the characters of main transitions based on the values of interfragment charge transfer (IFCT) summarized in Tables S2–S19, where visualization of the transition is also shown by natural transition orbitals (NTOs). An example of the calculated absorption spectra for the complexes Ir1-F and Ir4-F is shown in Figure 2 together with the NTOs corresponding to the lowest energy transitions.

Thorough analysis of the NTO characters indicates that, in fact, the dominating ^1^LC contribution into the lowest S_0_→S_1_ transition should be more accurately described as an intraligand charge transfer process (^1^ILCT), as it essentially consists of electron transition between the metalated aromatic system and N-coordinated carboxy-quinoline functionality of the N^C chelate (Figure 2). This conclusion seems very natural, taking into account a certain excess of electronic density on the metalated fragment and electron-withdrawing substituents (COOMe, F, NO_2_) at the N-coordinated moiety. A systematic bathochromic shift of the lowest energy absorption, both in the experimental and calculated spectra (see Table 1 and results of calculation in ESI), upon increase in electronegativity of the carboxy-quinoline substituents (H→F→NO_2_) in the Ir1-# series is completely in line with the suggested interpretation of the excited state character.

Observed emission of the studied complexes (Figure 3 and Table 1) demonstrates large Stokes shift (150–200 nm), excited state lifetimes in microsecond domain and strong intensity and lifetime dependence on oxygen concentration that indicate emission origin from excited triplet state, i.e., phosphorescence. The bands maxima for the complexes containing phenyl- and thienyl-metalated functions span the interval between 614 and 690 nm to give intense orange-red emission with the quantum yield (Φ) 20–80% in degassed dichloromethane solution, Table 1. It is worth noting that emitters from Ir2-# series display the highest QYs, first of all, due to the lowest k_nr_ constants among the complexes studied. In this series, the Ir2-NO_2_ gives a record QY value of 81% in degassed solution that is a result of the lowest k_nr_ and highest k_r_; the latter may be explained by a very high contribution of metal orbitals in the emissive T_1_→S_0_ transition (Table S13) and, consequently, higher magnitude of spin-orbital constant, which facilitates the spin-forbidden transition. The complexes with benzothienyl moiety coordinated to iridium center (Ir4-F and Ir4-NO_2_) display a large bathochromic shift of emission band maxima to the NIR region, up to 792 nm in the case of Ir4-NO_2,_ that is typical for the ligands containing this metalated aromatic system [11,26,29,30]. Emission efficiency for both complexes demonstrated extremely strong reduction that did not allow reliable determination of the quantum yield values. A sharp drop in emission quantum yield is quite typical for the phosphorescence NIR emitters and can be explained in terms of the “energy gap law” [31,32].

**Figure 4:**
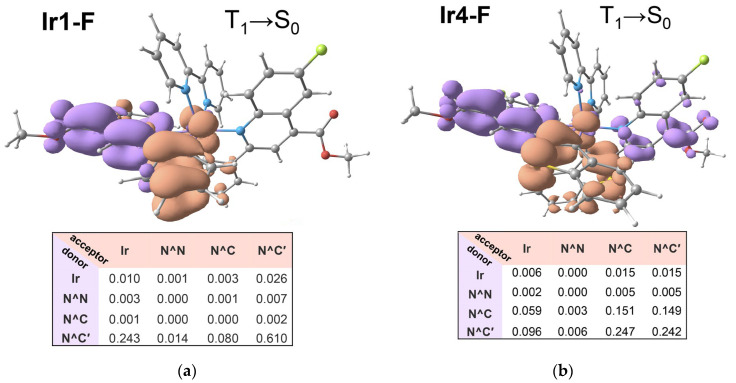
NTO of the emission (T_1_→S_0_) transition in the complexes Ir1-F (**a**) and Ir4-F (**b**): decrease and increase in electron density are shown in violet and terracotta colors, respectively, and corresponding interfragment charge transfer (IFCT) tables showing contribution of charge transfers between certain molecular fragments: iridium ion (Ir), N^C and N^N ligands.

The emission bands for all complexes were nearly structureless, showing indistinct shoulders at the long wavelength tail of the bands that indicated a major contribution of charge transfer character into emissive excited states. TD DFT analysis of the T_1_→S_0_ transitions (Figure 4, Tables S2–S19) also confirmed this assignment, showing the major contribution of the charge transfer processes (N^C→Ir, N^C→N^C′, intra-N^C) into the triplet excited state for the chromophores under investigation (see Figure 4 and Tables S3–S19) that was essentially similar to the character of the lowest excited singlet (S_1_) state.

**Figure 5:**
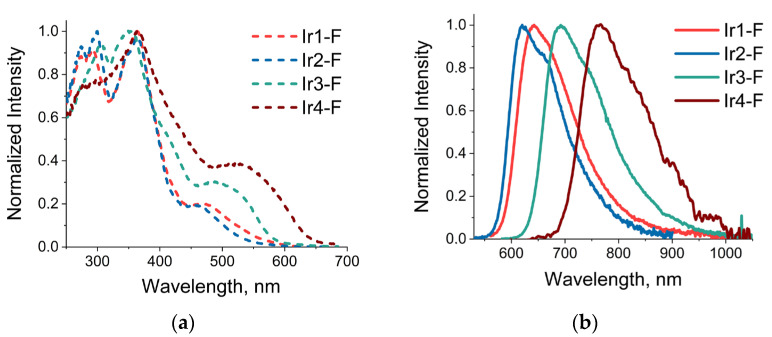
Normalized excitation (**a**) and emission spectra (**b**) of complexes Ir1-F, Ir2-F, Ir3-F, Ir4-F.

The similarity in the nature of the S_1_ and T_1_ states was also reflected in the shifts of the corresponding energies upon increase in electronegativity of the substituents in the carboxy-quinoline aromatic system. Comparison of the experimental emission band maxima in two series of compounds (Ir1-H, Ir1-F, Ir1-NO_2_) and (Ir2-H, Ir2-F, Ir2- NO_2_) indicated a small but systematic bathochromic shift in the H→F→NO_2_ sequence. Rather small shift values pointed to a weak effect of these substituents on the energy of emission because of their remote location and lower contribution of the related NTOs. On the contrary, expansion of the aromatic system at the C-coordinated function and substitution of the phenyl ring (Ir1-# and Ir2-# series) for the thienyl and benzotheinyl moieties, which contained polarizable sulfur atoms, resulted in contraction of the emission energy gap to clearly visible band maxima shifts (see Table 1 and Figure 5), cf. the data for Ir3-F vs. Ir4-F, Ir3-NO_2_ vs. Ir4-NO_2_, and the Ir#-F sequence, # = 2(Br-Ph)→1(Ph)→3(thienyl)→4(benzotheinyl). The observed contraction stemmed from the increase in the energy of the ground state orbitals located at the C-coordinated fragment of the chelate (electron acceptor in the T_1_→S_0_ transition), whereas the composition and structure of the N-coordinated moiety (electron donor in emissive transition) was kept unchanged. The results of the HOMO energy calculations confirmed the observed trend, giving the following magnitudes for the Ir#-F sequence mentioned above: −6.29, −6.05, −6.01 and −5.81 eV, respectively.

## 3. Materials and Methods

### 3.1. General Comments

Preparation of the ligands is described in Electronic Supporting Information, Part S1. All reagents were purchased from commercial sources and were used as received. ^1^H and ^1^H-^1^H COSY NMR spectra were recorded on a Bruker Avance 400 MHz instrument. Chemical shifts are reported in parts per million (ppm) and referenced to residual peaks of deuterated solvents (Acetone-*d_6_* (2.05 ppm), or DMSO-*d_6_* (2.50 ppm)). High-resolution mass spectra (HRMS) were measured on a Bruker Daltonik MaXis ESIQTOF instrument in the ESI^+^ mode. Microanalyses were carried out by using Euro EA3028-HT.

### 3.2. Preparation of the Complexes

General procedure: Cyclometalated Ir(III) µ-chloro-bridged dimers of general formula (N^C)_2_Ir(µ-Cl)_2_Ir(N^C)_2_ were synthesized by the method suggested by Nonoyama, which included refluxing IrCl_3_ × nH_2_O with 2–2.5 equivalents of cyclometalating ligand in a 3:1 mixture of 2-ethoxyethanol and water [33,34,35].

Synthesis of [(N^C)_2_Ir(N^N)]^+^ (N^N: 2,2′-bipyridine) complexes. General procedure: [(N^C)_2_IrCl]_2_ complex (0.01 mmol), 0.03 mmol of 2,2′-bipyridine (Sigma Aldrich, St. Louis, MO, USA), and 0.1 mmol of potassium hexafluorophosphate were refluxed under an inert gas environment in the blend of dichloromethane and methanol (1:1) for 12–16 h. After cooling to room temperature, the mixture was filtered, and the solvent was evaporated under reduced pressure. The residue was flash chromatographed utilizing a silica/dichloromethane column to yield ca. 50–80% of the crude [Ir(N^C)_2_(N^N)]PF_6_ product after solvent evaporation and drying. The target coordination compounds were recrystallized by a diffusion of diethyl ether into a dichloromethane or dichloromethane/acetone solution of the corresponding Ir(III) complexes at room temperature. Note that the numbering schemes used in description of proton NMR spectra are shown in Figures S9–S24.

Complex Ir1-H (yield 78%): red crystalline material. Crystals of Ir1-H are prism, C_44_H_32_IrN_4_O_4_∙PF_6_, *P* –1, a = 9.08970(10), b = 10.89890(10), c = 19.3715(2) Å, α = 84.4690(10), β = 78.9330(10), γ = 87.4290(10)°, V = 1873.95(3) Å^3^, Z = 2, R_1_ = 0.0324, CCDC 2242684. ^1^H NMR (400 MHz, DMSO-*d_6_*), ppm: 8.86 (s, 1H, H_9_), 8.52 (d, 1H, ^3^J = 8.79, H_10_), 8.43 (d, 1H, ^3^J = 7.88, H_1_), 8.36 (d, 1H, ^3^J = 5.76, H_5_), 8.33 (d, 1H, ^3^J = 7.58, H_8_), 8.13 (td, 1H, ^3^J = 8.48, H_11_), 7.71 (t, 1H, ^3^J = 6.67, H_12_), 7.59 (d, 1H, ^3^J = 8.48, H_13_), 7.53 (d, 1H, ^3^J = 7.58, H_6_), 7.24 (t, 1H, ^3^J = 7.58, H_2_), 7.16 (t, 1H, ^3^J = 7.58, H_7_), 6.59 (td, 1H, ^3^J = 7.58, H_3_), 6.65 (d, 1H, ^3^J = 7.58, H_4_), 4.13 (s, 3H, H_14_). HRMS (ESI^+^) *m*/*z*: 873.1963, calculated for C_44_H_32_IrN_4_O_4_^+^: 873.2047. Elemental analysis; calculated for C_44_H_32_IrN_4_O_4_PF_6_ C, 51.92; H, 3.17; N, 5.50, experimental C, 51.89; H, 3.17; N, 5.52.

Complex Ir2-H (yield 68%): dark red crystalline material. ^1^H NMR (400 MHz, DMSO-*d_6_*), ppm: 8.88 (s, 1H, H_8_), 8.54 (dd, 1H, ^3^J = 9.20, H_1_), 8.47 (d, 1H, ^3^J = 5.47, H_9_), 8.43 (d, 1H, ^3^J = 7.95, H_12_), 8.32 (d, 1H, ^3^J = 8.45, H_7_), 8.16 (td, 1H, ^3^J = 8.45, H_10_), 7.75 (td, 1H, ^3^J = 6.21, H_11_), 7.57 (m, 2H, H_3,4_), 7.48 (dd, 1H, ^3^J = 8.27, H_6_), 7.22 (td, 1H, ^3^J = 7.81, H_2_), 6.83 (d, 1H, ^3^J = 2.15, H_5_), 4.13 (s, 3H, H_13_). HRMS (ESI^+^) *m*/*z*: 1031.0260, calculated for C_44_H_30_Br_2_IrN_4_O_4_^+^: 1031.0243. Elemental analysis PF_6_: experimental (C, 44.95; H, 2.59; N, 4.74), calculated (C, 44.95; H, 2.57; N, 4.77).

Complex Ir1-F (yield 62%): red crystalline material. Crystals of Ir1-F are prism, C_44_H_30_F_2_IrN_4_O_4_∙PF_6_, *P* –1, a = 11.4642(6), b = 13.0721(6), c = 19.0187(6) Å, α = 93.694(3), β = 102.128(4), γ = 115.584(5)°, V = 2474.1(2) Å^3^, Z = 2, R_1_ = 0.0331, CCDC 2242687. ^1^H NMR (400 MHz, acetone-*d_6_*), ppm: dark red powder, 8.95 (s, 1H, H_9_), 8.47 (d, 1H, ^3^J = 7.55, H_10_), 8.37–8.33 (m, 3H, H_6,7,1_), 8.17 (td, 1H, ^3^J = 7.51, H_11_), 7.74–7.67 (m, 2H, H_5,12_), 7.25 (t, 1H, ^3^J = 7.51, H_2_), 7.06 (td, 1H, ^3^J = 8.16, H_8_), 6.92 (t, 1H, ^3^J = 7.30, H_3_), 6.68 (dd, 1H, ^3^J = 7.94, H_4_), 4.15 (s, 3H, H_13_). HRMS (ESI^+^) *m*/*z*: 909.1918, calculated for C_44_H_30_F_2_IrN_4_O_4_^+^: 909.1859. Elemental analysis calculated for C_44_H_30_F_2_IrN_4_O_4_PF_6_: experimental (C, 50.18; H, 2.91; N, 5.35), calculated (C, 50.14; H, 2.87; N, 5.32).

Complex Ir2-F (yield 58%): dark red crystalline material. ^1^H NMR (400 MHz, acetone-*d_6_*), ppm: 8.96 (s, 1H, H_9_), 8.47 (d, 1H, ^3^J = 5.04, H_10_), 8.37–8.28 (m, 3H, H_1,2,8_), 8.19 (td, 1H, ^3^J = 7.33, H_11_), 7.75 (td, 1H, ^3^J = 5.72, H_12_), 7.67 (tt, 1H, ^3^J = 4.35, H_5_), 7.50 (dd, 1H, ^3^J = 8.47, H_3_), 7.10 (td, 1H, ^3^J = 8.47, H_7_), 6.86 (d, 1H, ^3^J = 2.06, H_4_), 4.14 (s, 3H, H_13_). HRMS (ESI^+^) *m*/*z*: 1067.01, calculated for C_44_H_28_Br_2_F_2_IrN_4_O_4_^+^: 1067.0080. Elemental analysis calculated for C_44_H_28_Br_2_F_2_IrN_4_O_4_PF_6_: experimental (C, 43.60; H, 2.33; N, 4.62), calculated (C, 43.61; H, 2.33; N, 4.62).

Complex Ir3-F (yield 63%): dark red crystalline material. Crystals of Ir3-F are prism, C_40_H_26_F_2_IrN_4_O_4_S_2_∙PF_6_∙CH_2_Cl_2_, *P* –1, a = 9.5712(2), b = 14.8269(3), c = 16.0282(2) Å, α = 99.846(2), β = 104.184(2), γ = 107.021(2)°, V = 2035.08(8) Å^3^, Z = 2, R_1_ = 0.0582, CCDC 2242686. ^1^H NMR (400 MHz, acetone-*d_6_*), ppm: 8.61 (d, 1H, ^3^J = 8.31, H_1_), 8.44 (s, 1H, H_7_), 8.34 (dd, 1H, ^3^J = 10.75, H_8_), 8.26–8.22 (m, 2H, ^3^J = 7.33, H_3,4_), 7.80 (td, 1H, ^3^J = 6.11, H_2_), 7.73 (d, 1H, ^3^J = 4.64, H_6_), 7.47 (dd, 1H, ^3^J = 9.96, H_10_), 7.08 (td, 1H, ^3^J = 8.26, H_9_), 6.50 (d, 1H, ^3^J = 5.34, H_5_), 4.13 (s, 3H, H_11_). HRMS (ESI^+^) *m*/*z*: 921.09, calculated for C_40_H_26_F_2_IrN_4_O_4_S_2_^+^: 921.10. Elemental analysis calculated for C_40_H_26_F_2_IrN_4_O_4_S_2_PF_6_: experimental (C, 45.10; H, 2.42; N, 5.28), calculated (C, 45.07; H, 2.46; N, 5.26).

Complex Ir4-F (yield 60%): brown crystalline material. Crystals of Ir4-F are prism, C_48_H_30_F_2_IrN_4_O_4_S_2_∙0.75(CH_2_Cl_2_), *P* –1, a = 9.8800(1), b = 12.6383(1), c = 20.6634(2) Å, α = 76.610(1), β = 85.090(1), γ = 79.244(1)°, V = 2463.50(4) Å^3^, Z = 2, R_1_ = 0.0483, CCDC 2242685. ^1^H NMR (400 MHz, acetone-*d_6_*), ppm: 8.58 (s, 1H, H_9_), 8.55 (d, 1H, ^3^J = 5.68, H_5_), 8.45 (d, 1H, ^3^J = 8.37, H_8_), 8.33 (dt, 1H, ^3^J = 11.07, H_10_), 8.21 (t, 1H, ^3^J = 7.62, H_6_), 8.03 (dd, 1H, ^3^J = 8.07, H_1_), 7.84 (t, 1H, ^3^J = 6.12, H_7_), 7.42–7.37 (m, 1H, H_12_), 7.25 (t, 1H, ^3^J = 7.92, H_2_), 6.92 (td, 1H, ^3^J = 8.37, H_11_), 6.75 (t, 1H, ^3^J = 7.67, H_3_), 6.53 (t, 1H, ^3^J = 5.72, H_4_), 4.17 (s, 1H, H_13_). HRMS (ESI^+^) *m*/*z*: 1021.1192, calculated for C_48_H_30_F_2_IrN_4_O_4_S_2_^+^: 1021.1300. Elemental analysis calculated for C_48_H_30_F_2_IrN_4_O_4_S_2_PF_6_: experimental (C, 49.41; H, 2.61; N, 4.83), calculated (C, 49.44; H, 2.59; N, 4.80).

Complex Ir1-NO_2_ (yield 48%): dark red crystalline material. ^1^H NMR (400 MHz, acetone-*d_6_*), ppm: 9.56 (d, 1H, ^4^J = 2.47, H_10_), 9.11 (s, 1H, H_9_), 8.48 (d, 2H, ^3^J = 7.72, H_1,8_), 8.42 (d, 1H, ^3^J = 5.02, H_5_), 8.19 (t, 1H, ^3^J = 7.72, H_6_), 7.93 (dd, 1H, ^3^J = 9.72, H_11_), 7.83 (d, 1H, ^3^J = 9.72, H_12_), 7.77 (t, 1H, ^3^J = 6.77, H_7_), 7.33 (t, 1H, ^3^J = 8.28, H_2_), 6.99 (t, 1H, ^3^J = 7.25, H_3_), 6.82 (d, 1H, ^3^J = 8.20, H_4_), 4.19 (s, 3H, H_13_). HRMS (ESI^+^) *m*/*z*: 963.1774, calculated for C_44_H_30_IrN_6_O_8_^+^: 963.1749. Elemental analysis calculated for C_44_H_30_IrN_6_O_8_PF_6_: experimental (C, 47.71; H, 2.73; N, 7.57), calculated (C, 47.70; H, 2.73; N, 7.59).

Complex Ir2-NO_2_ (yield 51%): dark red crystalline material. Crystals of Ir2-NO_2_ are prism, C_44_H_28_Br_2_IrN_6_O_8_∙PF_6_∙0.75(C_3_H_6_O), *P* –1, a = 11.61180(10), b = 12.59440(10), c = 17.52830(10) Å, α = 73.9430(10), β = 89.5730(10), γ = 86.1790(10)°, V = 2457.76(3) Å^3^, Z = 2, R_1_ = 0.0328, CCDC 2242688. ^1^H NMR (400 MHz, acetone-*d_6_*), ppm: 9.55 (d, 1H, ^4^J = 2.58, H_9_), 9.11 (s, 1H, H_8_), 8.52 (d, 1H, ^3^J = 5.45, H_5_), 8.46 (d, 1H, ^3^J = 8.57, H_2_), 8.44 (d, 1H, ^3^J = 7.59, H_6_), 8.19 (td, 1H, ^3^J = 7.96, H_1_), 7.96 (dd, 1H, ^3^J = 9.56, H_11_), 7.81 (d, 1H, ^3^J = 9.56, H_7_), 7.80 (td, 1H, ^3^J = 7.11, H_10_), 7.57 (d, 1H, ^3^J = 8.46, H_3_), 7.01 (d, 1H, ^3^J = 1.86, H_4_), 4.17 (s, 3H, H_12_). HRMS (ESI^+^) *m*/*z*: 1121.0097, calculated for C_44_H_28_Br_2_IrN_6_O_8_^+^: 1120.9959. Elemental analysis calculated for C_44_H_28_Br_2_IrN_6_O_8_PF_6_: experimental (C, 41.78; H, 2.20; N, 6.69), calculated (C, 41.75; H, 2.23; N, 6.64).

Complex Ir4-NO_2_ (yield 67%): dark green crystalline material. ^1^H NMR (400 MHz, acetone-*d_6_*), ppm: 9.53 (d, 1H, ^4^J = 2.54, H_10_), 8.71 (s, 1H, H_9_), 8.61 (dd, 1H, ^3^J = 5.57, H_5_), 8.43 (d, 1H, ^3^J = 8.11, H_8_), 8.24 (td, 1H, ^3^J = 7.74, H_6_), 8.08 (d, 1H, ^3^J = 8.11, H_1_), 7.90 (td, 1H, ^3^J = 5.77, H_7_), 7.77 (dd, 1H, ^3^J = 9.71, H_11_), 7.58 (d, 1H, ^3^J = 9.58, H_12_), 7.33 (td, 1H, ^3^J = 7.37, H_2_), 6.79 (t, 1H, ^3^J = 7.65, H_3_), 6.64 (d, 1H, ^3^J = 7.91, H_3_), 4.20 (s, 3H, H_12_). HRMS (ESI^+^) *m*/*z*: 1075.1361, calculated for C_48_H_30_IrN_6_O_8_S_2_^+^: 1075.1190. Elemental analysis calculated for C_48_H_30_IrN_6_O_8_S_2_PF_6_: experimental (C, 47.25; H, 2.50; N, 6.89), calculated (C, 47.25; H, 2.48; N, 6.89).

### 3.3. X-ray Structure Determinations

The crystals of Ir1-H, Ir1-F, Ir2-NO_2_, Ir3-F, and Ir4-F were immersed in cryo-oil, mounted in a Nylon loop, and measured at a temperature of 100 K. The diffraction data were collected with Agilent Technologies “Xcalibur” diffractometers (Oxford, UK) using Mo Kα radiation (λ = 0.71073 Å) and Cu Kα (λ = 1.54184 Å) radiation, respectively. Diffraction data were processed in the CrysAlisPro program (Agilent Technologies; Versions 1.171.36.32, 2013 (Ir1-F), 1.171.40.67a, 2019 (Ir1-H), 1.171.40.69a, 2020 (Ir2-NO_2_), and 1.171.40.71a, 2020 (Ir3-F, Ir4-F)). The structures were solved by the dual-space algorithm and refined using the SHELX programs [36,37] incorporated in the OLEX2 program package [38]. The unit cells of Ir1-F and Ir2-NO_2_ contained disordered solvent molecules, and Ir4-F contained disordered counterion PF_6_^−^, respectively, which were treated as a diffuse contribution to the overall scattering without specific atom positions by SQUEEZE/PLATON [39]. Supplementary crystallographic data for this paper have been deposited at the Cambridge Crystallographic Data Centre and can be obtained free of charge via www.ccdc.cam.ac.uk/structures/ (accessed on 16 February 2023).

### 3.4. Photophysical Experiments

The photophysical characteristics of complexes were investigated in freshly distilled dichloromethane solution. Absorption UV-vis spectra were measured using a Shimadzu UV-1800 spectrometer (Shimadzu, Kyoto, Japan). Excitation spectra were recorded using a Fluorolog-3 (JY Horiba Inc., Kyoto, Japan) spectrofluorometer. Emission spectra were measured using an Avantes AvaSpec-2048x64 spectrometer with LED 365 nm pumping. Quantum yields were calculated using a comparative method with [Ru(bpy)_3_](PF_6_)_2_ in water (*Φ_r_* = 0.042) as a standard [40]. The reference refraction indexes were: 1.33 (water), 1.42 (dichloromethane). The equation to calculate quantum yields [41]:

(1) $$\Phi_{S}=\Phi_{r}\frac{\eta_{S}^{2}\cdot A_{r}\cdot I_{S}}{\eta_{r}^{2}\cdot A_{S}\cdot I_{r}}$$

where *Φ_S_*—the quantum yield of the sample, *Φ_r_*—the quantum yield of the reference, *η*—the refractive index of the solvent, *A_s_, A_r_*—the absorbance of the sample and the reference at the wavelength of excitation of emission, respectively, and *I_s_*, *I_r_*—the integrated area of emission band of the sample and the reference, respectively.

### 3.5. Calculation Details

All calculations were performed within the density functional theory (DFT) [42,43] framework applying the Gaussian-16 computer code [44]. The geometries of the ground singlet and first excited triplet states were optimized for all complexes under consideration. The frequency calculation was also carried out for all structures. Negative values of vibrational frequencies were not detected. The hybrid functional B3LYP [45] was used for all calculations. The Stuttgart–Dresden effective core pseudopotential and the corresponding basis set were chosen for iridium [46]; for carbon and hydrogen atoms, we used Pople’s 6-31G* Gaussian-type function basis set, and all other atoms were calculated within 6-311+G* [47]. The polarizable continuum model (PCM) [48] was used for taking into account the non-specific solvation effects.

The electronic absorption spectra were obtained for all compounds with time-dependent expansion of DFT (TD-DFT). After calculation, the UV/Vis spectra were converted from oscillator strengths to experimental extinction coefficients [49] to provide the ability to compare data. The energies of phosphorescence maxima were obtained as the difference between the energy of the optimized triplet and ground singlet states.

The most intensive absorption and emission transitions were analyzed using two methods. First of all, the natural transition orbitals (NTOs) [50] were plotted, which allowed visualization of the redistribution of electronic density during transitions. A quantitative ratio of charges transferred between the corresponding parts of the molecules was obtained by calculation of the interfragment charge transfer (IFCT) [51]. Both methods were realized in the Multiwfn 3.6 program [51]. The changes in electronic density Δρ during the *S_0_ → S_i_* transitions were calculated as:

$$\Delta \rho \left(S_{0}\to S_{i}\right)=\underset{k}{\sum }{\left|\Psi_{ik}\left(virt\right)\right|}^{2}-\underset{k}{\sum }{\left|\Psi_{ik}\left(occ\right)\right|}^{2}$$

where *Ψ_ik_(occ)* and *Ψ_ik_(virt)* are NTO pairs for *S_0_→S_i_* transition. The electronic density’s change during T_1_→S_0_ transition was obtained in an analogous manner.

## 4. Conclusions

A series of the bis-metalated phosphorescent [(N^C)_2_Ir(bipyridine)]^+^ complexes with systematic variations in the structure and electronic characteristics of the N^C ligands were synthesized and characterized. Investigation of the complexes’ spectroscopic properties, together with DFT and TD DFT calculations, revealed that metal-to-ligand (MLCT) and intraligand (LC) charge transfer processes play a key role in the generation of both the lowest energy singlet (S_1_) and the emissive (T_1_) states in the studied emitters. It is worth noting that according to the results of theoretical studies, the LC character, which makes a major contribution in the generation of the above mentioned excited states, is more accurately considered as an intraligand charge transfer process between N- and C-coordinated moieties of the N^C chelate. This finding is completely in line with the trends observed in the experimental absorption and emission spectra, where the lowest absorption transitions and wavelengths of emission bands displayed systematic bathochromic shifts upon insertion of electron-withdrawing substituents into the N-coordinated fragment of the N^C ligand. An analogous shift was also induced by expansion of the aromatic system of the C-coordinated fragment and insertion of polarizable sulfur atoms into the structure of the aromatic rings. These experimental and theoretical findings extend our knowledge of the nature of photophysical processes in complexes of this type, which are widely used in numerous practical applications, and provide useful instruments for fine-tuning of their emissive characteristics.

## Figures and Tables

**Table 1:** Photophysical data for iridium complexes, 298 K, dichloromethane solution.

	Abs, nm (ε × 10^−4^, L × mol^−1^ × cm^−1^)	λ_ems_, nm ^a^	λ_exc_, nm ^b^	Φ, % ^b^ (aer/degas)	τ, µs ^c^ (aer/degas)	k_r_, s^−1^ (degas) ^d^	k_nr_, s^−1^ (degas) ^e^	λ_ems_, (calc)
Ir1-H	267 (54), 293 (49), 355 (26), 473 (4)	636, 668sh	273, 293, 363, 465	17/45	0.53/1.14	3.9 × 10^5^	4.8 × 10^5^	638
Ir1-F	268 (59), 295 (57), 357 (30), 474 (5)	641, 665sh	273, 293, 364, 468	16/28	0.53/0.87	3.2 × 10^5^	8.3 × 10^5^	654
Ir1-NO_2_	260 (44), 306 (42), 366 (29), 511 (5)	671	267, 309, 365, 499, 535sh	13/20	0.25/0.33	6.0 × 10^5^	2.4 × 10^6^	708
Ir2-H	267 (51), 297 (54), 348sh ^f^ (27), 365 (28), 460 (6)	614, 656sh	273, 293, 346sh, 364, 452	17/49	0.72/1.98	2.4 × 10^5^	2.6 × 10^5^	560
Ir2-F	270 (42), 300 (44), 345 (21), 365sh (20), 466 (5)	623, 663sh	273, 297, 348sh, 364, 458	18/55	0.71/2.10	2.6 × 10^5^	2.1 × 10^5^	632
Ir2-NO_2_	262 (29), 310 (27), 365 (21), 488 (4.5)	634, 690sh	269, 309, 365, 482, 516sh	47/81	0.63/1.07	7.6 × 10^5^	1.7 × 10^5^	657
Ir3-F	306 (30), 336 (20), 408sh (8), 498 (5)	692, 740sh	306, 349, 398sh, 495	8/21	0.29/0.68	3.1 × 10^5^	1.2 × 10^6^	642
Ir4-F	372 (41), 316 (49.5), 343 (32), 383sh (22.5), 438sh (10), 554 (7)	766, 820sh	290, 366, 528	<0.1/0.1	0.12/0.16	3.1 × 10^3^	6.2 × 10^6^	715
Ir4-NO_2_	270 (42), 315sh (35.5), 328 (36), 395 (31), 458sh (13), 590 (12)	792, 822sh	297, 335sh, 366, 579, 596	<0.1/0.1	0.07/0.09	1.1 × 10^4^	1.1 × 10^7^	779

^a^ λ_ex_ = 365 nm. **^b^** Emission quantum yields were determined in aerated (aer) and degassed (degas) dichloromethane solution at λ_ex_ = 365 nm. ^c^ Emission decays in aerated (aer) and degassed (degas) dichloromethane solution and excitation spectra were measured at the maximum of the emission bands; lifetime was obtained using mono-exponential fitting. ^d^ k_r_ = Φ/τ_obs_. ^e^ k_nr_ = k_obs_ − k_r_ = (1 − Φ)/τ_obs_. ^f^ sh—shoulder.

## Data Availability

Data are contained within the article or Supplementary Materials.

## Supplementary Materials

The following supporting information can be downloaded at: https://www.mdpi.com/article/10.3390/molecules28062740/s1, Scheme S1. Synthesis of the N^C proligands. Table S1. Key structural parameters of the [Ir(N^C)2(N^N)]PF6 complexes (schematic numbering of related atoms is represented in the figures in the right part of the table). Figures S1–S8. 1H NMR spectra of ligands. Figures S9–S24. 1H and 1H-1H COSY NMR spectra of complexes. Figures S25–S33. ESI+ mass spectra of complexes. Figure S34. Absorption spectra of all synthesized complexes in DCM: (a) Ir1-H, Ir1-F, Ir1-NO2; (b) Ir2-H, Ir2-F, Ir2-NO2; (c) Ir3-F, Ir4-F, Ir4-NO2. Figures S35–S43. Absorption spectra of complexes: experimental (red) and calculated (black) lines with oscillator strengths of electronic transitions (bars). Tables S2, S4, S6, S8, S10, S12, S14, S16 and S18. Experimental and calculated absorption maxima (λ), extinction coefficients (ε), oscillator strengths (f) of complexes. Tables S3, S5, S7, S9, S11, S13, S15, S17 and S19. The decrease (violet) and increase (terracota) in electron density for the most intense electronic absorption transitions of complexes. The data for the corresponding interfragment charge transfer (IFCT) are given below the figures. Diagonal values represent intraligand transitions, off-diagonal values represent a charge transfer from “Donor” to “Acceptor”.

## Sample Availability

Samples of the compounds are available from the authors on request.
