# Supplementary material for: Investigation of the N^C Ligand Effects on Emission Characteristics in a Series of Bis-Metalated [Ir(N^C)2(N^N)]+ Complexes
Source: Molecules. 2023 Mar 17;28(6):2740. doi: 10.3390/molecules28062740 (PMC10054739; doi:10.3390/molecules28062740)
Supplement: Supplementary file 1 [file molecules-28-02740-s001.zip › molecules-2271469-supplementary.pdf]

Supplementary Materials

for the article

Part 1. Synthesis of the metalating ligands.

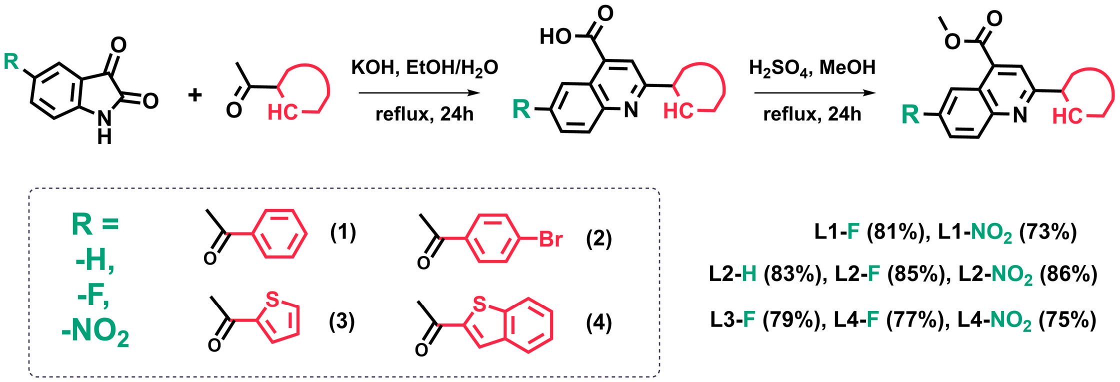

Scheme S1. Synthesis of the N^C proligands.

General procedure: In 100 mL RBF, the appropriate Isatin (0.01 mol), ketone (0.01 mol), and KOH (0.03 mol) were dissolved in ethanol (20 mL) and the reaction mixture was refluxed for 24-72 h. the progress of the reaction was monitored using TLC. After the reaction was terminated, the solvent evaporation afforded a residue, which was dissolved in water (20 mL). The solution was washed twice with diethyl ether (10 mL). the aqueous phase was acidified to pH 1 with 37% HCl and the precipitate collected by suction filtration, washed with water and dried.

In order to prepare the methylated proligand, 1 mL of conc. H_2SO_4 was added to a methanol (30 mL) solution of the compound prepared at the previous stage. The reaction mixture was refluxed overnight. Solvent was removed under reduced pressure. The residue was partitioned between EtOAc and saturated $NaHCO_3$. The organic layer was separated and washed with water and brine, dried over anhydrous Na_2SO_4 and evaporated to give the desired compound.

For the proton numbering schemes in the ^{1}H NMR spectra of the ligands see Figures S1-S8.

L1-F. 6-fluoro-2-phenylquinoline-4-carboxylic acid (yield 88%): Yellow powder, ^{1}H NMR (400 MHz, DMSO-*d6*), ppm: 8.69 (s, 1H, H_6), 8.63 (dd, 1H, ^3J = 10.71, H_7), 8.35 (d, 2H, ^3J = 8.36, $H_{1,5}$), 8.28 (td, 1H, ^3J = 6.79, H_8), 7.73 (td, 1H, ^3J = 8.88, H_9), 7.59 (m, 3H, $H_{2,3,4}$). Methyl 6-fluoro-2-phenylquinoline-4-carboxylate (yield 81%): Yellow powder, ^{1}H NMR (400 MHz, DMSO-*d6*), ppm: 8.57 (s, 1H, H_6), 8.36 (dd, 1H, ^3J = 8.35, H_7), 8.29 (m, 3H, $H_{1,5,8}$), 7.82 (td, 1H, ^3J = 8.68, H_9), 7.58 (m, 3H, $H_{2,3,4}$), 4.04 (s, 3H, H_{10}).

L1-NO₂. 6-nitro-2-phenylquinoline-4-carboxylic acid (yield 84%): Yellow powder, ^{1}H NMR (400 MHz, DMSO-*d6*), ppm: 14.38 (s, 1H, OH), 9.70 (d, 1H, ^4J = 2.64, H_7), 8.67 (s, 1H, H_6), 8.56 (dd, 1H, ^3J = 9.23, H_8), 8.37-8.33 (m, 3H, $H_{1,2,9}$), 7.65-7.60 (m, 3H, $H_{3,4,5}$). Methyl 6-nitro-2-

phenylquinoline-4-carboxylate (yield 73%): Yellow powder, ^{1}H NMR (400 MHz, DMSO-*d6*), ppm: 9.56 (d, 1H, $^4J = 2.25$, H_7), 8.67 (s, 1H, H_6), 8.55 (dd, 1H, $^3J = 9.67$, H_8), 8.36-8.33 (m, 3H, $H_{1,2,9}$), 7.63-7.61 (m, 3H, $H_{3,4,5}$), 4.19 (s, 3H, H_{10}).

L2-H. 2-(4-bromophenyl) quinoline-4-carboxylic acid (yield 85%). White off powder, ^{1}H NMR (400 MHz, DMSO-*d6*), ppm: 14.03 (s, 1H, OH), 8.66 (d, 1H, $^3J = 8.33$, H_6), 8.48 (s, 1H, H_5), 8.28 (d, 2H, $^3J = 8.65$, $H_{1,2}$), 8.18 (d, 1H, $^3J = 8.46$, H_9), 7.88 (t, 1H, $^3J = 7.61$, H_7), 7.78 (d, 2H, $^3J = 8.76$, $H_{3,4}$), 7.73 (t, 1H, $^3J = 7.8$, H_8). Methyl 2-(4-bromophenyl) quinoline-4-carboxylate (yield 83%). Off white powder, ^{1}H NMR (400 MHz, DMSO-*d6*), ppm: 8.56 (d, 1H, $^3J = 8.55$, H_6), 8.49 (s, 1H, H_5), 8.26 (d, 2H, $^3J = 8.09$, $H_{1,2}$), 8.18 (d, 1H, $^3J = 8.55$, H_9), 7.89 (t, 1H, $^3J = 7.63$, H_7), 7.78 (d, 2H, $^3J = 8.70$, $H_{3,4}$), 7.74 (t, 1H, $^3J = 7.63$, H_8), 4.04 (s, 3H, H_{10}).

L2-F. 2-(4-bromophenyl)-6-fluoroquinoline-4-carboxylic acid (yield 79%): Red powder, ^{1}H NMR (400 MHz, DMSO-*d6*), ppm: 8.57 (s, 1H, H_5), 8.47 (dd, 1H, $^3J = 11.34$, H_6), 8.26 (d_{broad}, 3H, $^3J = 8.76$, $H_{7,1,2}$), 7.84 (m, 3H, $H_{8,3,4}$). Methyl 2-(4-bromophenyl)-6-fluoroquinoline-4-carboxylate (yield 85%): Purple powder, ^{1}H NMR (400 MHz, DMSO-*d6*), ppm: 8.50 (s, 1H, H_5), 8.32 (dd, 1H, $^3J = 10.62$, H_6), 8.22-8.19 (m, 3H, $H_{7,1,2}$), 7.81-7.74 (m, 3H, $H_{8,3,4}$), 4.03 (s, 3H, H_9).

L2-NO$_2$. 2-(4-bromophenyl)-6-nitroquinoline-4-carboxylic acid (yield 91%): Yellow powder, ^{1}H NMR (400 MHz, DMSO-*d6*), ppm: 9.69 (d, 1H, $^4J = 2.65$, H_6), 8.68 (s, 1H, H_5), 8.56 (dd, 1H, $^3J = 9.39$, H_7), 8.40 (d, 1H, $^3J = 9.27$, H_8), 8.32 (d, 2H, $^3J = 8.65$, $H_{1,2}$), 7.82 (d, 2H, $^3J = 8.65$, $H_{3,4}$). Methyl 2-(4-bromophenyl)-6-nitroquinoline-4-carboxylate (yield 86%): Yellow powder, ^{1}H NMR (400 MHz, DMSO-*d6*), ppm: 9.60 (d, 1H, $^4J = 2.48$, H_6), 8.73 (s, 1H, H_5), 8.60 (dd, 1H, $^3J = 9.36$, H_7), 8.56 (d, 1H, $^3J = 9.36$, H_8), 8.35 (d, 2H, $^3J = 8.56$, $H_{1,2}$), 7.85 (d, 2H, $^3J = 8.56$, $H_{3,4}$), 4.08 (s, 3H, H_9).

L3-F. 6-fluoro-2-(thiophen-2-yl) quinoline-4-carboxylic acid (yield 83%): Dark red powder, ^{1}H NMR (400 MHz, Acetone-*d6*), ppm: 8.61 (s, 1H, H_4), 8.54 (dd, $^3J = 10.98$, 1H, H_5), 8.24 (dd, 1H, $^3J = 9.51$, H_6), 8.03 (dd, 1H, $^3J = 3.87$, H_3), 7.80 (dd, 1H, $^3J = 5.12$, H_1), 7.77 (dd, 1H, $^3J = 8.79$, H_7), 7.34 (dd, 1H, $^3J = 5.27$, H_2). Methyl 6-fluoro-2-(thiophen-2-yl) quinoline-4-carboxylate (yield 79%): Dark red powder, ^{1}H NMR (400 MHz, Acetone-d$_6$), ppm: 8.53 (s, 1H, H_4), 8.46 (dd, $^3J = 11.12$, 1H, H_5), 8.16 (dd, 1H, $^3J = 9.48$, H_6), 8.02 (dd, 1H, $^3J = 3.87$, H_3), 7.72 (dd, 1H, $^3J = 5.11$, H_1), 7.69 (dd, 1H, $^3J = 8.81$, H_7), 7.26 (dd, 1H, $^3J = 5.27$, H_2), 4.09 (s, 1H, H_8).

L4-F. 2-(benzo[b]thiophen-2-yl)-6-fluoroquinoline-4-carboxylic acid (yield 84%): Red powder, ^{1}H NMR (400 MHz, DMSO-*d6*), ppm: 8.70 (s, 1H, H_6), 8.51 (s, 1H, H_1), 8.46 (dd, 1H, $^3J = 11.34$, H_7), 8.18 (dd, 1H, $^3J = 9.45$, H_9), 8.03 (dd, 1H, $^3J = 4.16$, H_5), 7.94 (dd, 1H, $^3J = 4.16$, H_2), 7.79 (td, 1H, $^3J = 8.31$, H_8), 7.49-7.42 (m, 2H, $H_{3,4}$). Methyl 2-(benzo[b]thiophen-2-yl)-6-fluoroquinoline-4-carboxylate (yield 77%): Red powder, ^{1}H NMR (400 MHz, DMSO-*d6*), ppm: 8.71 (s, 1H, H_6), 8.52 (s, 1H, H_1), 8.36 (dd, 1H, $^3J = 10.96$, H_7), 8.20 (dd, 1H, $^3J = 9.59$, H_9), 8.04 (dd, 1H, $^3J = 4.26$, H_5), 7.96 (dd, 1H, $^3J = 4.26$, H_2), 7.82 (td, 1H, $^3J = 8.67$, H_8), 7.48-7.43 (m, 2H, $H_{3,4}$), 4.06 (s, 1H, H_{10}).

L4-NO₂. 2-(benzo[b]thiophen-2-yl)-6-nitroquinoline-4-carboxylic acid (yield 88%): Brown powder, ^{1}H NMR (400 MHz, DMSO-d_6), ppm: 9.68 (d, 1H, ^4J = 2.57, H_7), 8.81 (s, 1H, H_6), 8.66 (s, 1H, H_1), 8.55 (dd, 1H, ^3J = 9.35, H_8), 8.29 (dd, 1H, ^3J = 9.35, H_9), 8.07 (d, 1H, ^3J = 7.62, H_5), 7.99 (d, 1H, ^3J = 7.62, H_2), 7.51-7.44 (m, 2H, $H_{3,4}$). Methyl 2-(benzo[b]thiophen-2-yl)-6-nitroquinoline-4-carboxylate (yield 75%): Yellow powder, ^{1}H NMR (400 MHz, DMSO-d_6), ppm: 9.58 (d, 1H, ^4J = 2.56, H_7), 8.84 (s, 1H, H_6), 8.68 (s, 1H, H_1), 8.57 (dd, 1H, ^3J = 9.40, H_8), 8.31 (dd, 1H, ^3J = 9.40, H_9), 8.09 (d, 1H, ^3J = 7.74, H_5), 8.01 (d, 1H, ^3J = 7.74, H_2), 7.52-7.45 (m, 2H, $H_{3,4}$), 4.10 (s, 3H, H_{10}).

Part 2. Structural and spectroscopic data for ligands and complexes.

Table S1. Key structural parameters of the $[Ir(N^\wedge C)_2(N^\wedge N)]PF_6$ complexes (schematic numbering of related atoms is represented on the figures in the right part of the table).

	**Ir1-H**	**Ir1-F**	**Ir2-NO₂**	**Ir3-F**	**Ir4-F**
Bond lengths, Å					
Ir-N1	2.164	2.193	2.156	2.140	2.163
Ir-N2	2.168	2.169	2.174	2.150	2.146
Ir-N3	2.087	2.082	2.097	2.128	2.104
Ir-N4	2.077	2.092	2.085	2.128	2.093
Ir-C1	2.000	1.994	2.008	2.007	2.020
Ir-C2	2.015	1.995	2.007	1.990	2.013
Bond angles, °					
N1-Ir-N3	83.30	81.35	83.09	83.88	79.84
N1-Ir-N4	100.69	104.87	103.24	107.53	101.99
N2-Ir-N3	105.38	103.24	105.35	106.55	100.11
N2-Ir-N4	80.21	83.38	82.12	86.92	82.37
C1-Ir-N3	80.27	79.75	79.58	79.66	79.70
C1-Ir-N4	94.57	94.48	93.61	88.85	97.94
C2-Ir-N3	96.86	94.76	94.98	90.39	98.87
C2-Ir-N4	79.74	79.84	79.77	80.02	79.60
C–H···N nonbonding contacts, Å					
C3-N1	3.196	3.110	3.115	3.139	3.056
C4-N2	2.947	3.137	3.077	3.139	3.020
H3-N1	2.406	2.343	2.343	2.262	2.395
H4-N2	2.357	2.398	2.354	2.229	2.346

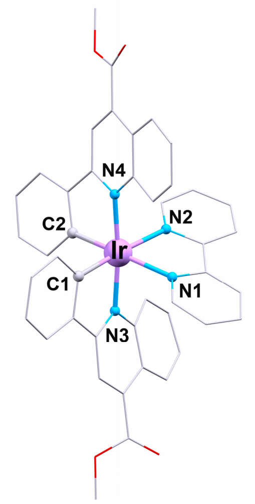

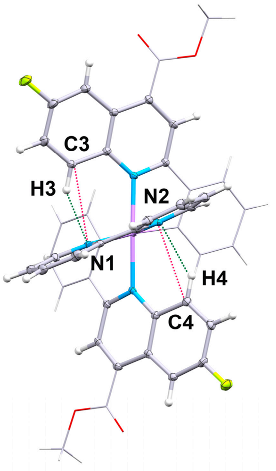

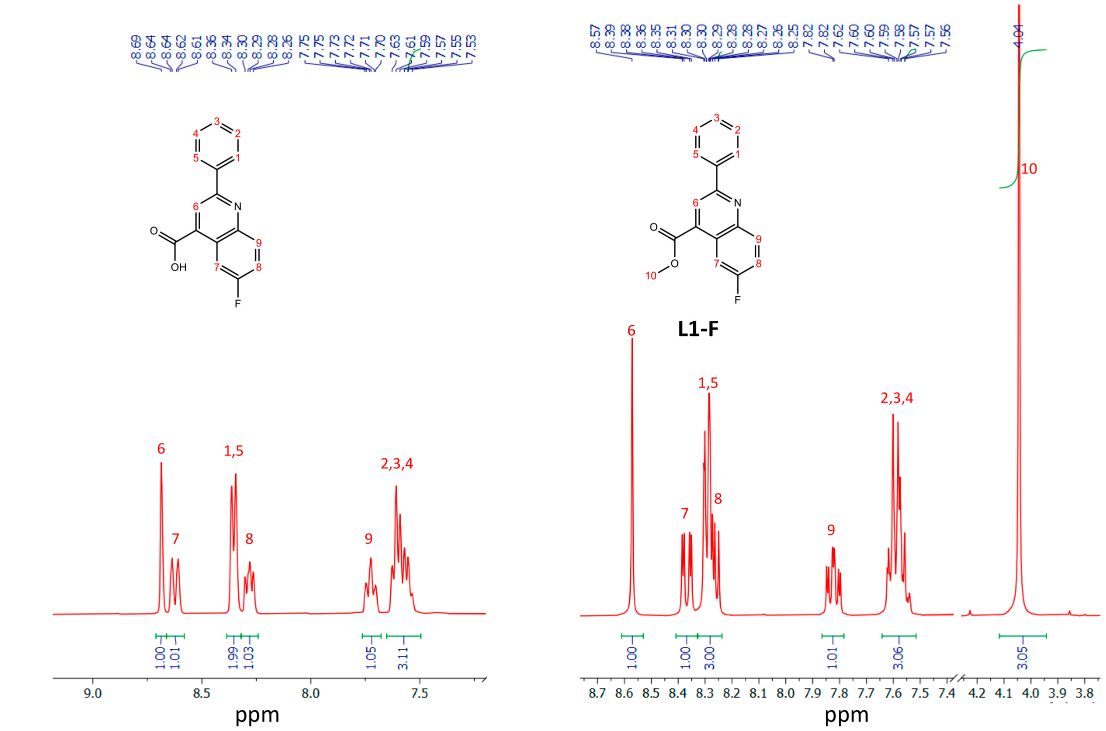

Figure S1. ^{1}H NMR spectrum of 6-fluoro-2-phenylquinoline-4-carboxylic acid and methyl 6-fluoro-2-phenylquinoline-4-carboxylate (**L1-F**) in DMSO-d_6 at 298 K.

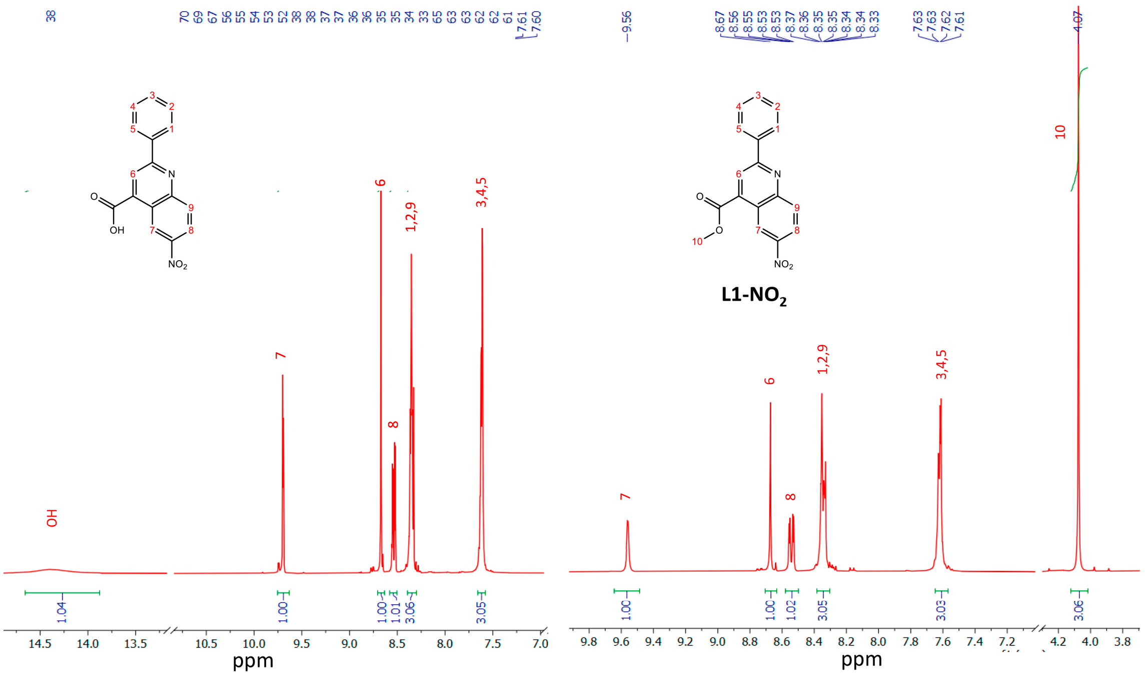

Figure S2. ^{1}H NMR spectrum of 6-nitro-2-phenylquinoline-4-carboxylic acid and ^{1}H NMR spectrum of methyl 6-nitro-2-phenylquinoline-4-carboxylate (**L1-NO$_2$**) in DMSO-d_6 at 298 K.

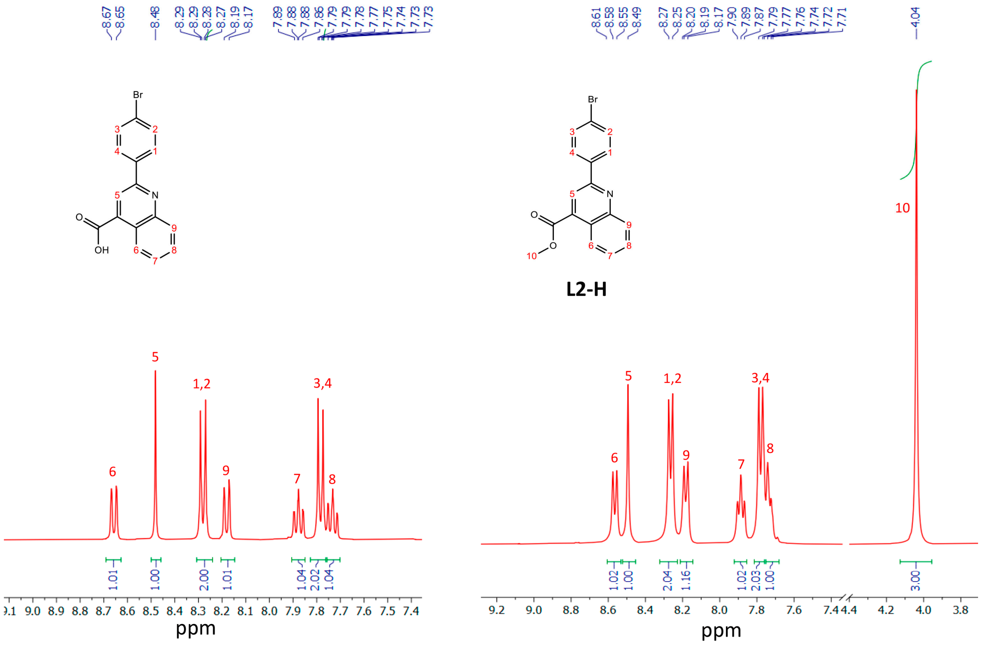

Figure S3. ^{1}H NMR spectrum of 2-(4-bromophenyl) quinoline-4-carboxylic acid and methyl 2-(4-bromophenyl) quinoline-4-carboxylate (**L2-H**) in DMSO-d_6 at 298 K.

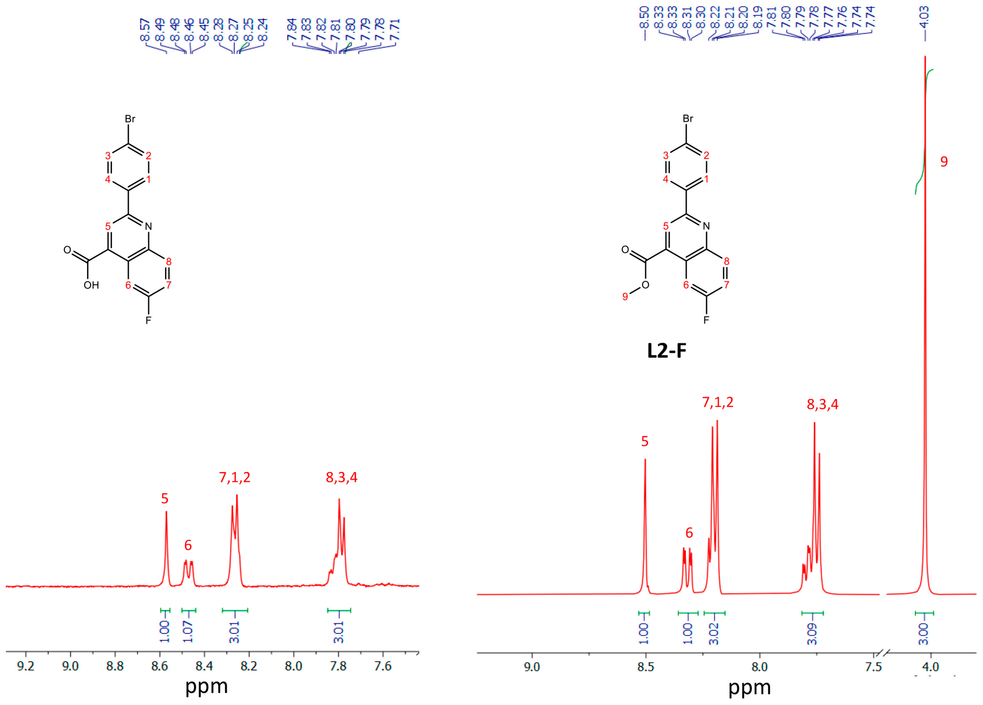

Figure S4. ^{1}H NMR spectrum of 2-(4-bromophenyl)-6-fluoroquinoline-4-carboxylic acid and methyl 2-(4-bromophenyl)-6-fluoroquinoline-4-carboxylate (**L2-F**) in DMSO-d_6 at 298 K.

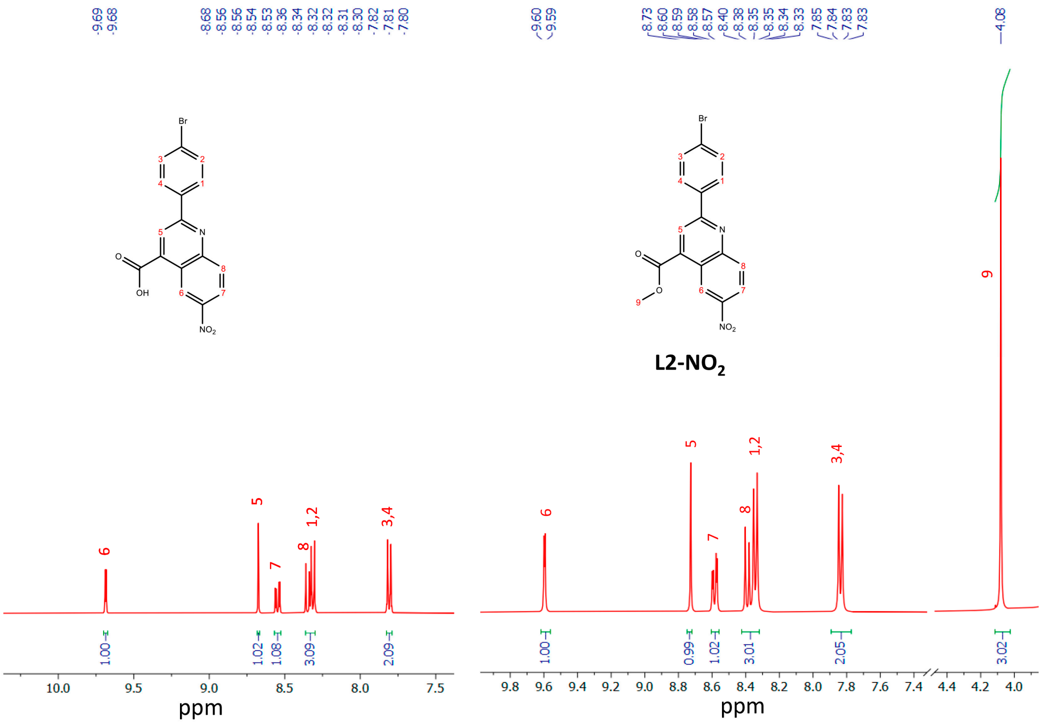

Figure S5. ^{1}H NMR spectrum of 2-(4-bromophenyl)-6-nitroquinoline-4-carboxylic acid and methyl 2-(4-bromophenyl)-6-nitroquinoline-4-carboxylate (**L2-NO$_2$**) in DMSO-d_6 at 298 K.

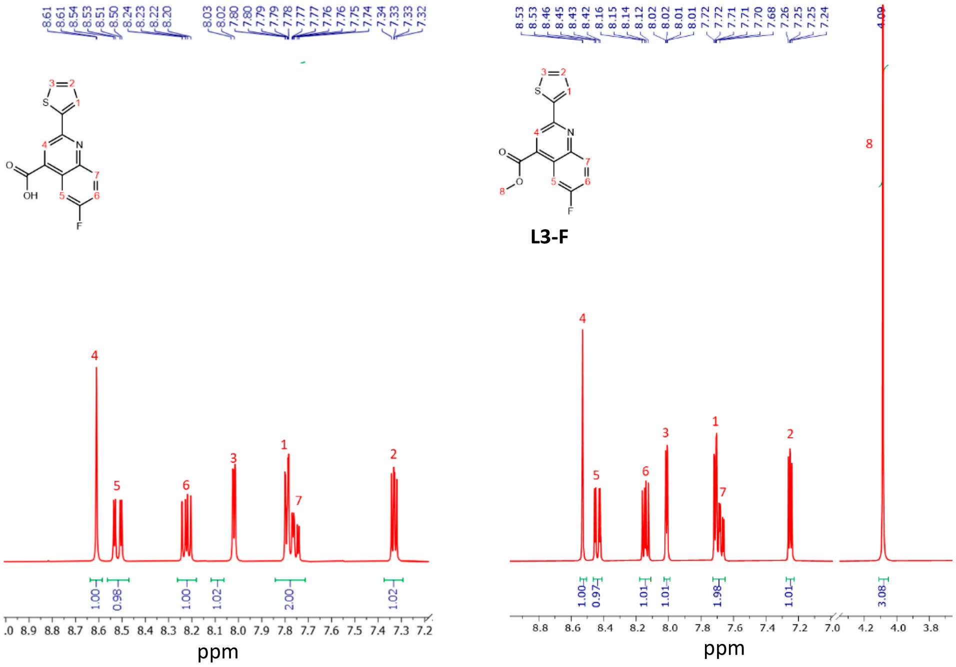

Figure S6. ^{1}H NMR spectrum of 6-fluoro-2-(thiophen-2-yl) quinoline-4-carboxylic acid and methyl 6-fluoro-2-(thiophen-2-yl) quinoline-4-carboxylate (**L3-F**) in Acetone-d_6 at 298 K.

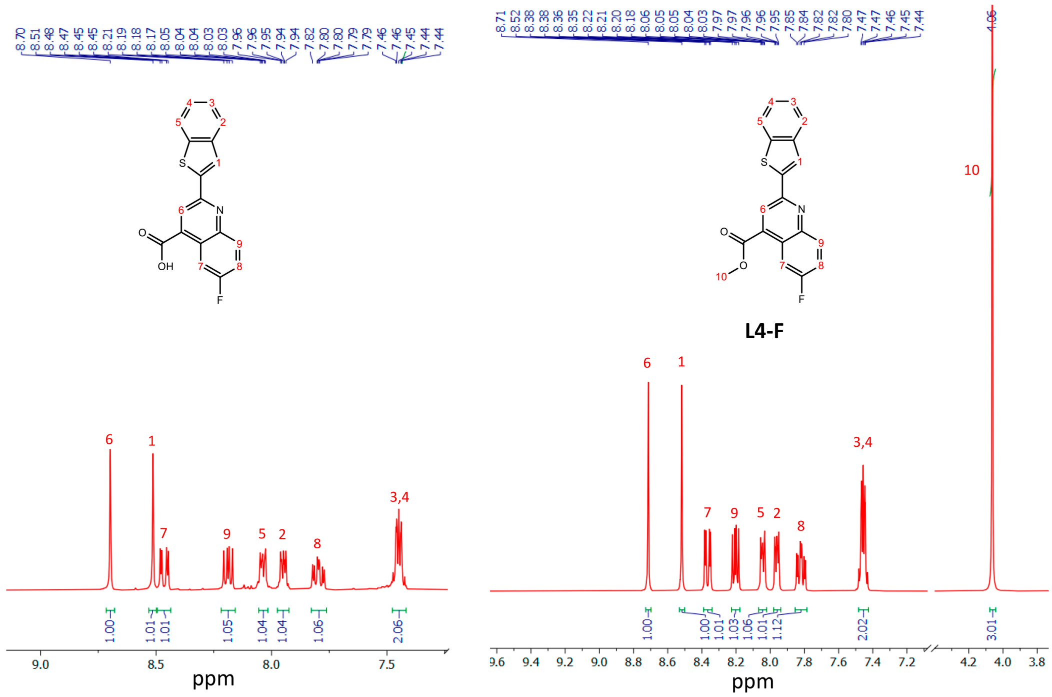

Figure S7. ^{1}H NMR spectrum of 2-(benzo[b]thiophen-2-yl)-6-fluoroquinoline-4-carboxylic acid and methyl 2-(benzo[b]thiophen-2-yl)-6-nitroquinoline-4-carboxylate (**L4-F**) in DMSO-*d6* at 298 K.

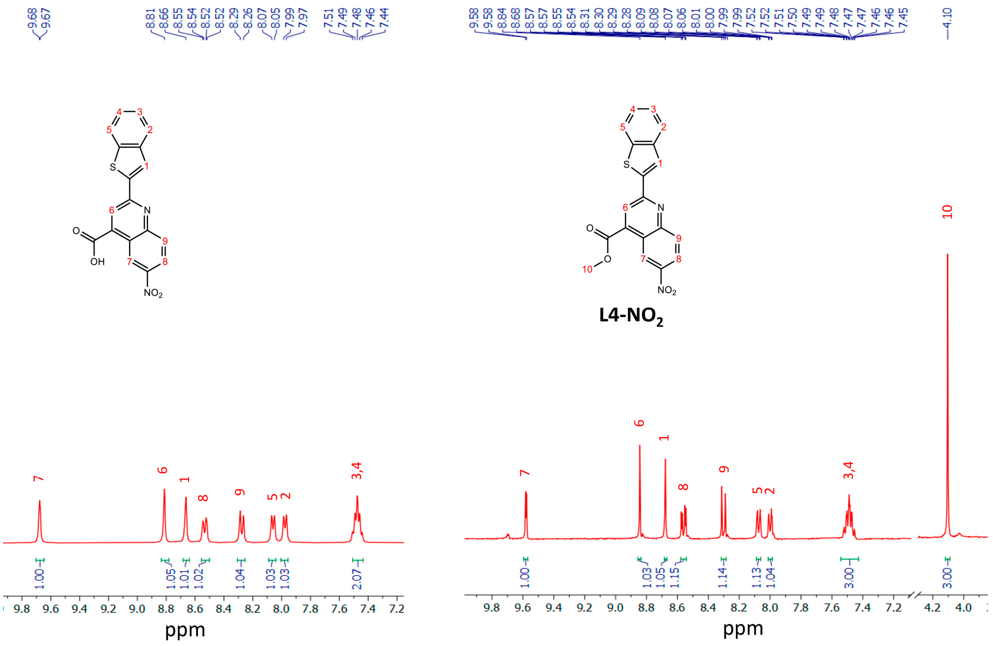

Figure S8. ^{1}H NMR spectrum of 2-(benzo[b]thiophen-2-yl)-6-nitroquinoline-4-carboxylic acid and methyl 2-(benzo[b]thiophen-2-yl)-6-nitroquinoline-4-carboxylate (**L4-NO$_2$**) in DMSO-*d6* at 298 K.

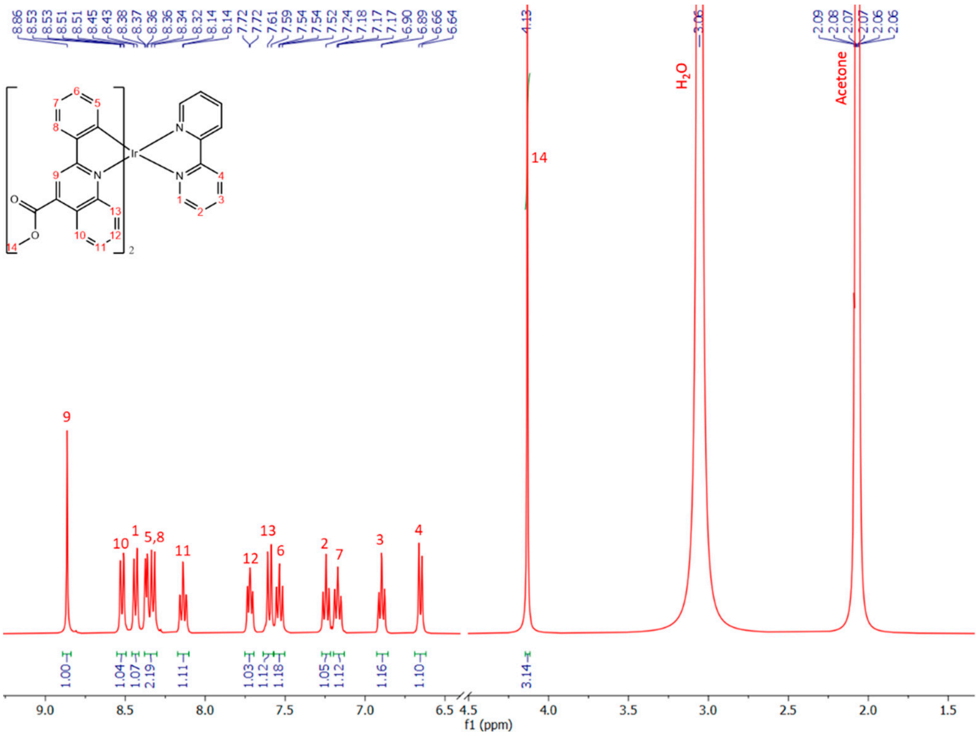

Figure S9. ^{1}H NMR spectrum of complex Ir1-H in Acetone-d_6 at 298 K.

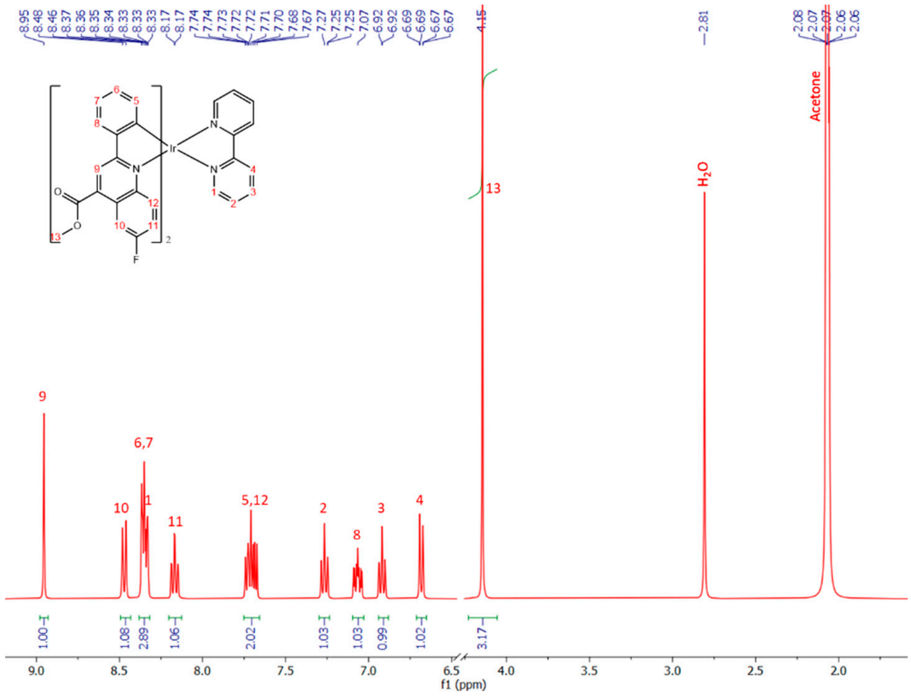

Figure S10. ^{1}H NMR spectrum of complex Ir1-F in Acetone-d_6 at 298 K.

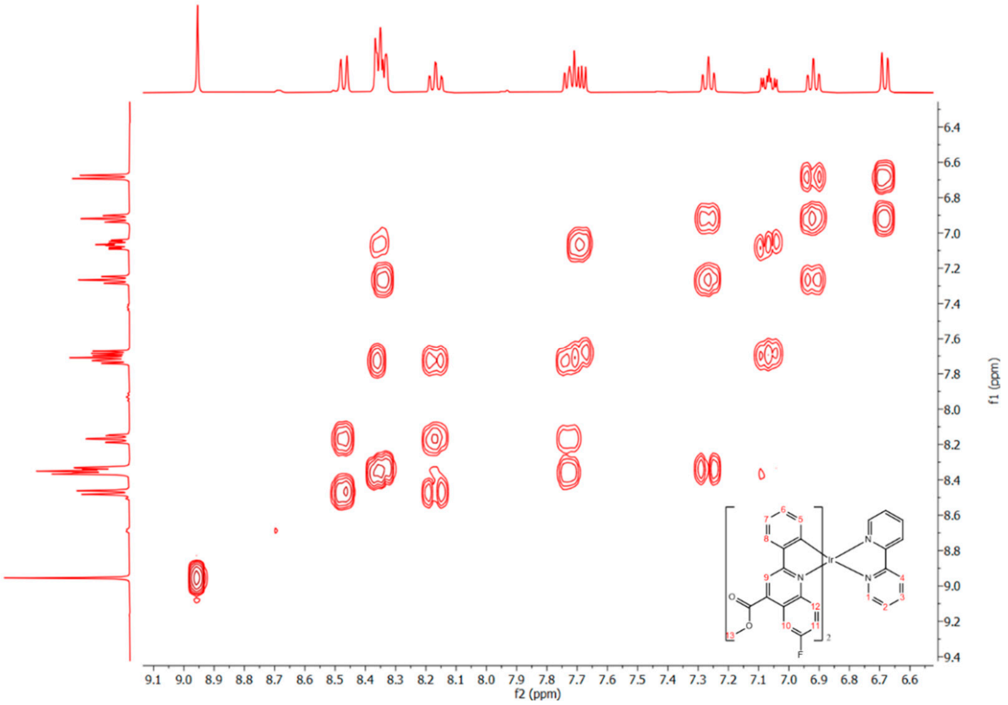

Figure S11. ^1H-^1H COSY NMR spectrum of Complex Ir1-F in Acetone-d_6 at 298 K.

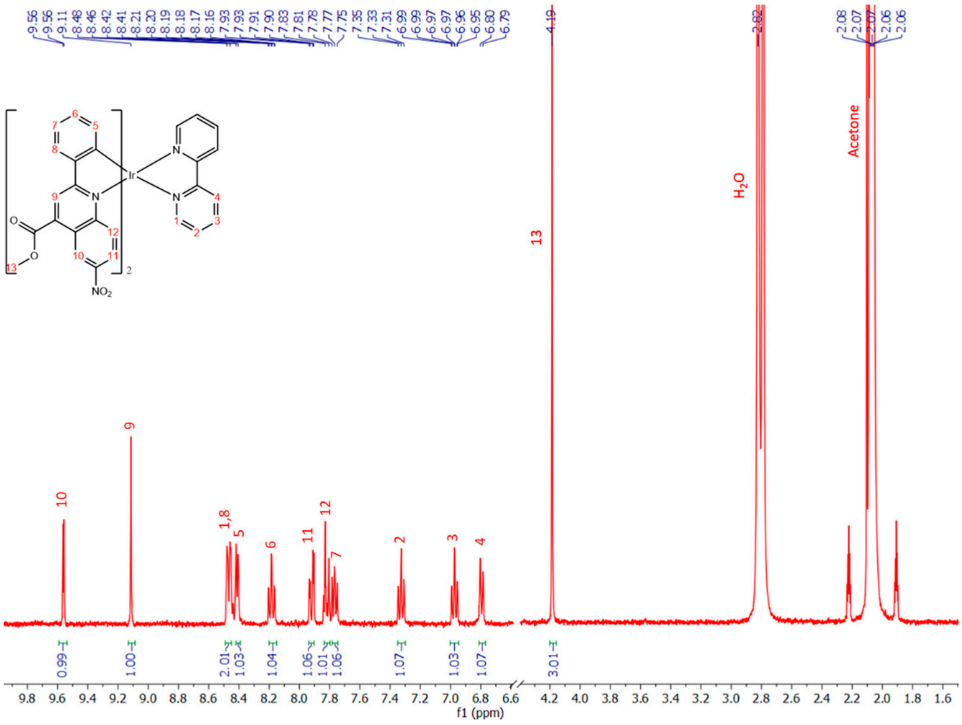

Figure S12. ^1H NMR spectrum of Complex Ir1-NO_2 in Acetone-d_6 at 298 K.

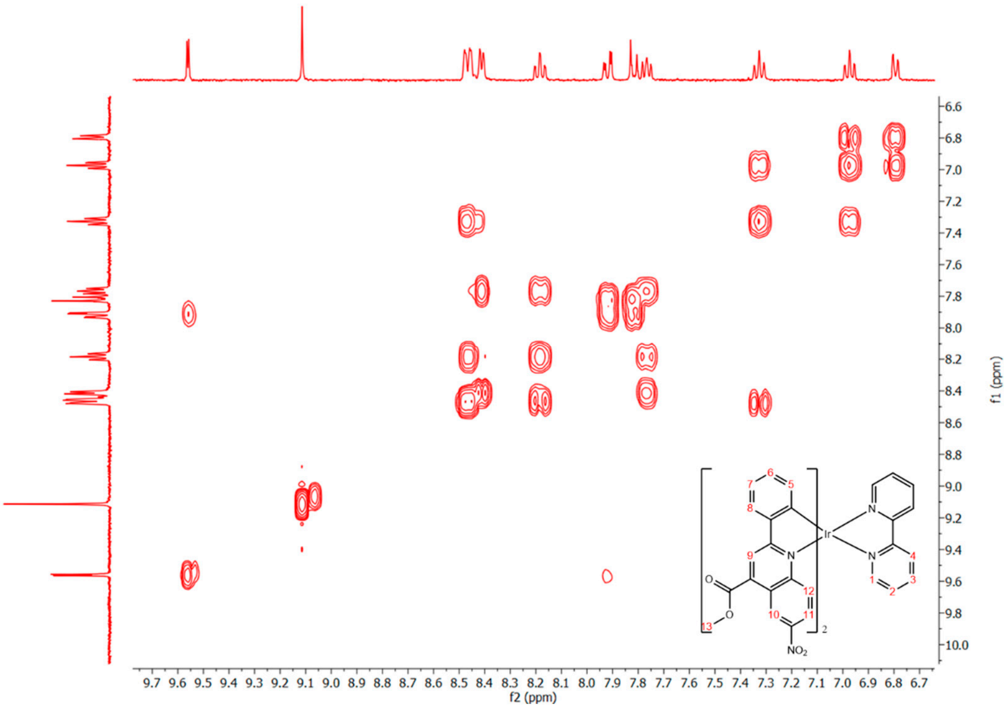

Figure S13. ^{1}H-^{1}H COSY NMR spectrum of Complex Ir1-NO_2 in Acetone-d_6 at 298 K.

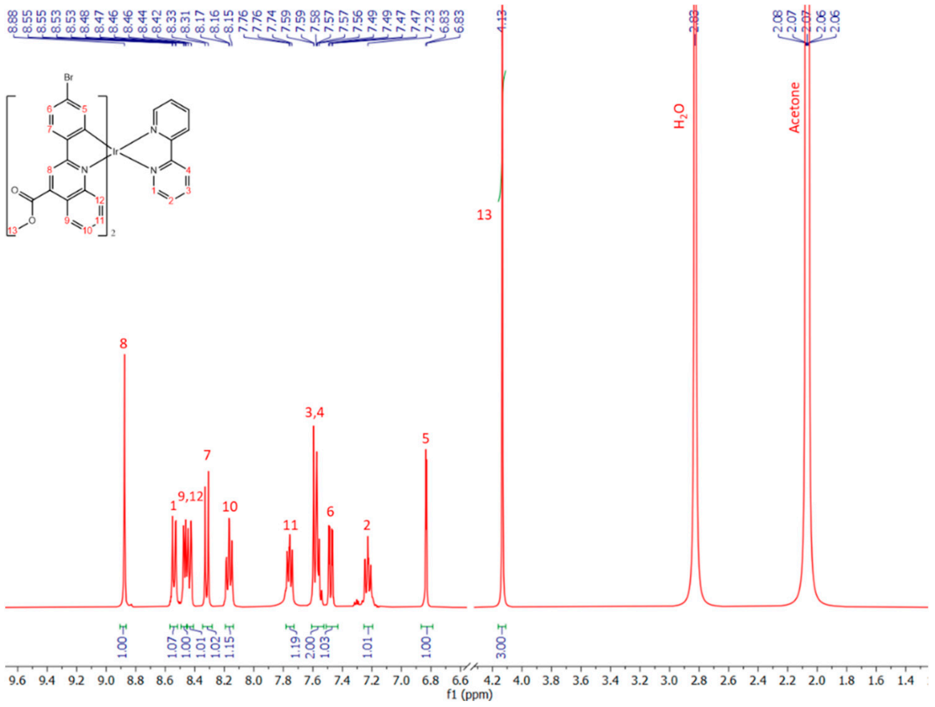

Figure S14. ^{1}H NMR spectrum of complex Ir2-H in Acetone-d_6 at 298 K.

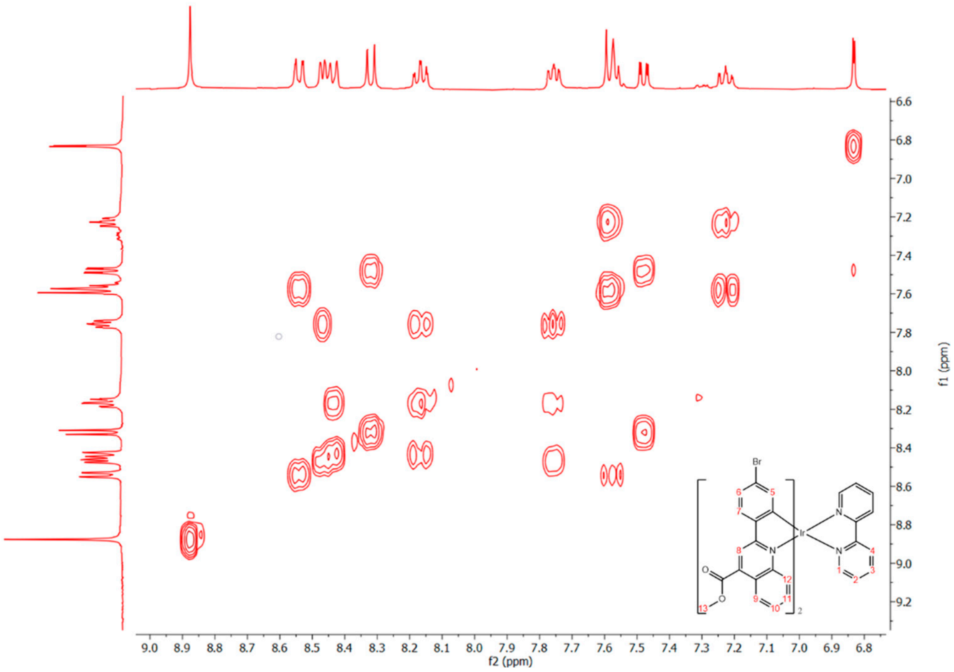

Figure S15. ^1H-^1H COSY NMR spectrum of Complex Ir2-H in DMSO-d_6 at 298 K.

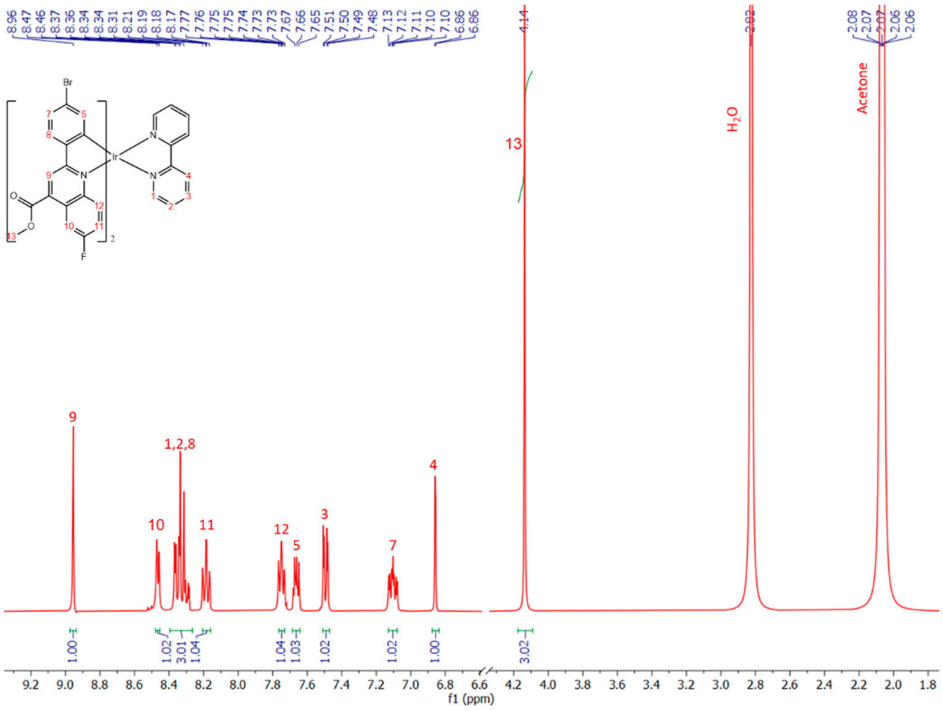

Figure S16. ^1H NMR spectrum of complex Ir2-F in Acetone-d_6 at 298 K.

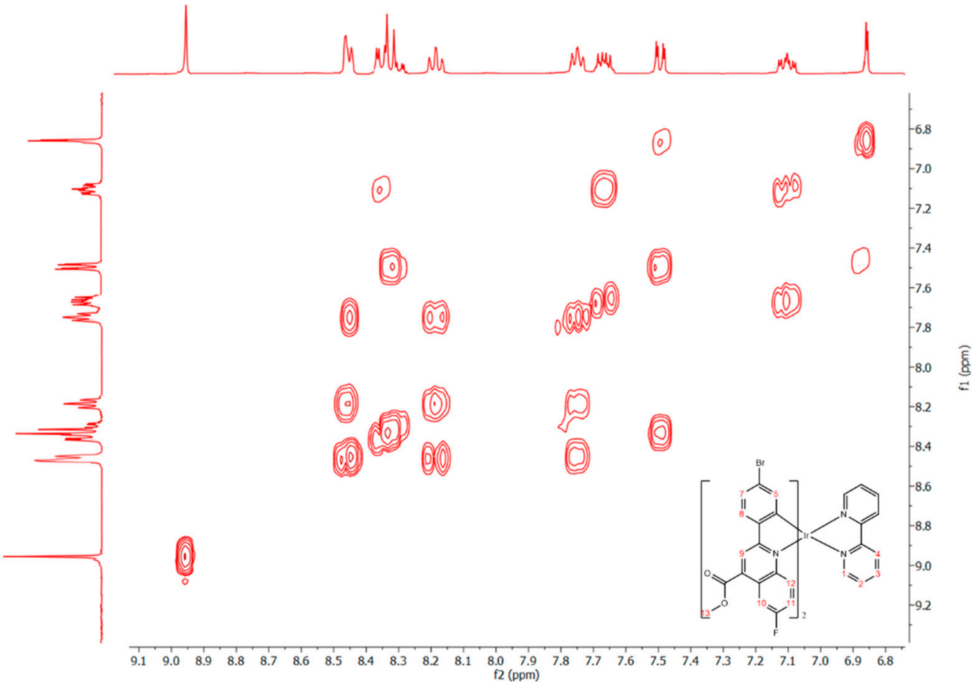

Figure S16. ^{1}H-^{1}H COSY NMR spectrum of Complex Ir2-F in Acetone-d_6 at 298 K.

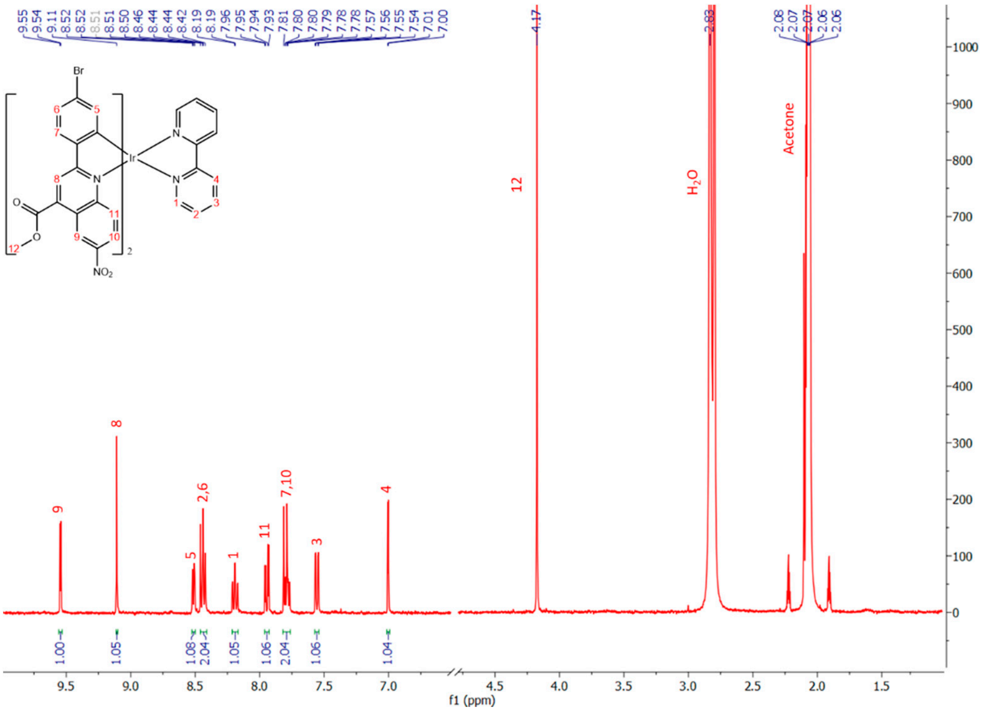

Figure S17. ^{1}H NMR spectrum of Complex Ir2-NO_2 in Acetone-d_6 at 298 K.

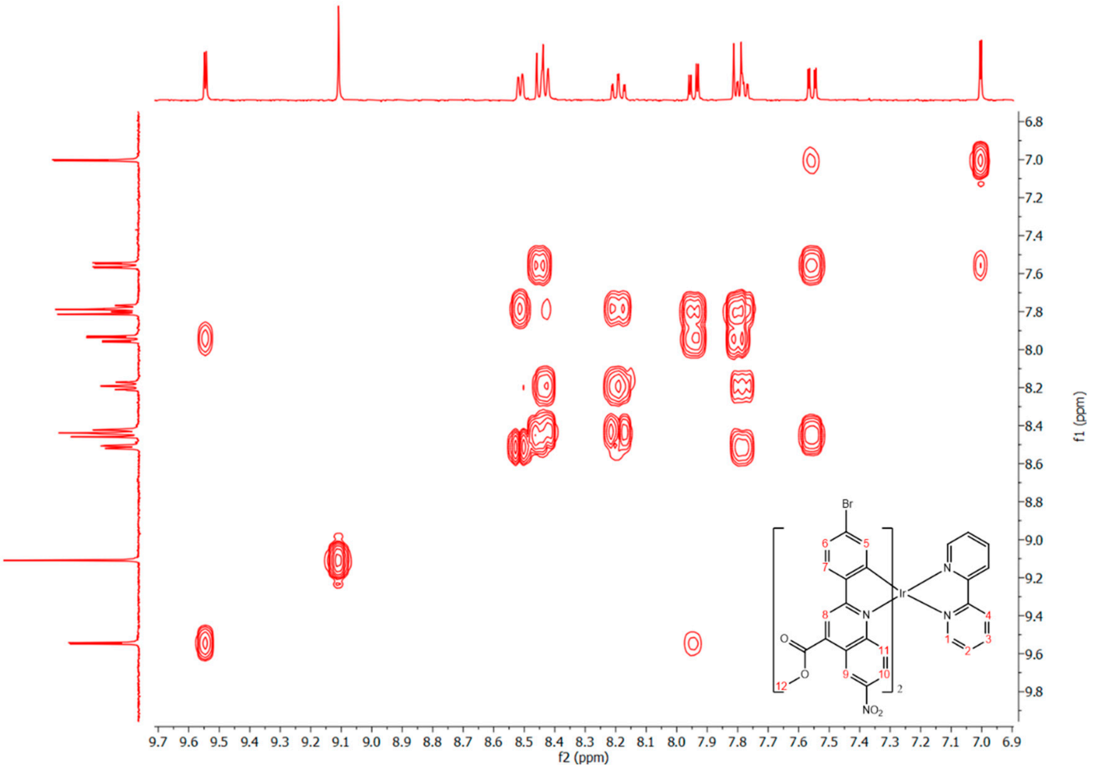

Figure S18. ^{1}H-^{1}H COSY NMR spectrum of Complex Ir2-NO_2 in DMSO-d_6 at 298 K.

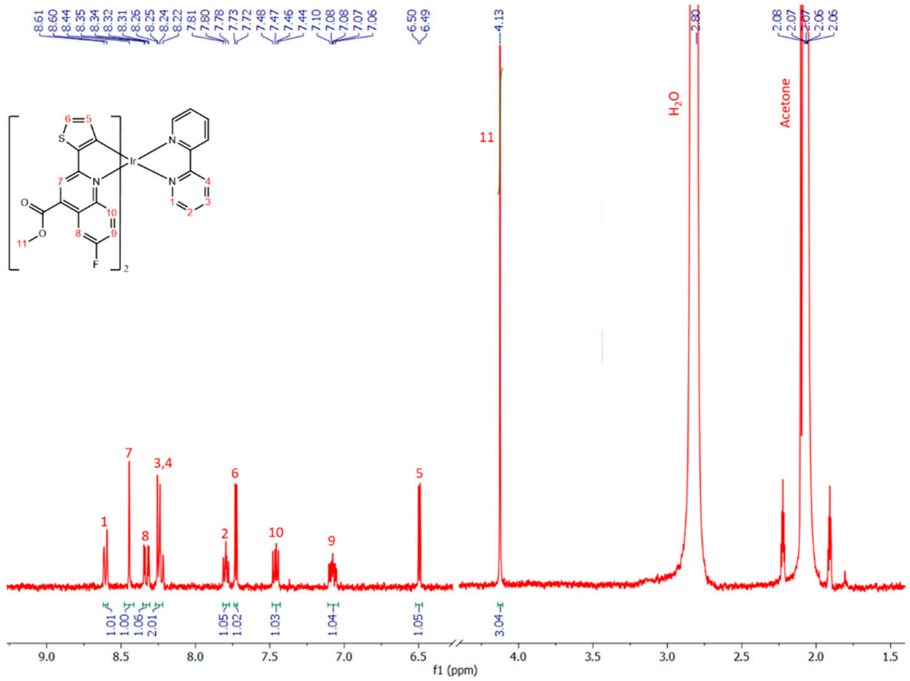

Figure S19. ^{1}H NMR spectrum of Complex Ir3-F in Acetone-d_6 at 298 K.

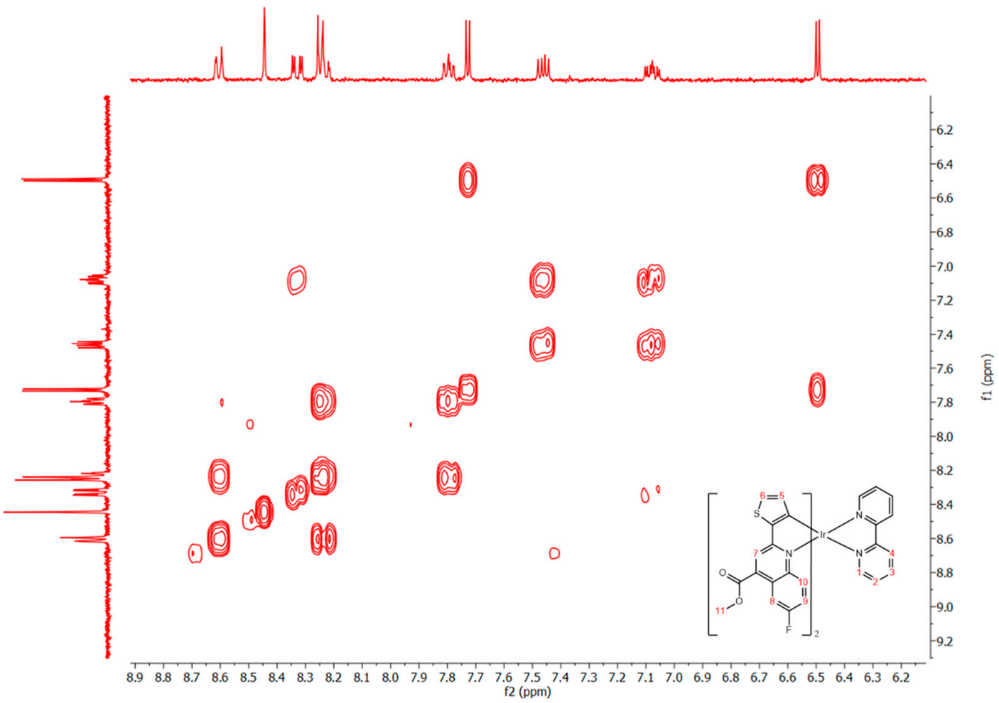

Figure S20. ^{1}H-^{1}H COSY NMR spectrum of Complex Ir3-F in Acetone-d_6 at 298 K.

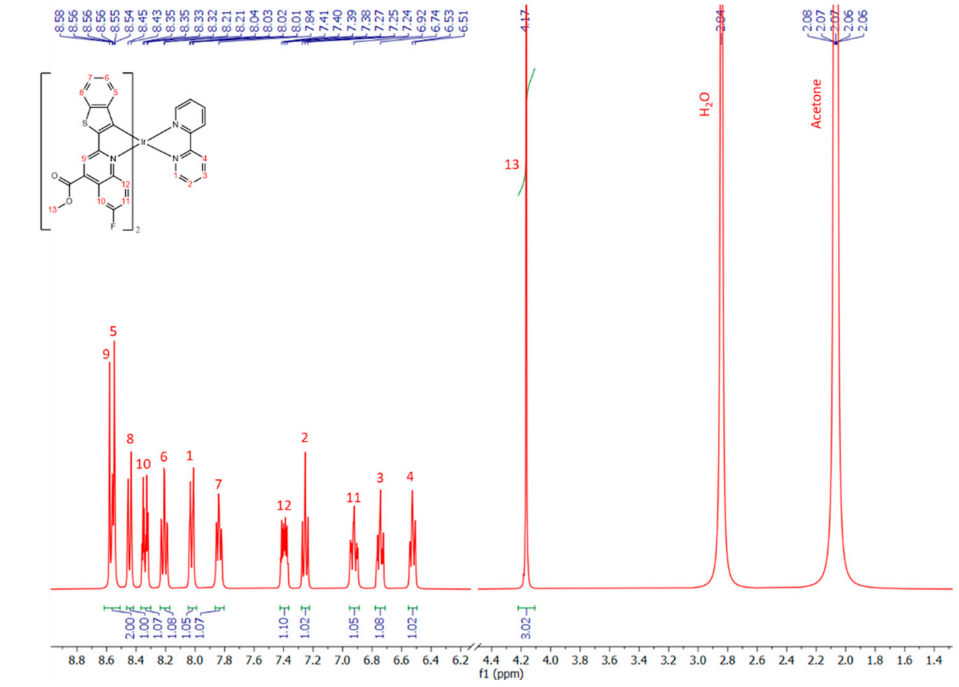

Figure S21. ^{1}H NMR spectrum of Complex Ir4-F in Acetone-d_6 at 298 K.

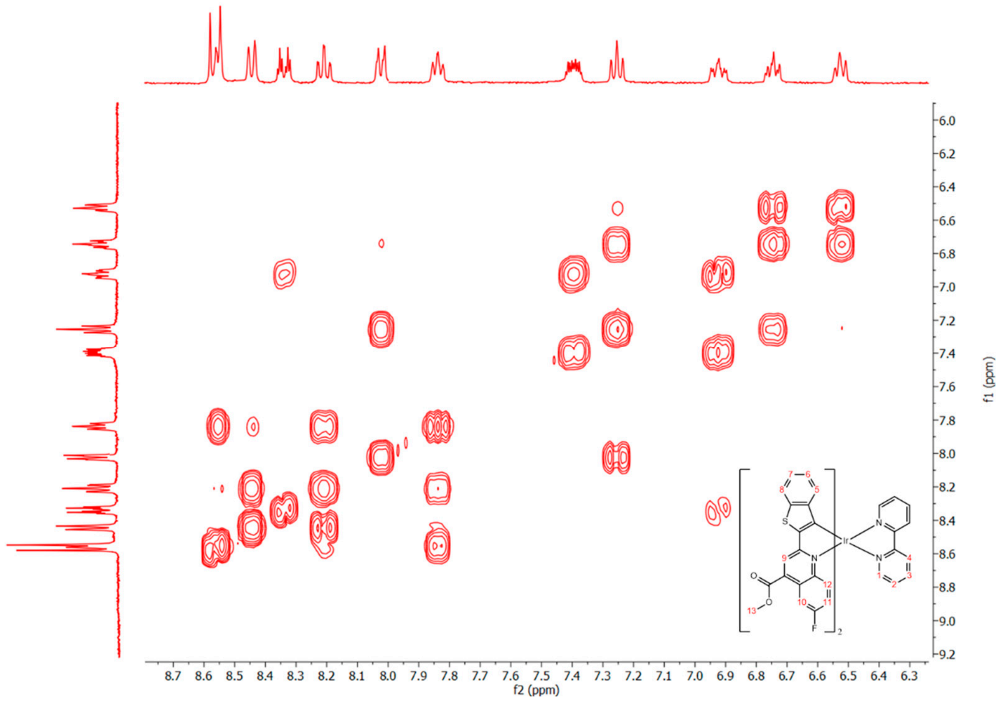

Figure S22. ^{1}H-^{1}H COSY NMR spectrum of Complex Ir4-F in Acetone-d_6 at 298 K.

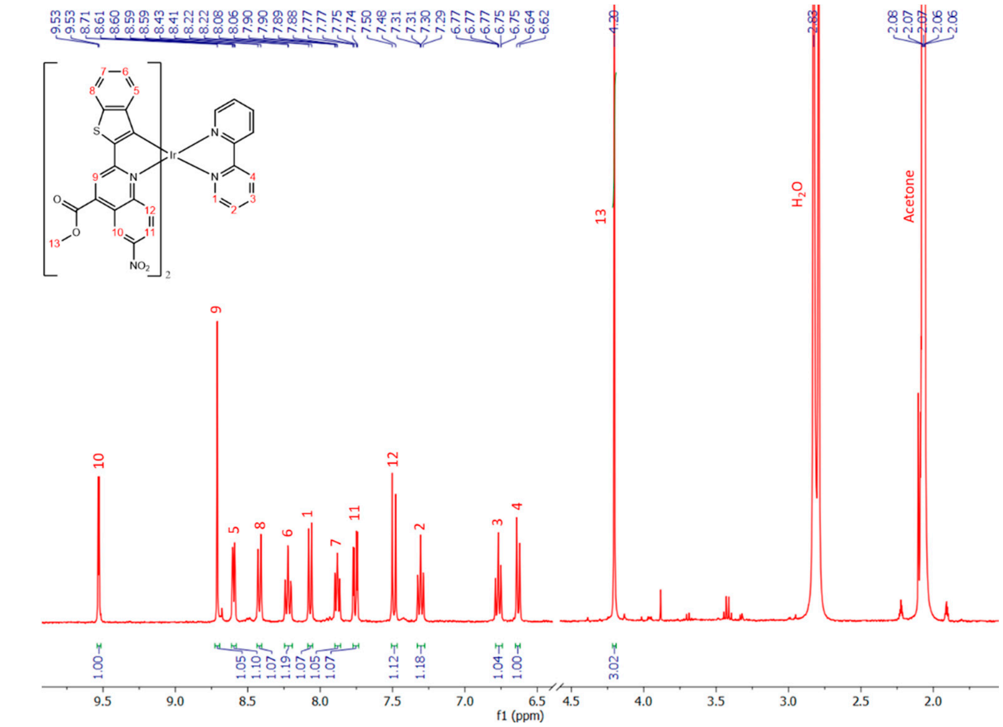

Figure S23. ^{1}H NMR spectrum of complex Ir4-NO_2 in Acetone-d_6 at 298 K.

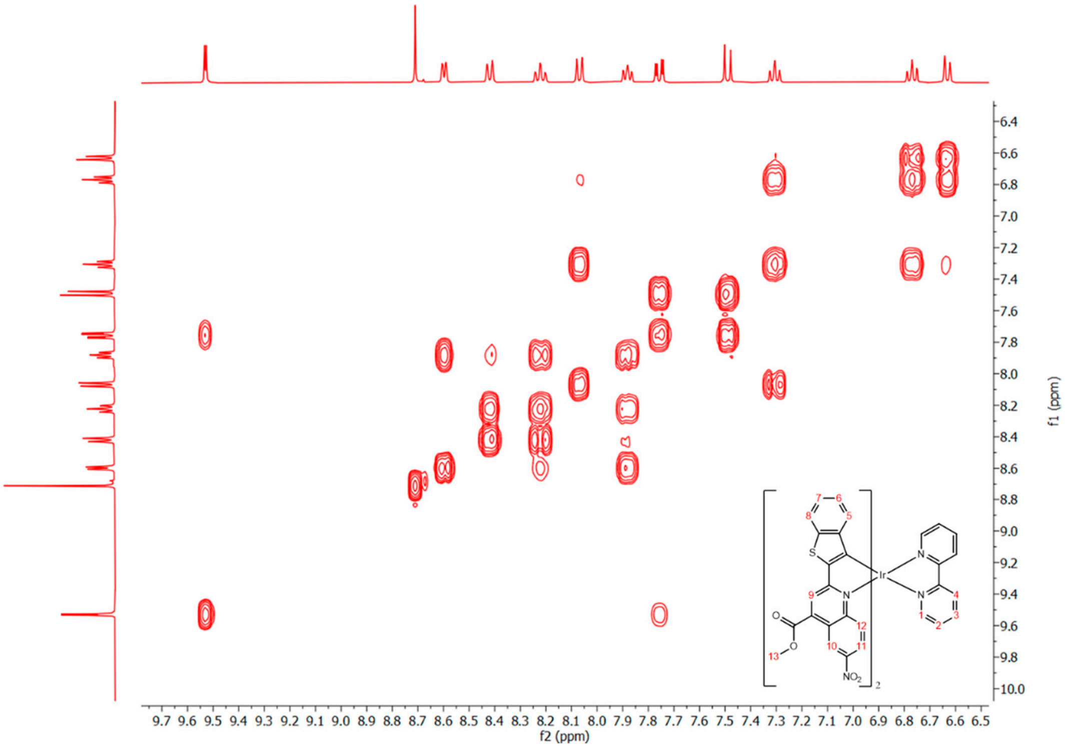

Figure S24. ^{1}H-^{1}H COSY NMR spectrum of Complex Ir4-NO_2 in Acetone-d_6 at 298 K.

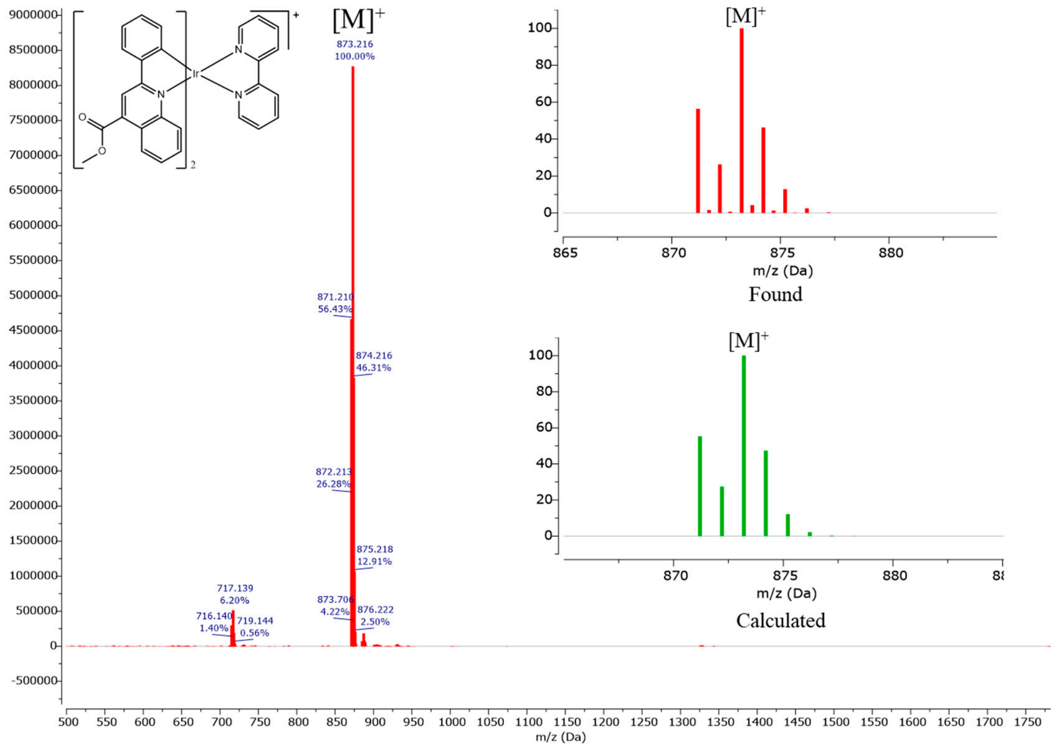

Figure S25. ESI$^+$ mass spectrum of complex Ir1-H.

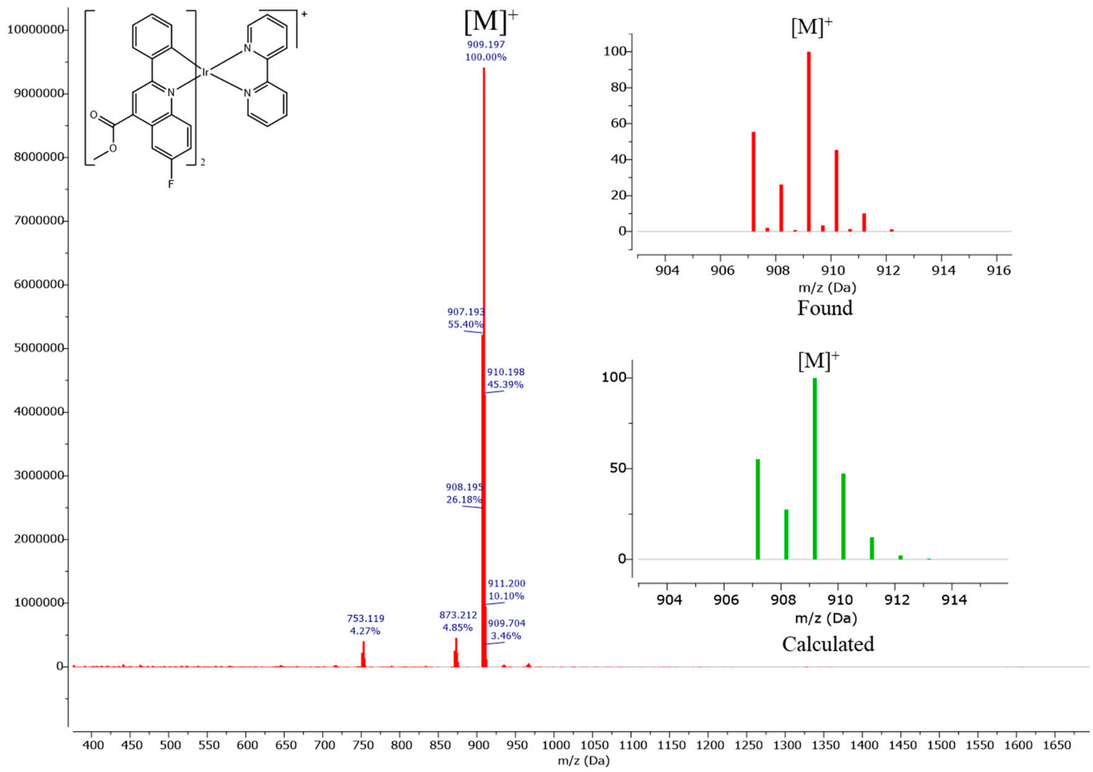

Figure S26. ESI$^+$ mass spectrum of complex Ir1-F.

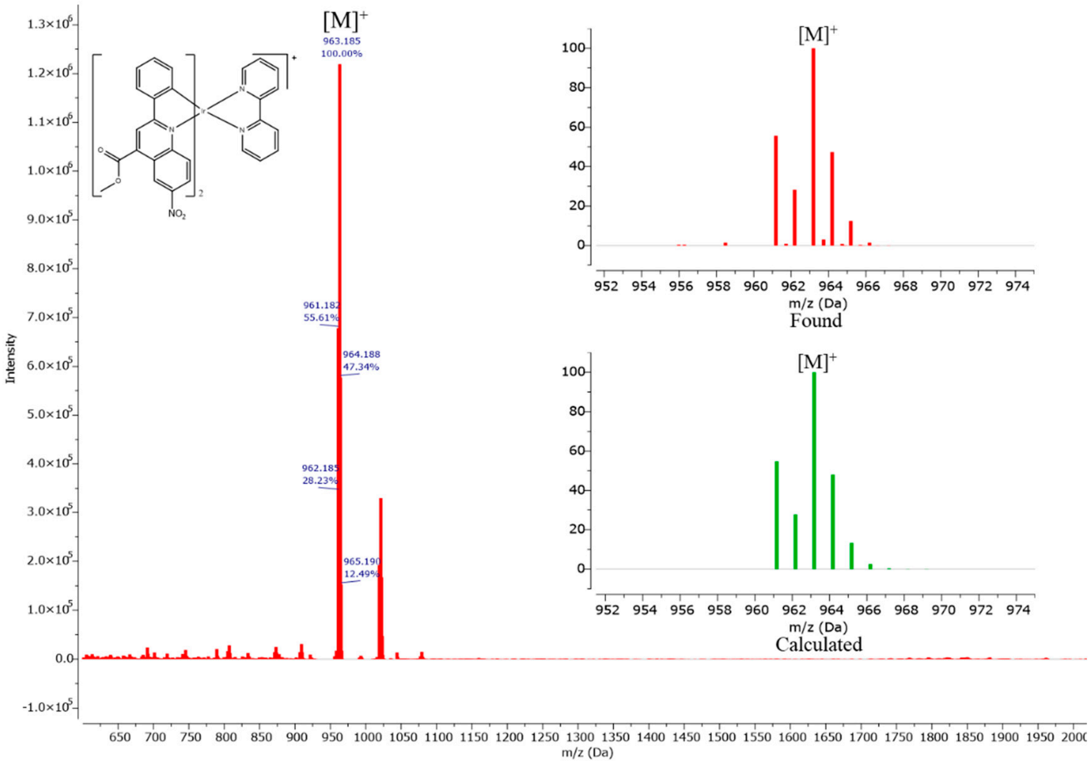

Figure S27. ESI$^+$ mass spectrum of complex Ir1-NO_2.

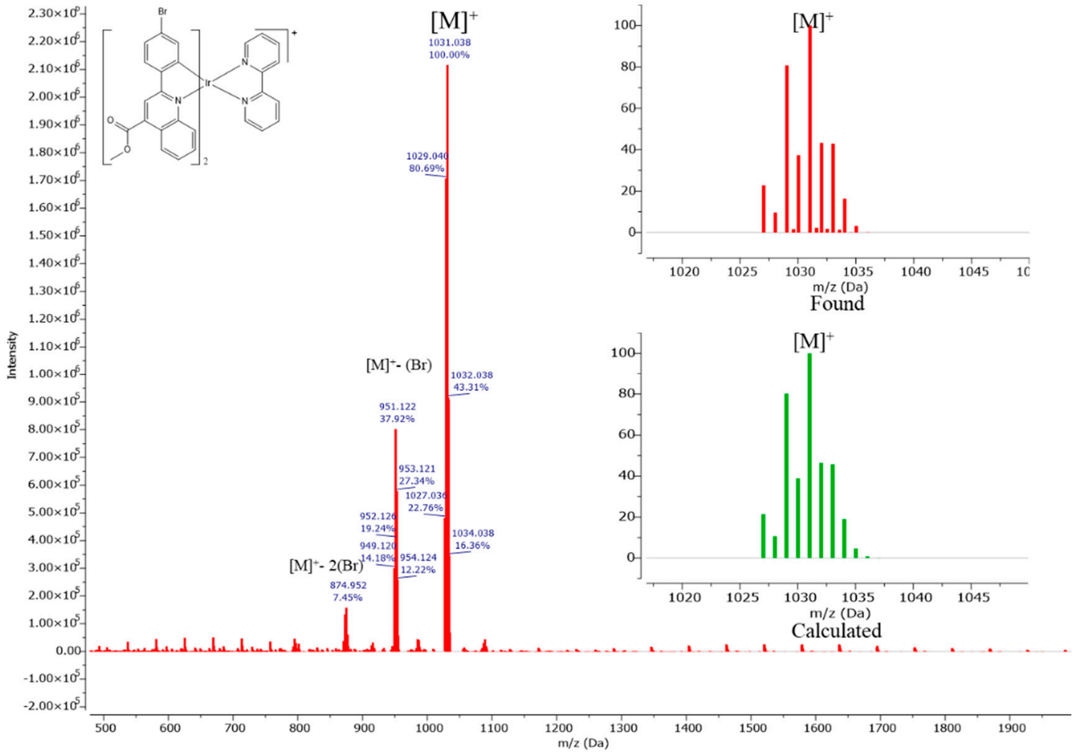

Figure S28. ESI$^+$ mass spectrum of complex Ir2-H.

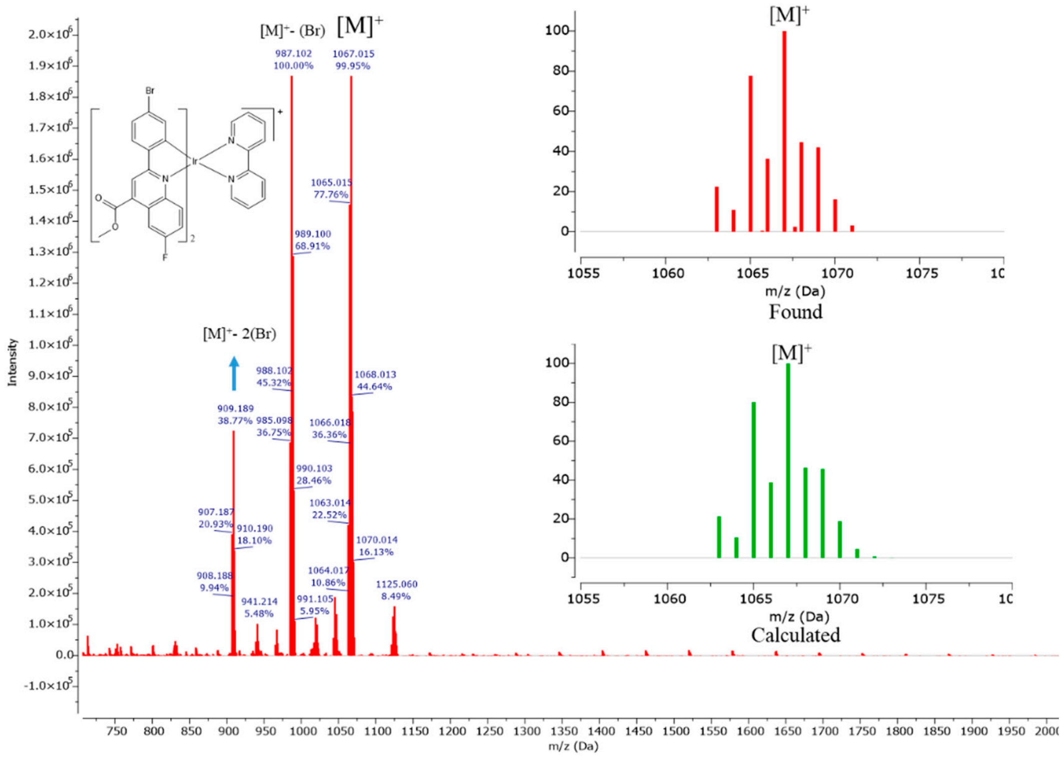

Figure S29. ESI$^+$ mass spectrum of complex Ir2-F.

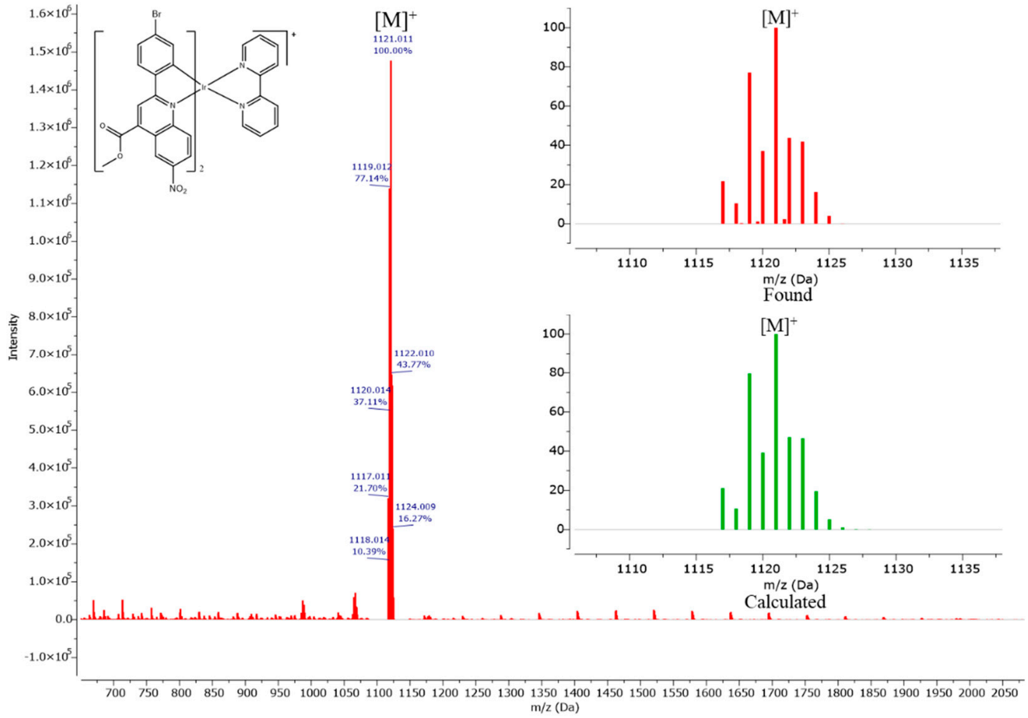

Figure S30. ESI$^+$ mass spectrum of complex Ir2-NO_2.

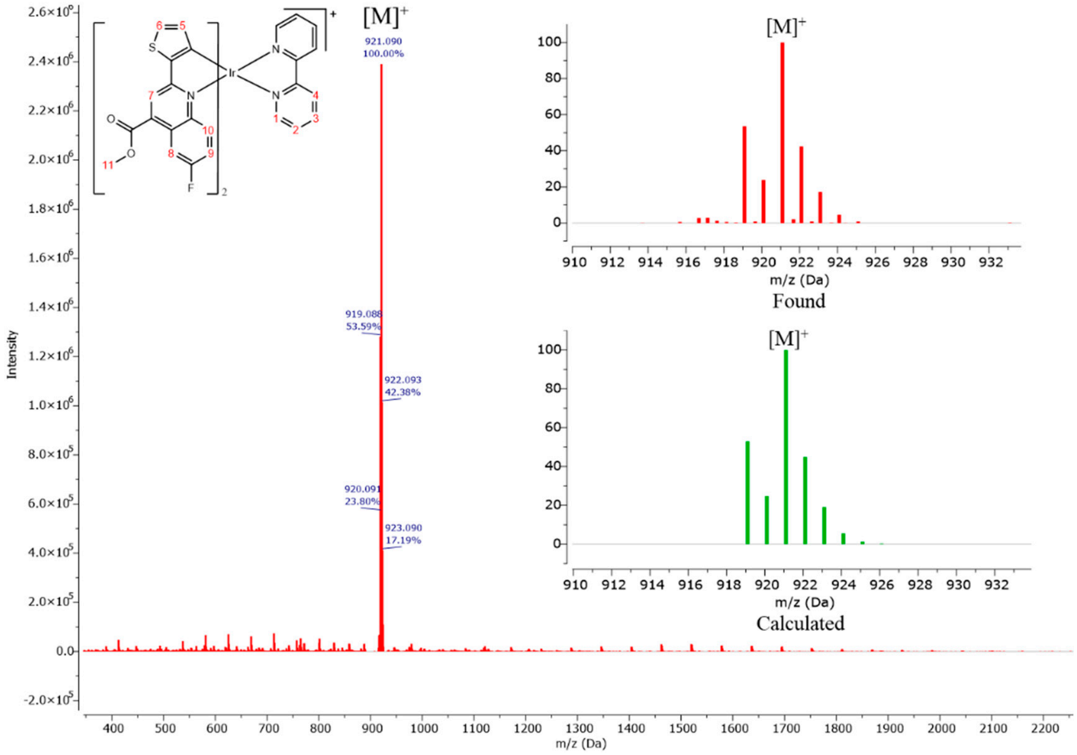

Figure S31. ESI$^+$ mass spectrum of complex Ir3-F.

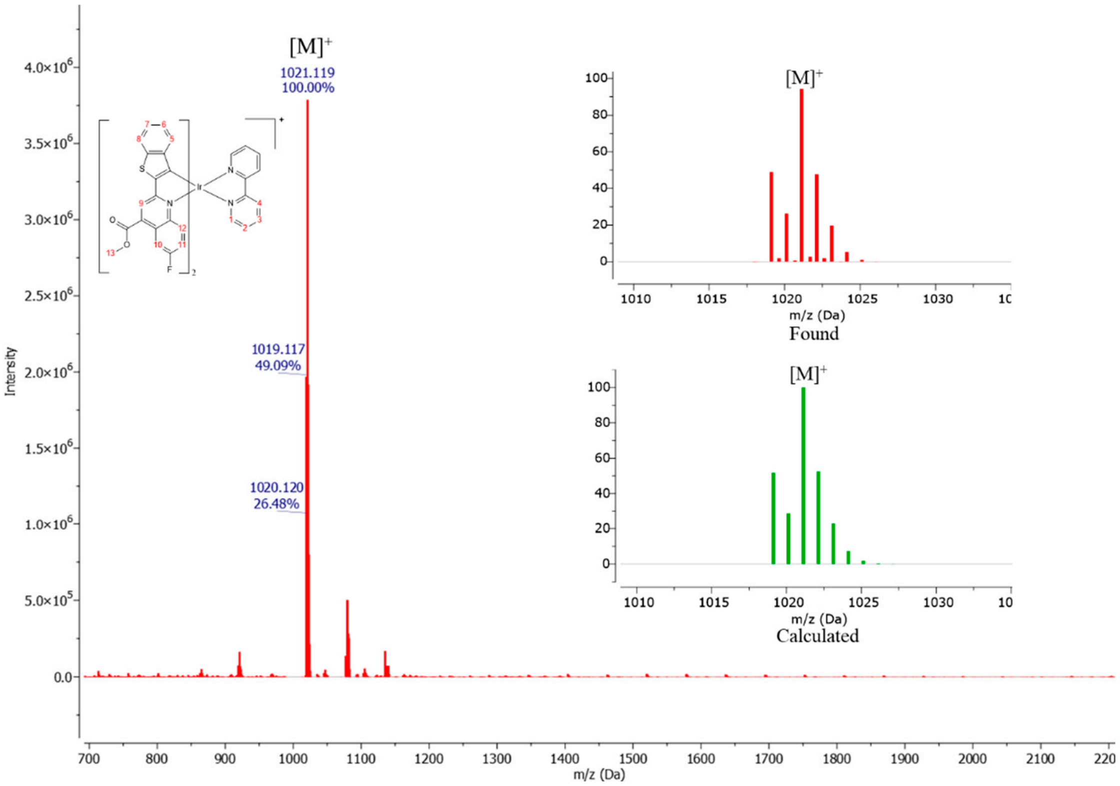

Figure S32. ESI$^+$ mass spectrum of complex Ir4-F.

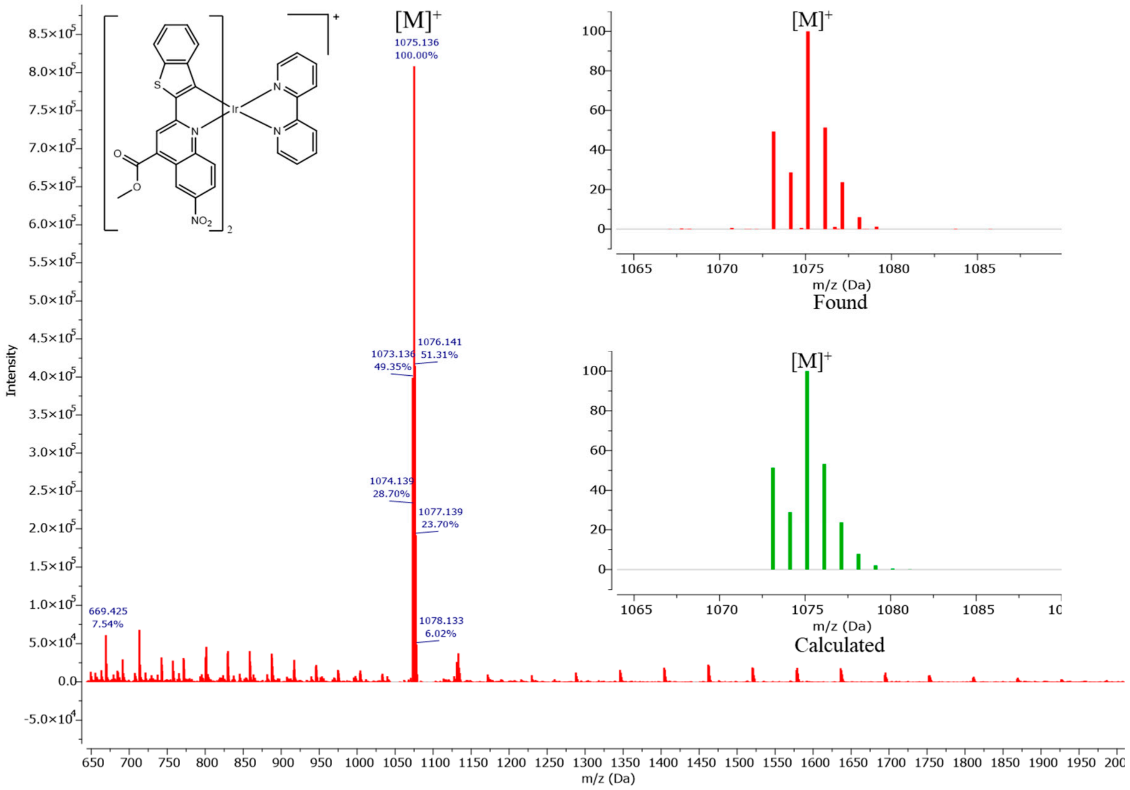

Figure S33. ESI$^+$ mass spectrum of complex Ir4-NO_2.

Part 3. Photophysical data and computational results.

(**a**) (**b**) (**c**)

Figure S34. Absorption spectra of all synthesized complexes in DCM: (a) Ir1-H, Ir1-F, Ir1-NO_2; (b) Ir2-H, Ir2-F, Ir2-NO_2; (c) Ir3-F, Ir4-F, Ir4-NO_2.

Complex Ir1-H

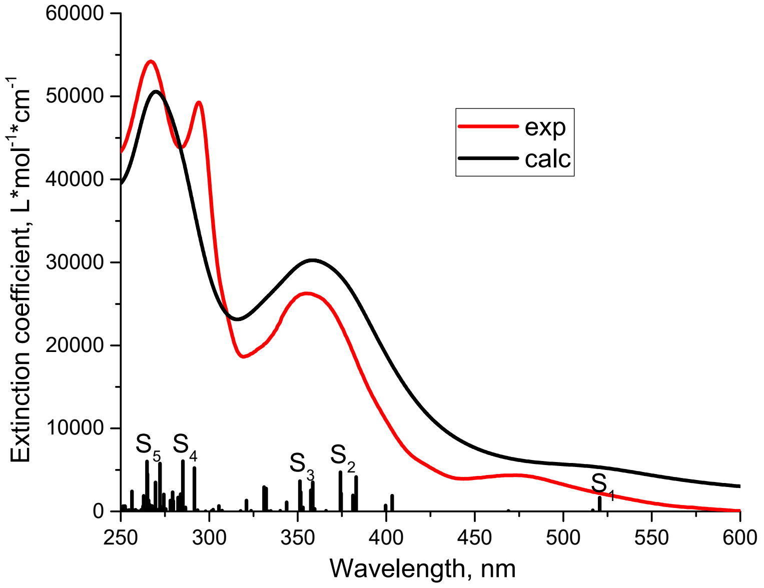

Figure S35. Absorption spectra of **Ir1-H**: experimental (red) and calculated (black) lines with oscillator strengths of electronic transitions (bars).

Table S2. Experimental and calculated absorption maxima (λ), extinction coefficients (ε), oscillator strengths (f) **Ir1-H**.

λ, nm (exp)	$\varepsilon*10^{-3}$, $M^{-1}cm^{-1}$ (exp)	Transitions	λ, nm (calc)	*f* (calc)	Contribution of main NTO pair in transition (%)
267	54	$S_0 \rightarrow S_5$	265	0.16	44
293	49	$S_0 \rightarrow S_4$	285	0.16	37
355	26	$S_0 \rightarrow S_3$	351	0.10	54
		$S_0 \rightarrow S_2$	374	0.13	72
473	5	$S_0 \rightarrow S_1$	521	0.05	98

Table S3. The decrease (violet) and increase (terracota) of electron density for most intensive electronic absorption transitions of **Ir1-H**. The data for the corresponding interfragment charge transfer (IFCT) are given below the figures. Diagonal values represent intraligand transitions, off-diagonal values represent a charge transfer from “Donor” to “Acceptor”.

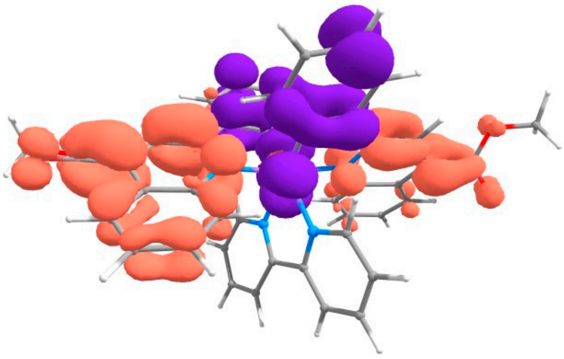

$S_0 \rightarrow S_1$

Donor	Acceptor			
	Ir	N^N	N^C	N^C’
Ir	0.010	0.005	0.131	0.235
N^N	0.001	0.000	0.008	0.014
N^C	0.008	0.004	0.103	0.185
N^C’	0.008	0.004	0.103	0.184

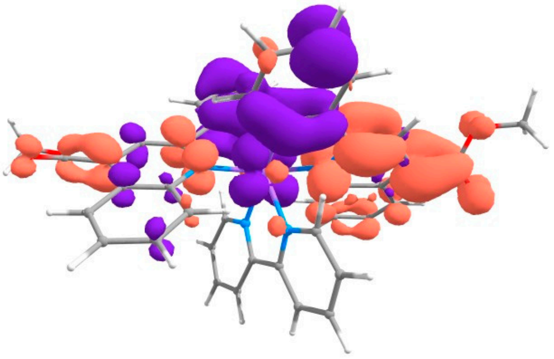

$S_0 \rightarrow S_2$

Donor	Acceptor			
	Ir	N^N	N^C	N^C’
Ir	0.005	0.043	0.106	0.034
N^N	0.001	0.007	0.018	0.006
N^C	0.011	0.090	0.218	0.069
N^C’	0.011	0.091	0.221	0.070

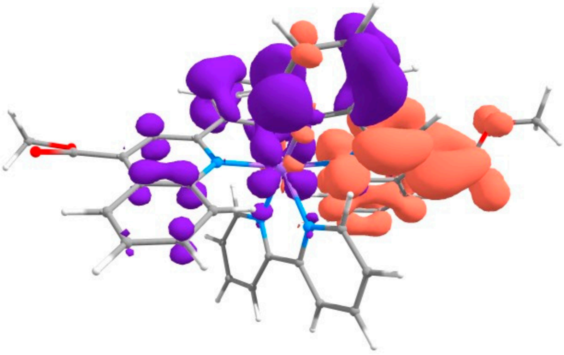

$S_0 \rightarrow S_3$

Donor	Acceptor			
	Ir	N^N	C^N	C^N’
Ir	0.006	0.065	0.141	0.011
N^N	0.001	0.009	0.019	0.001
N^C	0.010	0.111	0.240	0.019
N^C’	0.010	0.107	0.231	0.018

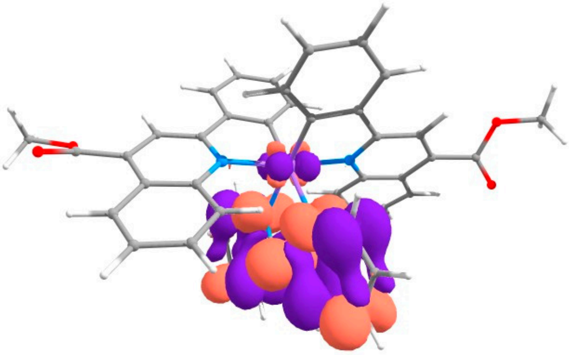

$S_0 \rightarrow S_4$

Donor	Acceptor			
	Ir	N^N	N^C	N^C’
Ir	0.005	0.073	0.038	0.035
N^N	0.013	0.197	0.104	0.095
N^C	0.007	0.107	0.056	0.051
N^C’	0.007	0.106	0.056	0.051

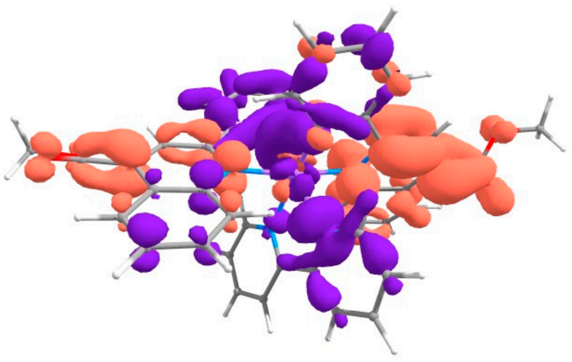

$S_0 \rightarrow S_5$

Donor	Acceptor			
	Ir	N^N	N^C	N^C’
Ir	0.004	0.028	0.078	0.028
N^N	0.004	0.034	0.097	0.034
N^C	0.007	0.053	0.150	0.053
N^C’	0.011	0.087	0.247	0.087

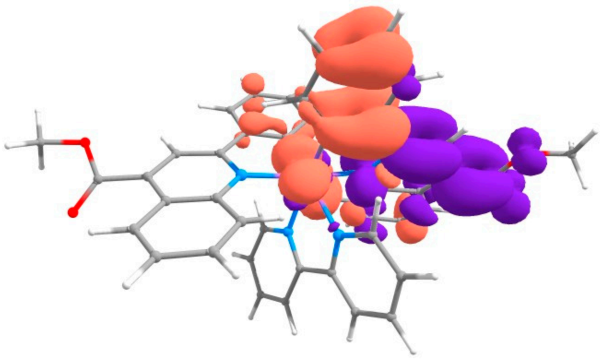

$T_1 \rightarrow S_0$

Donor	Acceptor			
	Ir	N^N	N^C	N^C’
Ir	0.010	0.001	0.003	0.025
N^N	0.003	0.000	0.001	0.007
N^C	0.001	0.000	0.000	0.002
N^C’	0.252	0.014	0.078	0.602

Complex Ir1-F

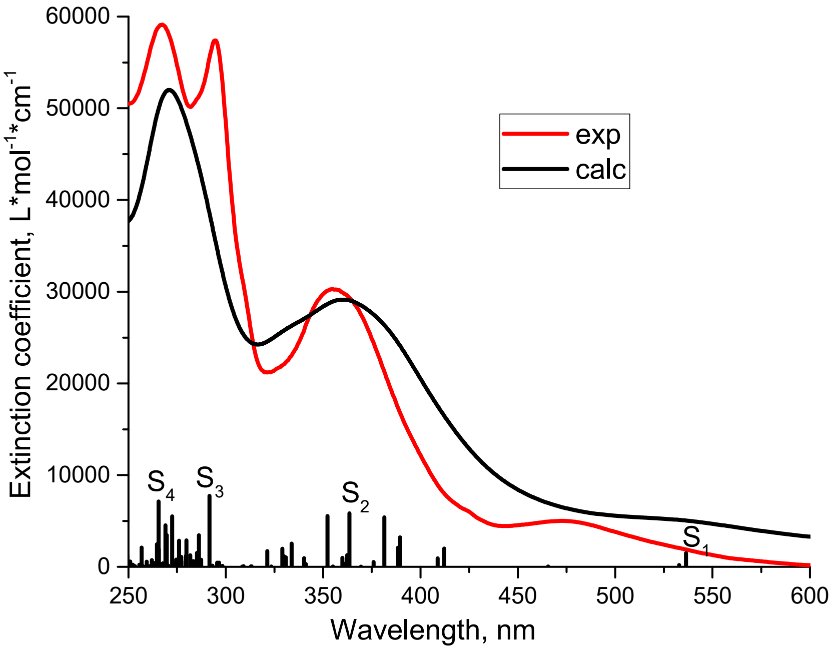

Figure S36. Absorption spectra of **Ir1-F**: experimental (red) and calculated (black) lines with oscillator strengths of electronic transitions (bars).

Table S4. Experimental and calculated absorption maxima (λ), extinction coefficients (ε), oscillator strengths (f) **Ir1-F**.

λ, nm (exp)	ε*10^{-3}, $M^{-1}cm^{-1}$ (exp)	Transitions	λ, nm (calc)	*f* (calc)	Contribution of main NTO pair in transition (%)
268	59	$S_0 \rightarrow S_4$	265	0.19	25
295	57	$S_0 \rightarrow S_3$	292	0.21	64
357	30	$S_0 \rightarrow S_2$	364	0.16	67
474	5	$S_0 \rightarrow S_1$	536	0.04	98

Table S5. The decrease (violet) and increase (terracota) of electron density for most intensive electronic absorption transitions of **Ir1-F**. The data for the corresponding interfragment charge transfer (IFCT) are given below the figures. Diagonal values represent intraligand transitons, off-diagonal values represent a charge transfer from "Donor" to "Acceptor".

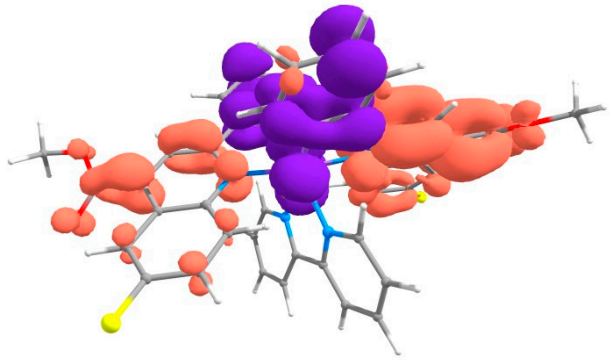

$S_0 \rightarrow S_1$

Donor	Acceptor			
	Ir	N^N	N^C	N^C'
Ir	0.009	0.004	0.105	0.254
N^N	0.001	0.000	0.006	0.015
N^C	0.008	0.003	0.086	0.207
N^C'	0.008	0.003	0.086	0.206

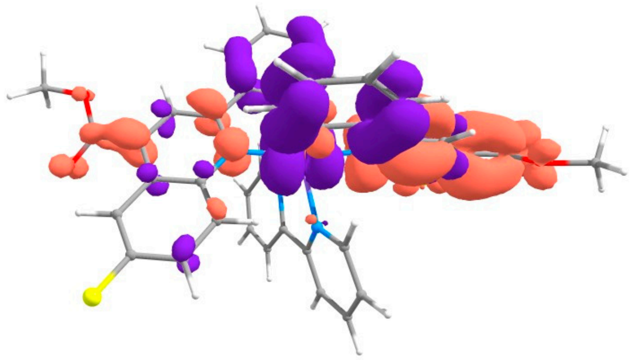

$S_0 \rightarrow S_2$

Donor	Acceptor			
	Ir	N^N	N^C	N^C'
Ir	0.008	0.025	0.079	0.176
N^N	0.001	0.002	0.007	0.016
N^C	0.008	0.025	0.079	0.177
N^C'	0.010	0.035	0.109	0.243

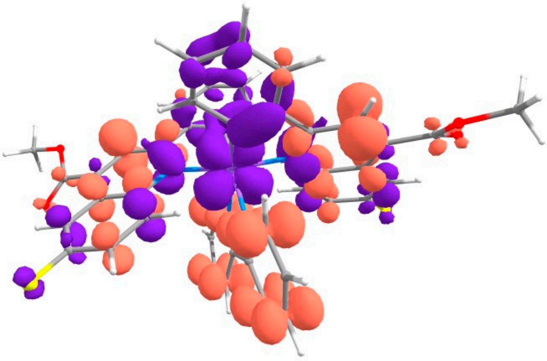

$S_0 \rightarrow S_3$

Donor	Acceptor			
	Ir	N^N	N^C	N^C'
Ir	0.007	0.101	0.069	0.067
N^N	0.003	0.050	0.034	0.033
N^C	0.008	0.131	0.089	0.086
N^C'	0.009	0.133	0.091	0.088

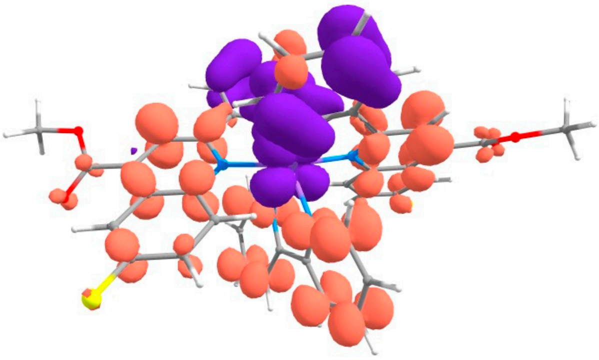

$S_0 \rightarrow S_4$

Donor	Acceptor			
	Ir	N^N	N^C	N^C'
Ir	0.007	0.104	0.067	0.094
N^N	0.002	0.032	0.021	0.029
N^C	0.008	0.128	0.083	0.116
N^C'	0.008	0.118	0.078	0.107

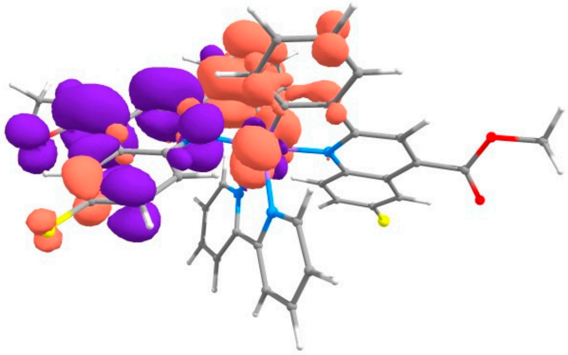

$T_1 \rightarrow S_0$

Donor	Acceptor			
	Ir	N^N	N^C	N^C'
Ir	0.010	0.001	0.003	0.026
N^N	0.003	0.000	0.001	0.007
N^C	0.001	0.000	0.000	0.002
N^C'	0.243	0.014	0.080	0.610

Complex Ir1-NO_2

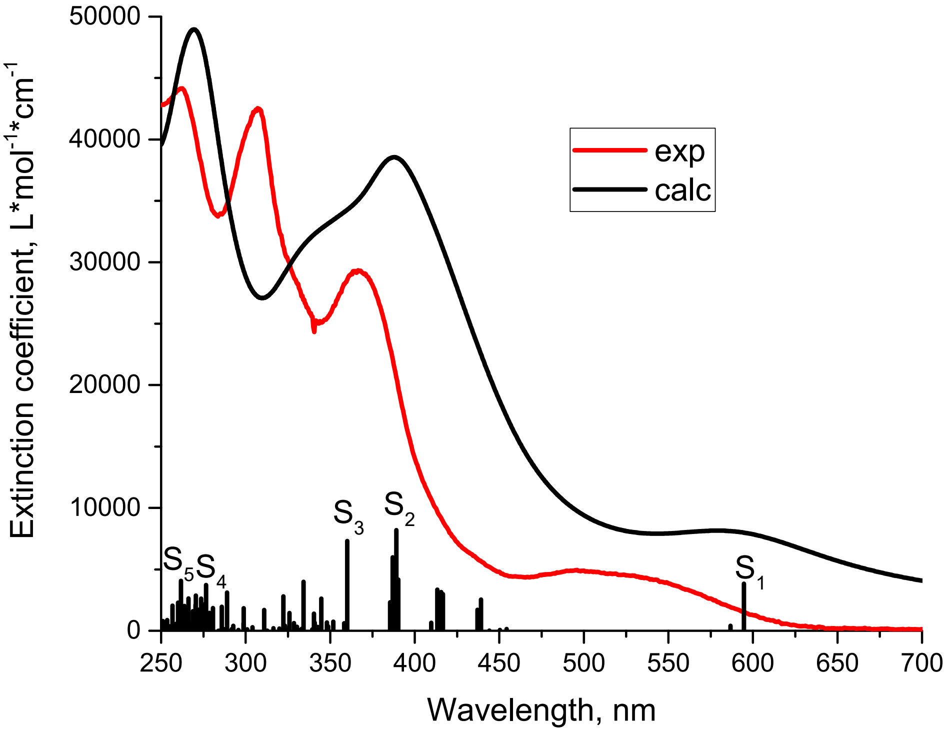

Figure S37. Absorption spectra of **Ir1-NO_2**: experimental (red) and calculated (black) lines with oscillator strengths of electronic transitions (bars).

Table S6. Experimental and calculated absorption maxima (λ), extinction coefficients (ε), oscillator strengths (f) **Ir1-NO_2**.

λ, nm (exp)	$\varepsilon*10^{-3}$, $M^{-1}cm^{-1}$ (exp)	Transitions	λ, nm (calc)	*f* (calc)	Contribution of main NTO pair in transition (%)
262	44	$S_0 \rightarrow S_5$	262	0.11	86
		$S_0 \rightarrow S_4$	277	0.10	56
308	43	$S_0 \rightarrow S_3$	360	0.20	78
366	29	$S_0 \rightarrow S_2$	389	0.22	91
511	5	$S_0 \rightarrow S_1$	595	0.10	98

Table S7. The decrease (violet) and increase (terracota) of electron density for most intensive electronic absorption transitions of **Ir1-NO₂**. The data for the corresponding interfragment charge transfer (IFCT) are given below the figures. Diagonal values represent intraligand transitons, off-diagonal values represent a charge transfer from "Donor" to "Acceptor".

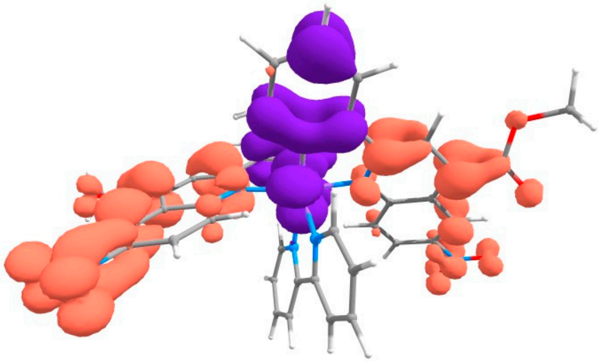

$S_0 \rightarrow S_1$

Donor	Acceptor			
	Ir	N^N	N^C	N^C'
Ir	0.006	0.002	0.138	0.235
N^N	0.000	0.000	0.008	0.014
N^C	0.004	0.002	0.108	0.185
N^C'	0.004	0.002	0.108	0.184

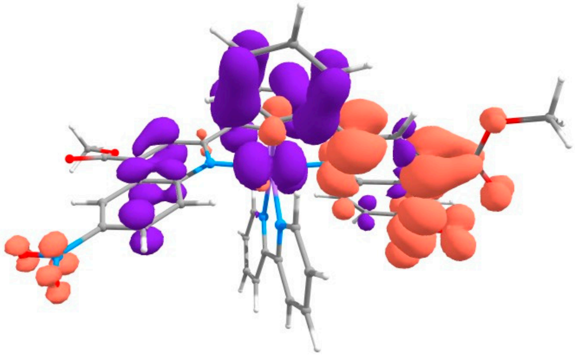

$S_0 \rightarrow S_2$

Donor	Acceptor			
	Ir	N^N	N^C	N^C'
Ir	0.002	0.001	0.159	0.015
N^N	0.000	0.000	0.023	0.002
N^C	0.004	0.002	0.309	0.028
N^C'	0.006	0.002	0.409	0.037

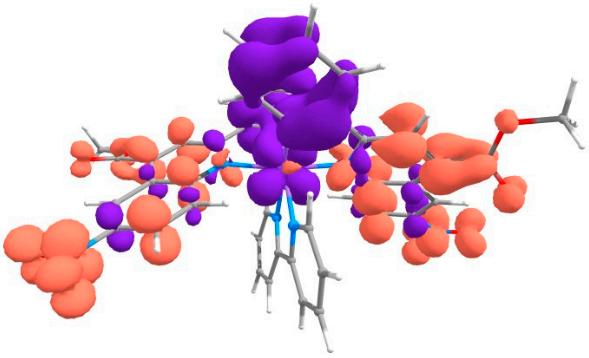

$S_0 \rightarrow S_3$

Donor	Acceptor			
	Ir	N^N	N^C	N^C'
Ir	0.004	0.001	0.129	0.094
N^N	0.001	0.000	0.023	0.017
N^C	0.007	0.002	0.223	0.161
N^C'	0.006	0.002	0.190	0.138

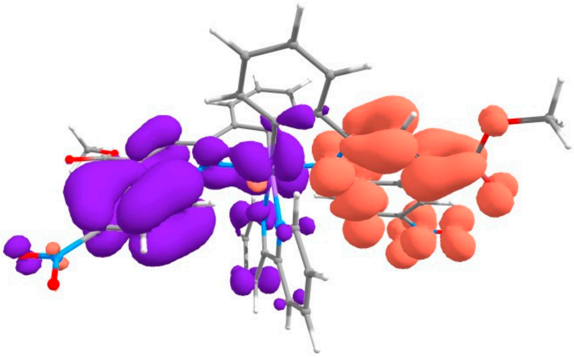

$S_0 \rightarrow S_4$

Donor	Acceptor			
	Ir	N^N	N^C	N^C'
Ir	0.003	0.010	0.131	0.020
N^N	0.002	0.006	0.082	0.013
N^C	0.003	0.010	0.126	0.019
N^C'	0.010	0.036	0.459	0.070

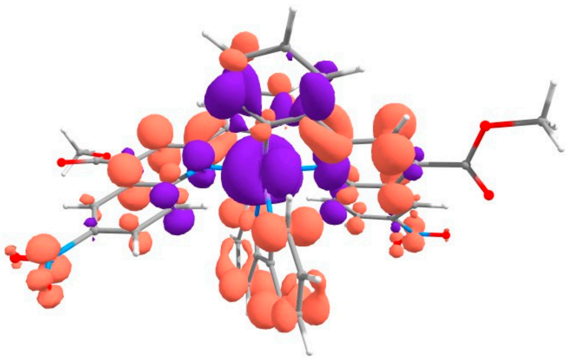

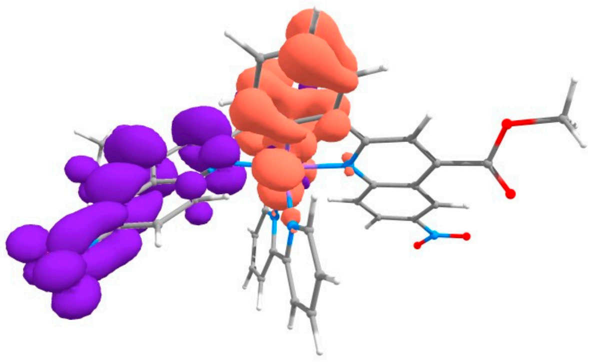

$S_0 \rightarrow S_5$

Donor	Acceptor			
	Ir	N^N	N^C	N^C’
Ir	0.005	0.116	0.165	0.168
N^N	0.000	0.005	0.007	0.007
N^C	0.003	0.077	0.109	0.111
N^C’	0.003	0.058	0.082	0.084

$T_1 \rightarrow S_0$

Donor	Acceptor			
	Ir	N^N	N^C	N^C’
Ir	0.010	0.001	0.006	0.012
N^N	0.003	0.000	0.002	0.004
N^C	0.001	0.000	0.001	0.001
N^C’	0.336	0.023	0.198	0.404

Complex Ir2-H

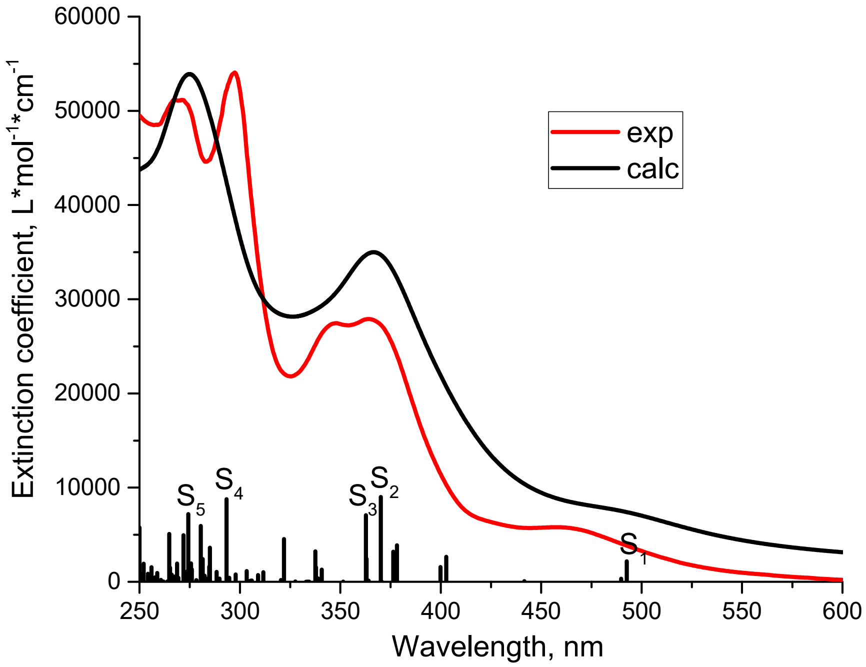

Figure S38. Absorption spectra of **Ir2-H**: experimental (red) and calculated (black) lines with oscillator strengths of electronic transitions (bars).

Table S8. Experimental and calculated absorption maxima (λ), extinction coefficients (ε), oscillator strengths (f) **Ir2-H**.

λ, nm (exp)	ε*10⁻³, M⁻¹cm⁻¹ (exp)	Transitions	λ, nm (calc)	*f* (calc)	Contribution of main NTO pair in transition (%)
267	51	$S_0 \rightarrow S_5$	274	0.19	52
297	54	$S_0 \rightarrow S_4$	293	0.24	41
348	28	$S_0 \rightarrow S_3$	363	0.19	90
365	28	$S_0 \rightarrow S_2$	370	0.24	95
460	9	$S_0 \rightarrow S_1$	493	0.06	97

Table S9. The decrease (violet) and increase (terracota) of electron density for most intensive electronic absorption transitions of **Ir2-H**. The data for the corresponding interfragment charge transfer (IFCT) are given below the figures. Diagonal values represent intraligand transitons, off-diagonal values represent a charge transfer from "Donor" to "Acceptor".

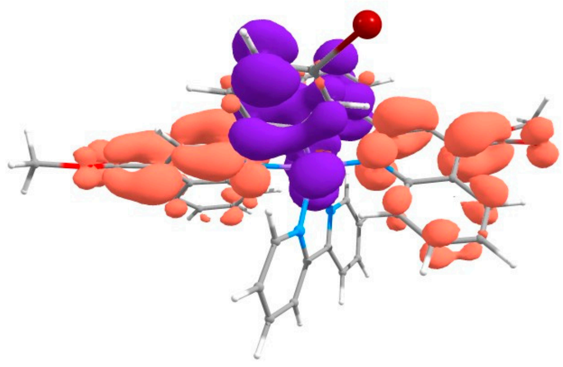

$S_0 \rightarrow S_1$

Donor	Acceptor			
	Ir	N^N	N^C	N^C'
Ir	0.010	0.004	0.180	0.165
N^N	0.001	0.000	0.011	0.010
N^C	0.008	0.004	0.155	0.142
N^C'	0.008	0.004	0.156	0.143

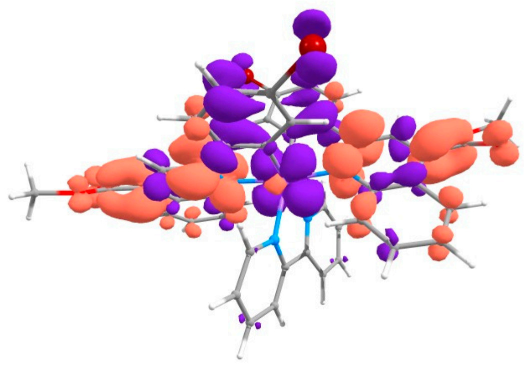

$S_0 \rightarrow S_2$

Donor	Acceptor			
	Ir	N^N	N^C	N^C'
Ir	0.009	0.005	0.145	0.159
N^N	0.001	0.001	0.024	0.026
N^C	0.008	0.005	0.142	0.155
N^C'	0.009	0.005	0.147	0.160

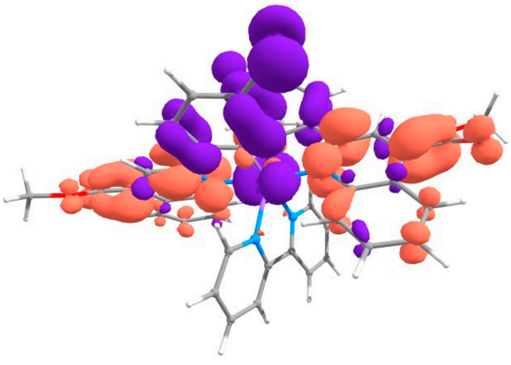

S$_0$→S$_3$

Donor	Acceptor			
	Ir	N^N	N^C	N^C'
Ir	0.006	0.005	0.102	0.093
N^N	0.001	0.001	0.012	0.011
N^C	0.011	0.010	0.193	0.176
N^C'	0.010	0.010	0.188	0.172

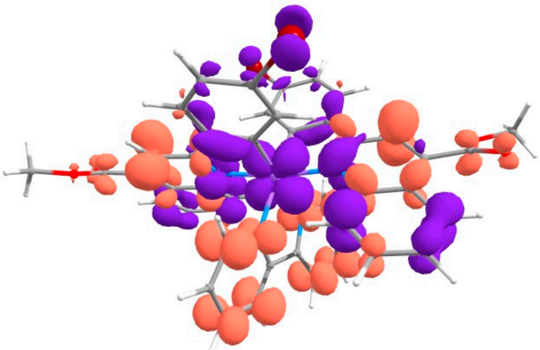

S$_0$→S$_4$

Donor	Acceptor			
	Ir	N^N	N^C	N^C'
Ir	0.005	0.037	0.058	0.058
N^N	0.001	0.006	0.009	0.009
N^C	0.012	0.095	0.150	0.149
N^C'	0.013	0.096	0.152	0.151

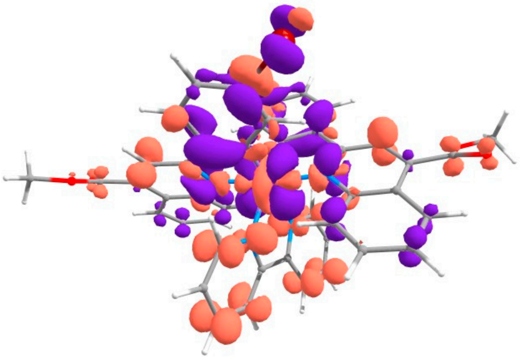

S$_0$→S$_5$

Donor	Acceptor			
	Ir	N^N	N^C	N^C'
Ir	0.058	0.065	0.098	0.095
N^N	0.008	0.009	0.014	0.014
N^C	0.059	0.065	0.098	0.096
N^C'	0.059	0.066	0.099	0.097

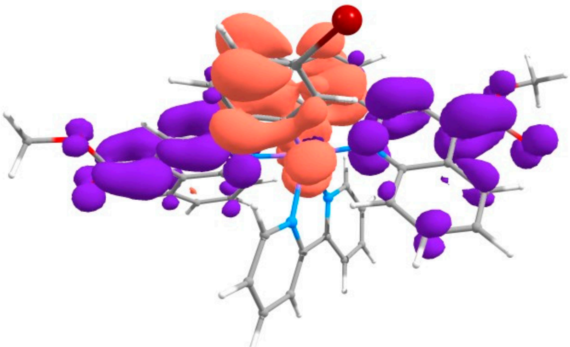

T$_1$→S$_0$

Donor	Acceptor			
	Ir	N^N	N^C	N^C'
Ir	0.007	0.001	0.010	0.010
N^N	0.003	0.000	0.004	0.004
N^C	0.119	0.009	0.166	0.165
N^C'	0.131	0.009	0.182	0.181

Complex Ir2-F

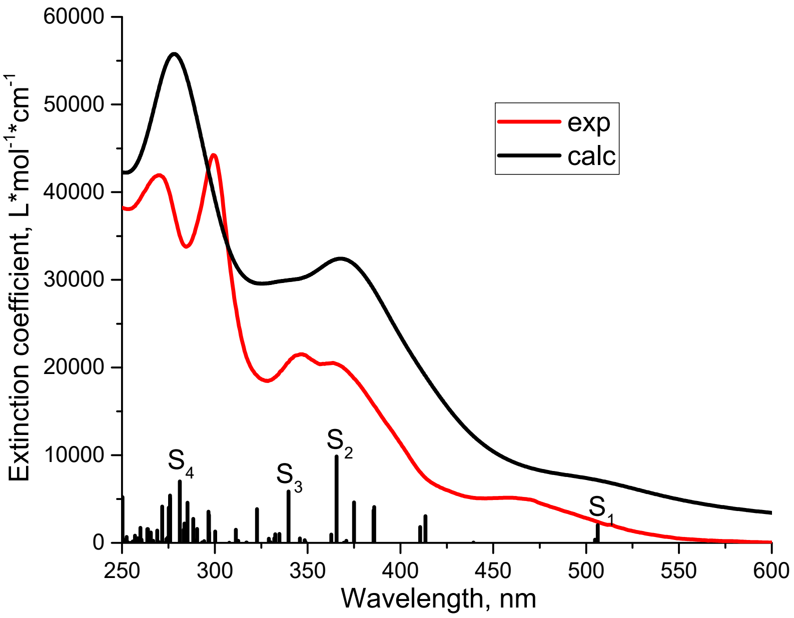

Figure S39. Absorption spectra of **Ir2-F**: experimental (red) and calculated (black) lines with oscillator strengths of electronic transitions (bars).

Table S10. Experimental and calculated absorption maxima (λ), extinction coefficients (ε), oscillator strengths (f) **Ir2-F**.

λ, nm (exp)	ε*10^{-3}, $M^{-1}cm^{-1}$ (exp)	Transitions	λ, nm (calc)	*f* (calc)	Contribution of main NTO pair in transition (%)
270	42	$S_0 \rightarrow S_4$	281	0.19	41
300	45				
345	22	$S_0 \rightarrow S_3$	340	0.16	90
365	21	$S_0 \rightarrow S_2$	366	0.27	86
466	5	$S_0 \rightarrow S_1$	506	0.05	96

Table S11. The decrease (violet) and increase (terracota) of electron density for most intensive electronic absorption transitions of **Ir2-F**. The data for the corresponding interfragment charge transfer (IFCT) are given below the figures. Diagonal values represent intraligand transitons, off-diagonal values represent a charge transfer from “Donor” to “Acceptor”.

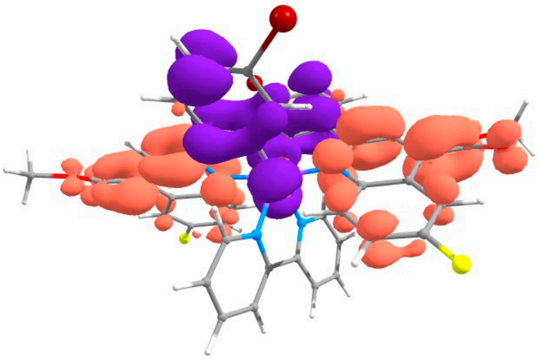

$S_0 \rightarrow S_1$

Donor	Acceptor			
	Ir	N^N	N^C	N^C’
Ir	0.009	0.003	0.174	0.161
N^N	0.001	0.000	0.010	0.009
N^C	0.008	0.003	0.158	0.147
N^C’	0.008	0.003	0.158	0.147

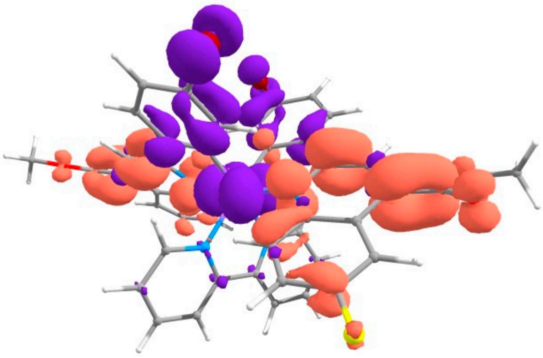

$S_0 \rightarrow S_2$

Donor	Acceptor			
	Ir	N^N	N^C	N^C’
Ir	0.010	0.009	0.188	0.176
N^N	0.001	0.001	0.026	0.024
N^C	0.008	0.007	0.140	0.132
N^C’	0.007	0.007	0.136	0.128

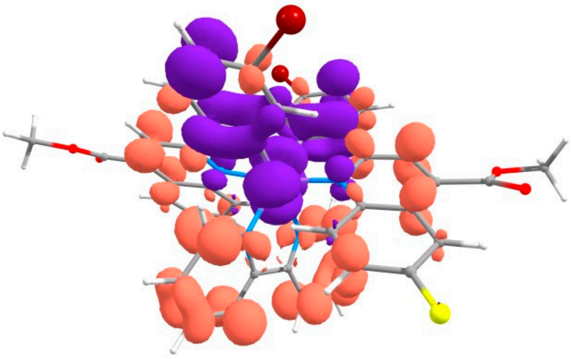

$S_0 \rightarrow S_3$

Donor	Acceptor			
	Ir	N^N	N^C	N^C’
Ir	0.010	0.127	0.098	0.102
N^N	0.001	0.008	0.006	0.006
N^C	0.009	0.121	0.093	0.098
N^C’	0.009	0.121	0.093	0.097

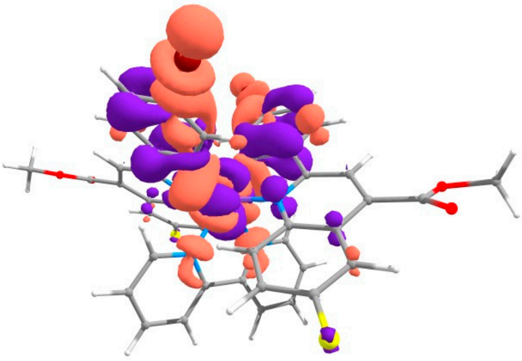

$S_0 \rightarrow S_4$

Donor	Acceptor			
	Ir	N^N	N^C	N^C’
Ir	0.036	0.100	0.042	0.045
N^N	0.007	0.018	0.008	0.008
N^C	0.060	0.166	0.070	0.075
N^C’	0.059	0.163	0.069	0.074

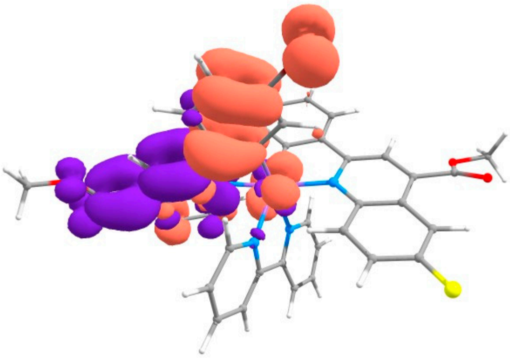

$T_1 \rightarrow S_0$

Donor	Acceptor			
	Ir	N^N	N^C	N^C’
Ir	0.006	0.000	0.032	0.001
N^N	0.002	0.000	0.009	0.000
N^C	0.145	0.008	0.765	0.028
N^C’	0.001	0.000	0.004	0.000

Complex Ir2-NO_2

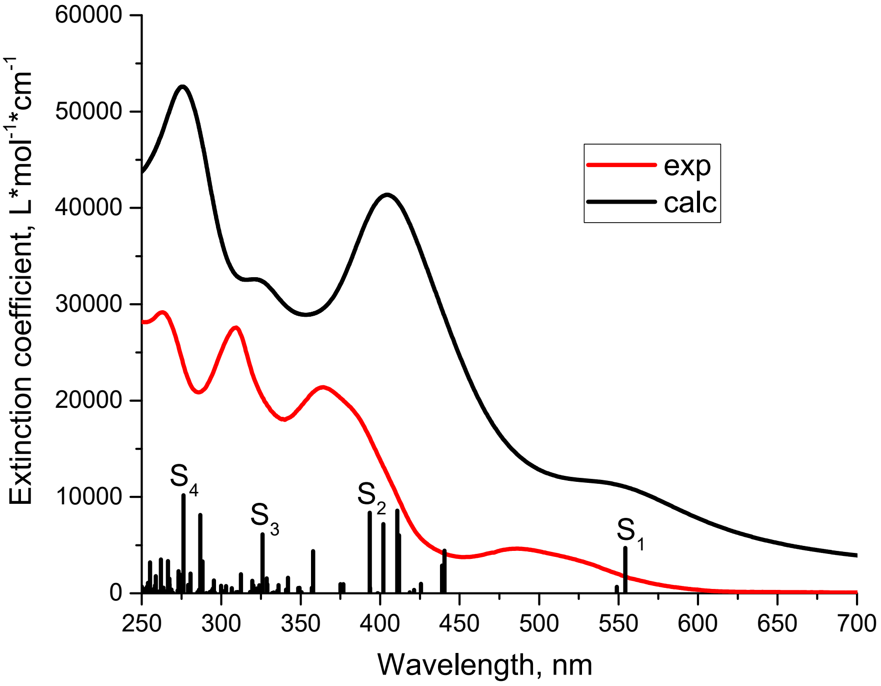

Figure S40. Absorption spectra of **Ir2-NO_2**: experimental (red) and calculated (black) lines with oscillator strengths of electronic transitions (bars).

Table S12. Experimental and calculated absorption maxima (λ), extinction coefficients (ε), oscillator strengths (f) **Ir2-NO₂**.

λ, nm (exp)	ε*10^{-3}, M^{-1}cm^{-1} (exp)	Transitions	λ, nm (calc)	*f* (calc)	Contribution of main NTO pair in transition (%)
262	29	$S_0 \rightarrow S_4$	276	0.28	38
310	28	$S_0 \rightarrow S_3$	326	0.17	38
365	22	$S_0 \rightarrow S_2$	393	0.23	89
488	5	$S_0 \rightarrow S_1$	554	0.13	98

Table S13. The decrease (violet) and increase (terracota) of electron density for most intensive electronic absorption transitions of **Ir2-NO₂**. The data for the corresponding interfragment charge transfer (IFCT) are given below the figures. Diagonal values represent intraligand transitons, off-diagonal values represent a charge transfer from "Donor" to "Acceptor".

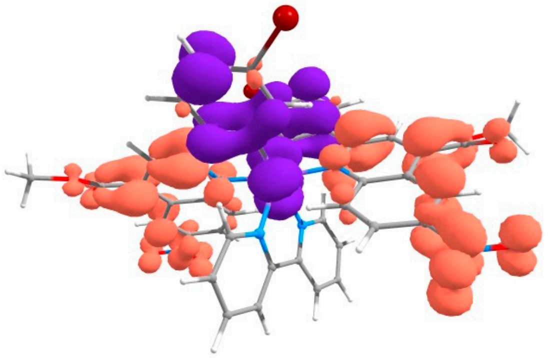

$S_0 \rightarrow S_1$

Donor	Acceptor			
	Ir	N^N	N^C	N^C'
Ir	0.006	0.002	0.186	0.173
N^N	0.000	0.000	0.011	0.010
N^C	0.005	0.002	0.155	0.144
N^C'	0.005	0.002	0.155	0.144

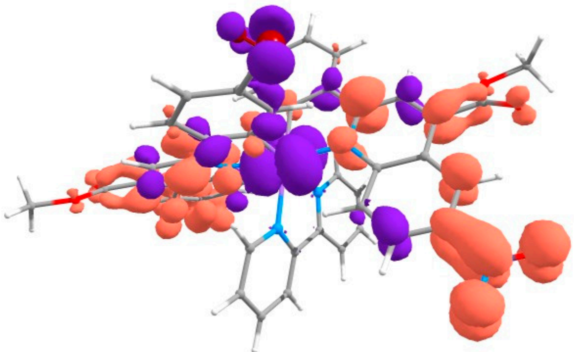

$S_0 \rightarrow S_2$

Donor	Acceptor			
	Ir	N^N	N^C	N^C'
Ir	0.006	0.002	0.222	0.208
N^N	0.001	0.000	0.021	0.019
N^C	0.003	0.001	0.133	0.124
N^C'	0.003	0.001	0.133	0.124

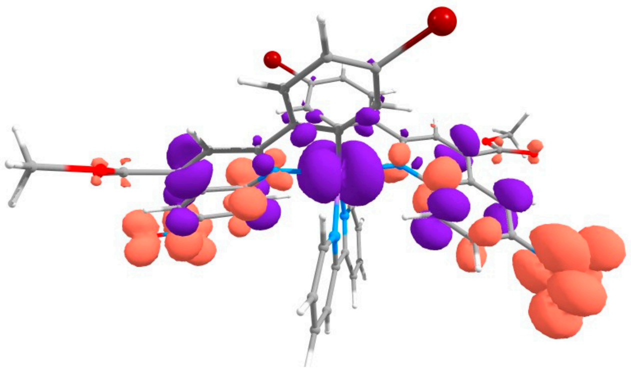

$S_0 \rightarrow S_3$

Donor	Acceptor			
	Ir	N^N	N^C	N^C’
Ir	0.005	0.021	0.128	0.134
N^N	0.001	0.004	0.026	0.027
N^C	0.006	0.024	0.145	0.152
N^C’	0.006	0.024	0.145	0.152

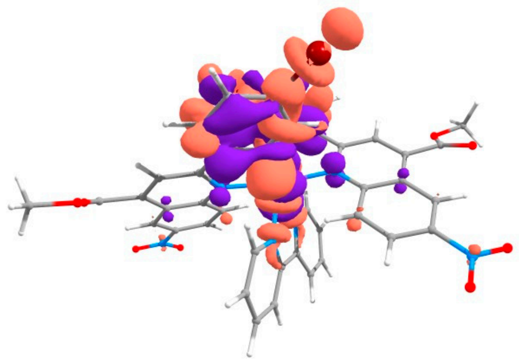

$S_0 \rightarrow S_4$

Donor	Acceptor			
	Ir	N^N	N^C	N^C’
Ir	0.022	0.043	0.087	0.086
N^N	0.002	0.005	0.010	0.010
N^C	0.033	0.066	0.135	0.134
N^C’	0.033	0.066	0.135	0.134

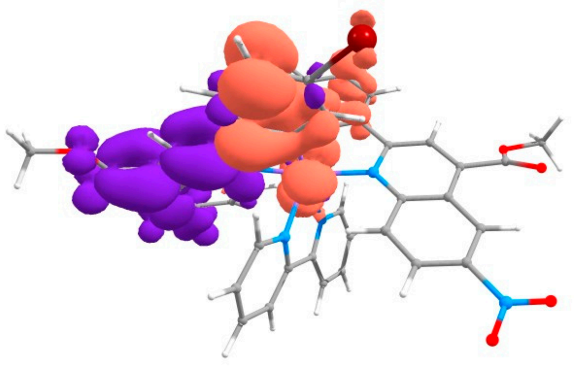

$T_1 \rightarrow S_0$

Donor	Acceptor			
	Ir	N^N	N^C	N^C’
Ir	0.009	0.001	0.004	0.014
N^N	0.003	0.000	0.001	0.004
N^C	0.001	0.000	0.000	0.001
N^C’	0.304	0.019	0.144	0.494

Complex Ir3-F

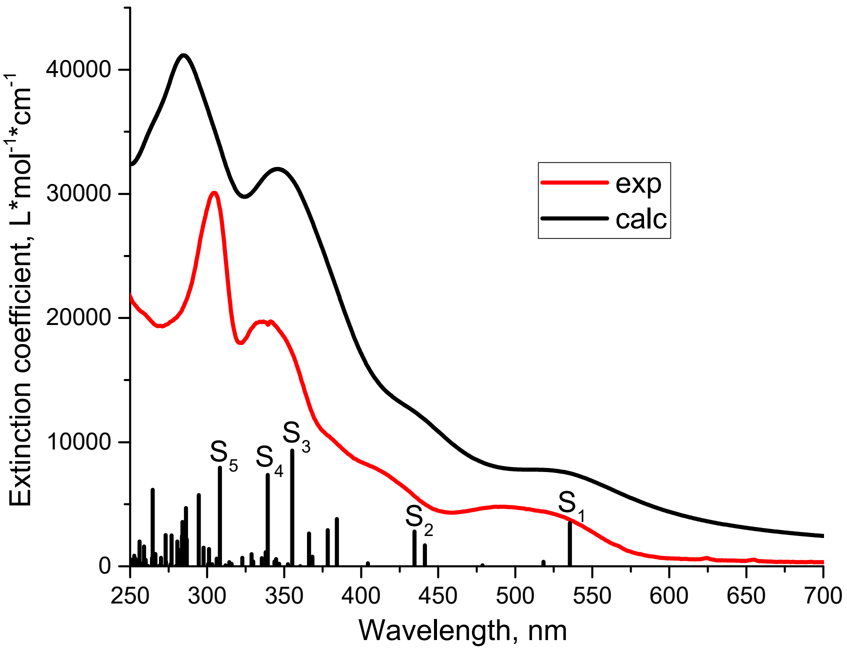

Figure S41. Absorption spectra of **Ir3-F**: experimental (red) and calculated (black) lines with oscillator strengths of electronic transitions (bars).

Table S14. Experimental and calculated absorption maxima (λ), extinction coefficients (ε), oscillator strengths (f) **Ir3-F**.

λ, nm (exp)	ε*10⁻³, M⁻¹cm⁻¹ (exp)	Transitions	λ, nm (calc)	*f* (calc)	Contribution of main NTO pair in transition (%)
306	30	$S_0 \rightarrow S_5$	308	0.22	88
336	20	$S_0 \rightarrow S_4$	339	0.20	87
		$S_0 \rightarrow S_3$	355	0.25	67
408	8	$S_0 \rightarrow S_2$	435	0.08	97
498	5	$S_0 \rightarrow S_1$	535	0.09	97

Table S15. The decrease (violet) and increase (terracota) of electron density for most intensive electronic absorption transitions of **Ir3-F**. The data for the corresponding interfragment charge transfer (IFCT) are given below the figures. Diagonal values represent intraligand transitons, off-diagonal values represent a charge transfer from "Donor" to "Acceptor".

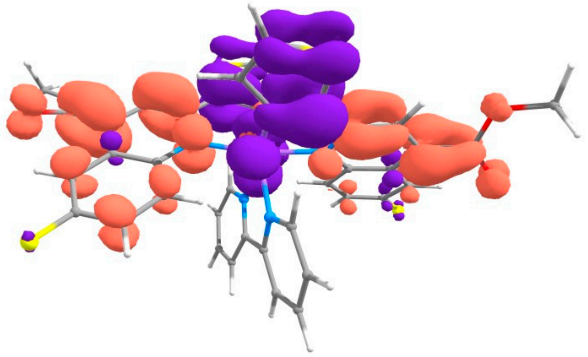

$S_0 \rightarrow S_1$

Donor	Acceptor			
	Ir	N^N	N^C	N^C'
Ir	0.007	0.003	0.163	0.102
N^N	0.000	0.000	0.009	0.006
N^C	0.009	0.004	0.211	0.131
N^C'	0.009	0.004	0.209	0.130

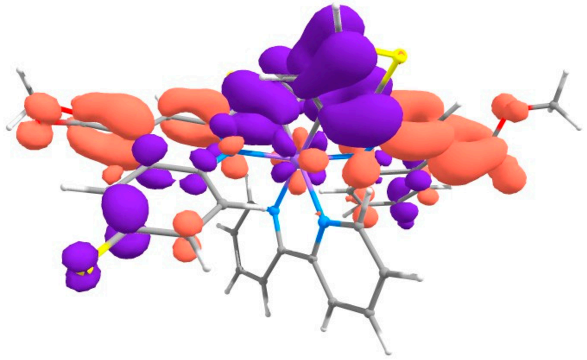

$S_0 \rightarrow S_2$

Donor	Acceptor			
	Ir	N^N	N^C	N^C'
Ir	0.002	0.001	0.018	0.029
N^N	0.000	0.000	0.003	0.004
N^C	0.018	0.007	0.170	0.276
N^C'	0.018	0.007	0.170	0.275

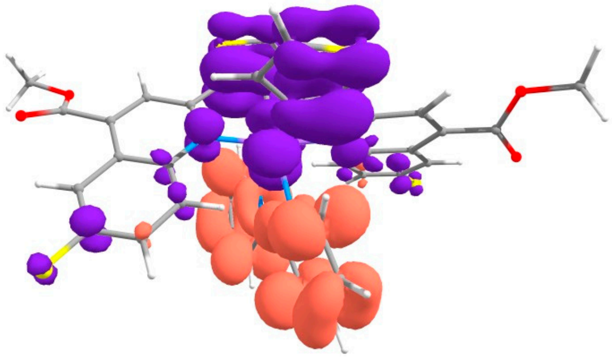

$S_0 \rightarrow S_3$

Donor	Acceptor			
	Ir	N^N	N^C	N^C'
Ir	0.011	0.176	0.064	0.059
N^N	0.001	0.013	0.005	0.004
N^C	0.012	0.190	0.070	0.064
N^C'	0.012	0.189	0.069	0.063

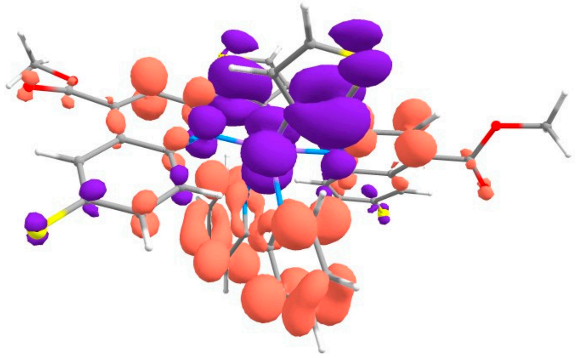

$S_0 \rightarrow S_4$

Donor	Acceptor			
	Ir	N^N	N^C	N^C'
Ir	0.005	0.121	0.080	0.077
N^N	0.000	0.007	0.005	0.004
N^C	0.006	0.151	0.099	0.096
N^C'	0.006	0.150	0.099	0.096

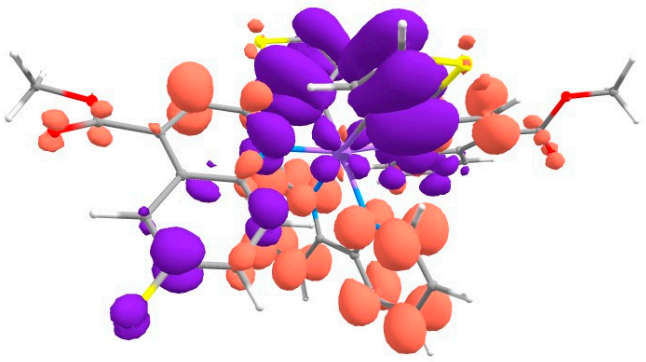

$S_0 \rightarrow S_5$

Donor	Acceptor			
	Ir	N^N	N^C	N^C’
Ir	0.002	0.020	0.016	0.015
N^N	0.002	0.017	0.013	0.012
N^C	0.018	0.171	0.135	0.128
N^C’	0.018	0.171	0.135	0.127

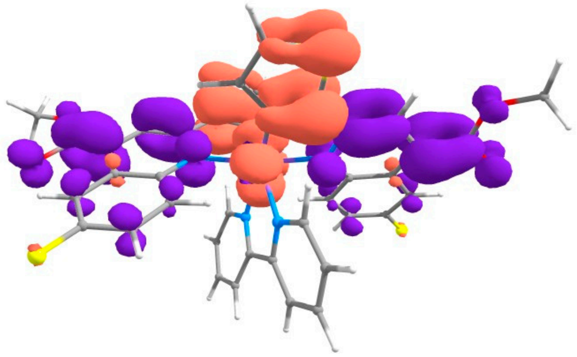

$T_1 \rightarrow S_0$

Donor	Acceptor			
	Ir	N^N	N^C	N^C’
Ir	0.007	0.000	0.011	0.011
N^N	0.003	0.000	0.005	0.005
N^C	0.113	0.008	0.179	0.179
N^C’	0.113	0.008	0.179	0.179

Complex Ir4-F

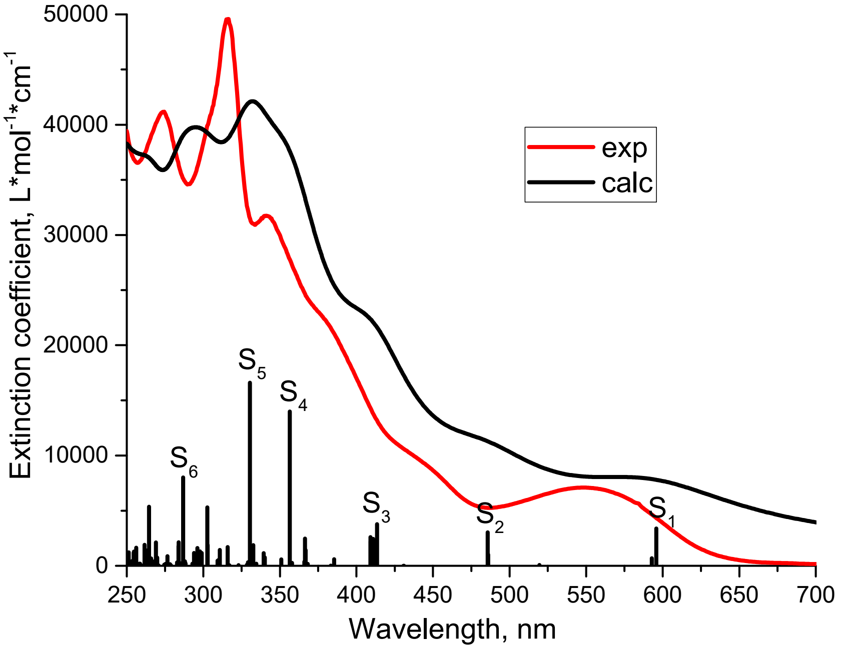

Figure S42. Absorption spectra of **Ir4-F**: experimental (red) and calculated (black) lines with oscillator strengths of electronic transitions (bars).

Table S16. Experimental and calculated absorption maxima (λ), extinction coefficients (ε), oscillator strengths (f) **Ir4-F**.

λ, nm (exp)	ε*10^{-3}, $M^{-1}cm^{-1}$ (exp)	Transitions	λ, nm (calc)	*f* (calc)	Contribution of main NTO pair in transition (%)
272	41	$S_0 \rightarrow S_6$	287	0.22	66
316	49	$S_0 \rightarrow S_5$	330	0.45	90
343	32	$S_0 \rightarrow S_4$	356	0.38	62
383	22	$S_0 \rightarrow S_3$	413	0.10	95
438	10	$S_0 \rightarrow S_2$	486	0.08	99
554	7	$S_0 \rightarrow S_1$	596	0.09	98

Table S17. The decrease (violet) and increase (terracota) of electron density for most intensive electronic absorption transitions of **Ir4-F**. The data for the corresponding interfragment charge transfer (IFCT) are given below the figures. Diagonal values represent intraligand transitons, off-diagonal values represent a charge transfer from "Donor" to "Acceptor".

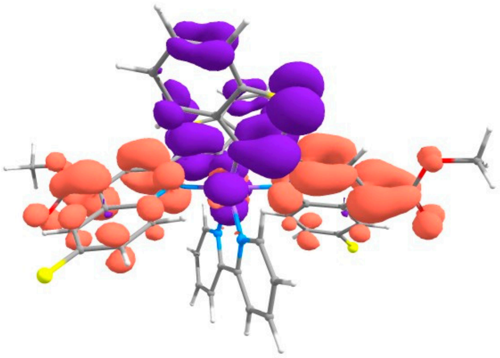

$S_0 \rightarrow S_1$

Donor	Acceptor			
	Ir	N^N	N^C	N^C'
Ir	0.006	0.002	0.091	0.091
N^N	0.000	0.000	0.005	0.005
N^C	0.013	0.004	0.192	0.192
N^C'	0.013	0.004	0.192	0.192

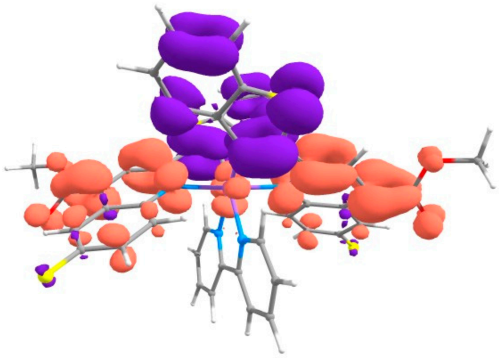

$S_0 \rightarrow S_2$

Donor	Acceptor			
	Ir	N^N	N^C	N^C'
Ir	0.001	0.000	0.008	0.008
N^N	0.000	0.000	0.003	0.003
N^C	0.019	0.010	0.229	0.229
N^C'	0.019	0.010	0.229	0.229

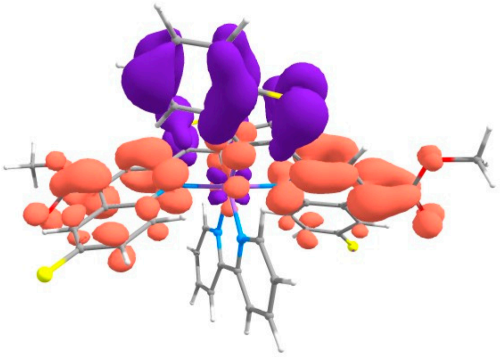

S0→S3

Donor	Acceptor			
	Ir	N^N	N^C	N^C’
Ir	0.001	0.001	0.015	0.015
N^N	0.000	0.000	0.003	0.003
N^C	0.020	0.010	0.236	0.236
N^C’	0.018	0.010	0.216	0.216

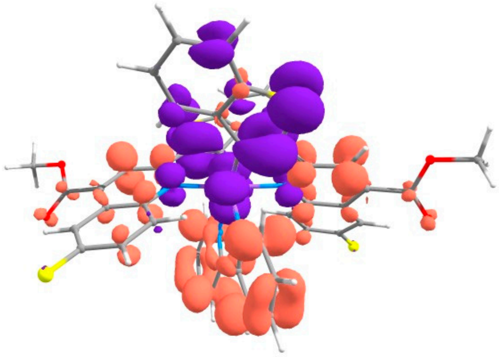

S0→S4

Donor	Acceptor			
	Ir	N^N	N^C	N^C’
Ir	0.004	0.061	0.080	0.080
N^N	0.000	0.005	0.006	0.006
N^C	0.007	0.102	0.135	0.135
N^C’	0.007	0.102	0.135	0.135

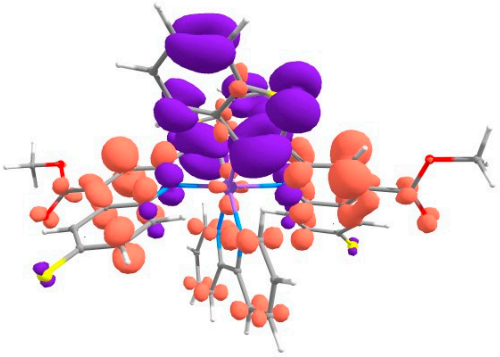

S0→S5

Donor	Acceptor			
	Ir	N^N	N^C	N^C’
Ir	0.001	0.003	0.010	0.010
N^N	0.000	0.001	0.003	0.003
N^C	0.015	0.054	0.208	0.208
N^C’	0.015	0.054	0.208	0.208

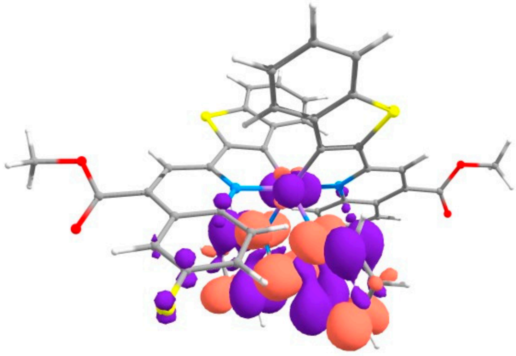

S0→S6

Donor	Acceptor			
	Ir	N^N	N^C	N^C’
Ir	0.004	0.108	0.014	0.014
N^N	0.019	0.475	0.061	0.061
N^C	0.004	0.094	0.012	0.012
N^C’	0.004	0.096	0.012	0.012

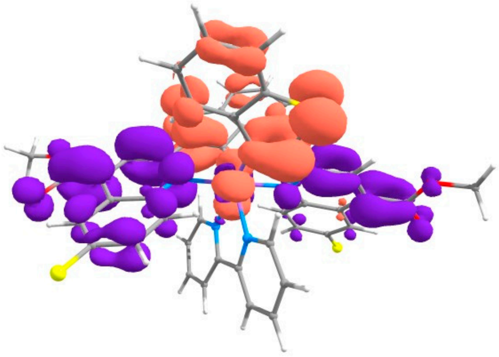

T1→S0

Donor	Acceptor			
	Ir	N^N	N^C	N^C’
Ir	0.006	0.000	0.015	0.015
N^N	0.002	0.000	0.005	0.005
N^C	0.059	0.003	0.151	0.149
N^C’	0.096	0.006	0.247	0.242

Complex Ir4-NO$_2$

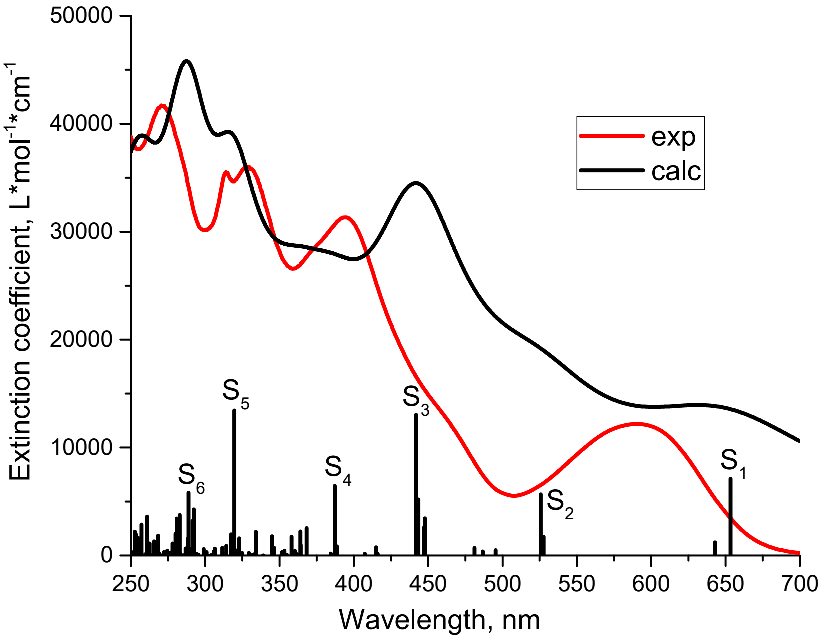

Figure S43. Absorption spectra of **Ir4-NO$_2$**: experimental (red) and calculated (black) lines with oscillator strengths of electronic transitions (bars).

Table S18. Experimental and calculated absorption maxima (λ), extinction coefficients (ε), oscillator strengths (f) **Ir4-NO₂**.

λ, nm (exp)	$\varepsilon \cdot 10^{-3}$, $M^{-1}cm^{-1}$ (exp)	Transitions	λ, nm (calc)	*f* (calc)	Contribution of main NTO pair in transition (%)
270	42	$S_0 \rightarrow S_6$	289	0.16	35
315	35	$S_0 \rightarrow S_5$	320	0.36	60
328	36				
395	31	$S_0 \rightarrow S_4$	387	0.18	54
		$S_0 \rightarrow S_3$	442	0.35	91
458	14	$S_0 \rightarrow S_2$	526	0.15	95
590	12	$S_0 \rightarrow S_1$	653	0.19	99

Table S19. The decrease (violet) and increase (terracota) of electron density for most intensive electronic absorption transitions of **Ir4-NO₂**. The data for the corresponding interfragment charge transfer (IFCT) are given below the figures. Diagonal values represent intraligand transitons, off-diagonal values represent a charge transfer from "Donor" to "Acceptor".

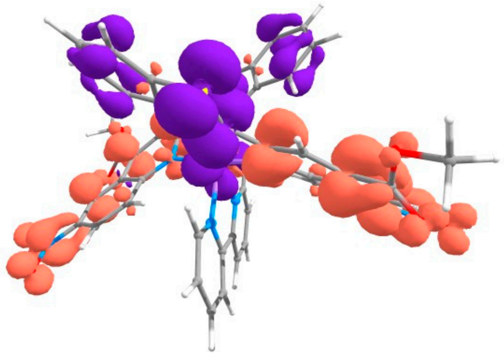

$S_0 \rightarrow S_1$

Donor	Acceptor			
	Ir	N^N	N^C	N^C'
Ir	0.004	0.001	0.086	0.087
N^N	0.000	0.000	0.005	0.005
N^C	0.009	0.003	0.196	0.198
N^C'	0.009	0.003	0.196	0.198

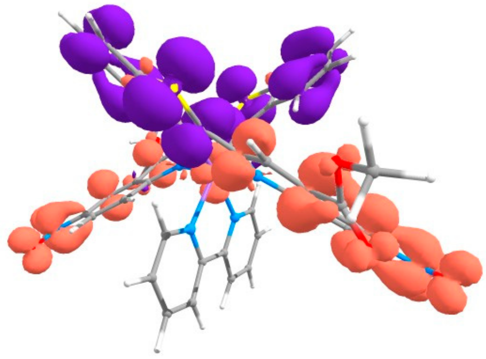

$S_0 \rightarrow S_2$

Donor	Acceptor			
	Ir	N^N	N^C	N^C'
Ir	0.000	0.000	0.007	0.007
N^N	0.000	0.000	0.003	0.003
N^C	0.015	0.006	0.235	0.232
N^C'	0.015	0.006	0.235	0.232

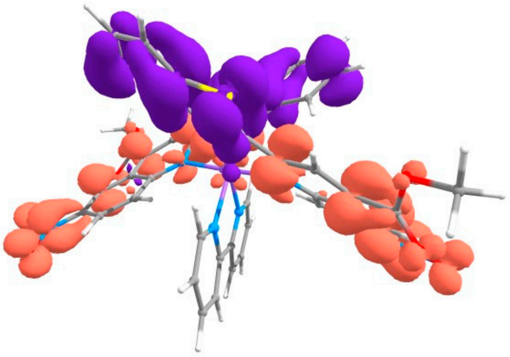

$S_0 \rightarrow S_3$

Donor	Acceptor			
	Ir	N^N	N^C	N^C'
Ir	0.000	0.000	0.010	0.010
N^N	0.000	0.000	0.001	0.001
N^C	0.009	0.003	0.237	0.240
N^C'	0.009	0.003	0.237	0.239

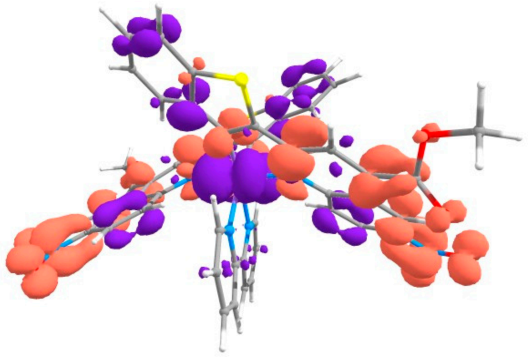

$S_0 \rightarrow S_4$

Donor	Acceptor			
	Ir	N^N	N^C	N^C'
Ir	0.009	0.015	0.187	0.187
N^N	0.001	0.002	0.023	0.023
N^C	0.006	0.010	0.128	0.129
N^C'	0.006	0.010	0.128	0.129

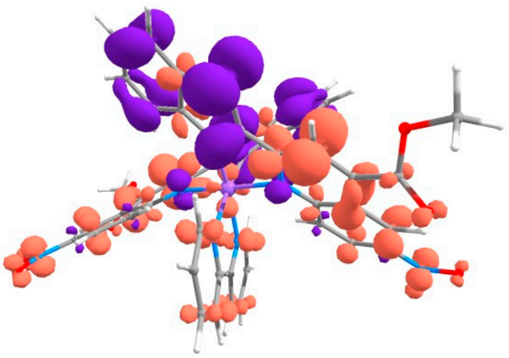

$S_0 \rightarrow S_5$

Donor	Acceptor			
	Ir	N^N	N^C	N^C'
Ir	0.003	0.012	0.058	0.058
N^N	0.001	0.003	0.012	0.012
N^C	0.010	0.038	0.185	0.184
N^C'	0.010	0.038	0.185	0.184

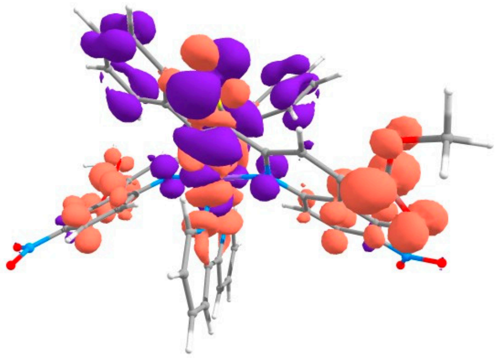

$S_0 \rightarrow S_6$

Donor	Acceptor			
	Ir	N^N	N^C	N^C'
Ir	0.011	0.027	0.031	0.031
N^N	0.030	0.076	0.086	0.086
N^C	0.030	0.075	0.084	0.084
N^C'	0.030	0.075	0.084	0.084

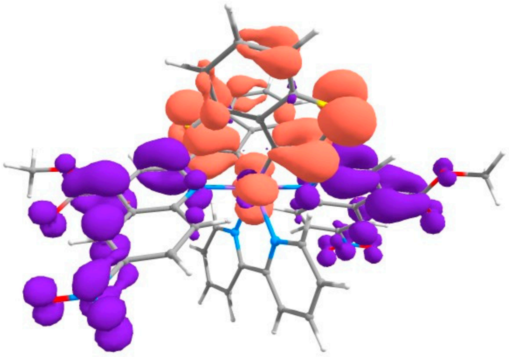

$T_1 \rightarrow S_0$

Donor	Acceptor			
	Ir	N^N	N^C	N^C’
Ir	0.004	0.000	0.012	0.012
N^N	0.001	0.000	0.003	0.003
N^C	0.076	0.004	0.201	0.201
N^C’	0.075	0.004	0.201	0.201
